# Integrating multi-omics data with network modeling to characterize *Pseudomonas aeruginosa* persister cell metabolism

**DOI:** 10.1128/jb.00373-25

**Published:** 2026-07-20

**Authors:** Joseph M. Ficarrotta, Anna S. Blazier, Aline Metris, Sidney L. Rhodes, Glynis L. Kolling, Jason A. Papin

**Affiliations:** 1 Department of Biomedical Engineering, University of Virginia 174483, Charlottesville, Virginia, USA; 2 Unilever Safety, Environmental & Regulatory Sciences (SERS) group, Sharnbrook, United Kingdom; 3 Division of Infectious Diseases and International Health, School of Medicine, University of Virginia 12349, Charlottesville, Virginia, USA; 4 Department of Biochemistry & Molecular Genetics, University of Virginia 174482, Charlottesville, Virginia, USA; University of California San Francisco, San Francisco, California, USA

**Keywords:** metabolism, modeling, multi-omics, persister

## Abstract

*Pseudomonas aeruginosa* is a gram-negative bacterial pathogen capable of forming antimicrobial-tolerant subpopulations known as persister cells. These cells are transient phenotypic variants that can tolerate antimicrobial treatment and have been associated with chronic infections and the development of antibiotic resistance. While persister cells are classically associated with reduced metabolic activity, the characteristics of their metabolism are not well understood, especially in the context of biocides. In this work, we performed an experimental and computational system-level analysis to characterize the metabolic state of biocide persister cells. To accomplish this, we conducted in-depth profiling of both wild-type and persister samples of *P. aeruginosa* with transcriptomic sequencing and metabolomic analyses. These analyses revealed a distinct metabolic repertoire in biocide persister cells, marked by an upregulation in genes associated with activity in central metabolism. Integration of both the transcriptomic data set with a *P. aeruginosa* genome-scale metabolic network reconstruction (GENRE) provided condition-specific models, which were used to identify metabolic reactions and genes that differentiated the persister phenotype from the untreated. Experimental testing of model predictions revealed metabolic functions, such as pyrimidine synthesis and methionine recycling, which could serve as potential targets for inhibiting persister cell formation.

**IMPORTANCE:**

Bacterial persister cells represent a transient subpopulation that can survive lethal antimicrobial treatments and are a major barrier to eliminating chronic infections, yet their formation and maintenance of this state remain poorly understood. By examining *Pseudomonas aeruginosa* treated with the industrial biocide benzisothiazolinone, this study reveals that persister cells retain active metabolism and exhibit a metabolic program distinct from that of untreated cells. We integrated transcriptomics, metabolomics, and genome-scale metabolic modeling to identify specific metabolic pathways, notably pyrimidine biosynthesis and methionine recycling, that are critical for persister survival. These findings provide insight into persister cell biology and highlight metabolism as a promising target for strategies aimed at preventing or eliminating these tolerant bacterial subpopulations.

## INTRODUCTION

Bacterial persistence is a transient, phenotypic state marked by the ability to withstand antimicrobial treatment (1). When a culture is exposed to high doses of antimicrobials, a majority of the cells rapidly die; however, a subpopulation, known as persister cells, remains and tolerates treatment. Upon removal of the antimicrobial pressure, persister cells can then switch into a normal phenotype and repopulate the culture (2). Because of their transient, tolerant nature, persister cells have been implicated in the recalcitrant nature of chronic infections (3, 4). Furthermore, the presence of persister cells may contribute to the development of antimicrobial resistance (5). Despite their clinical and industrial relevance, relatively little is known about persister cell formation and maintenance of this state across different bacterial species (6).

Traditionally, persister cells are thought to tolerate antimicrobial treatment due to a reduced metabolic state (7). However, the specifics of persister cell metabolism are poorly understood. While some studies demonstrate that persistence is linked to arrested cell growth (8, 9), others indicate that dormancy is not a prerequisite for persistence (10, 11). Furthermore, studies have shown that inactivation of the tricarboxylic acid cycle can differentially modulate persister levels depending on the organism being studied (12, 13). Nevertheless, the metabolism of persister cells has been shown to be important for their ability to tolerate antimicrobial treatment (14–17). While individual metabolic genes have been linked to the persister phenotype (18), robust analyses of persister cell metabolism are lacking (11). A system-level analysis of persister cell metabolism would enhance our understanding of the persister cell metabolic program and enable identification of promising targeting strategies to eliminate the persister phenotype.

Benzisothiazolinone (BIT) is a biocide that is used in a wide range of products from detergents to paints. BIT can diffuse across the bacterial cell membrane and react with thiols from cysteines of proteins’ active sites, blocking their enzymatic activity (19). This action results in free radical production as well as impaired cell growth, respiration, and ATP production, leading to cell death (20); however, some cells may survive (21). Previous studies have characterized persister cells after biocidal treatments, but the mode of action at the molecular level is biocide and micro-organism-specific (21–24), and it is not clear how *Pseudomonas aeruginosa* metabolism allows it to survive BIT treatment (25).

**Fig 1:**
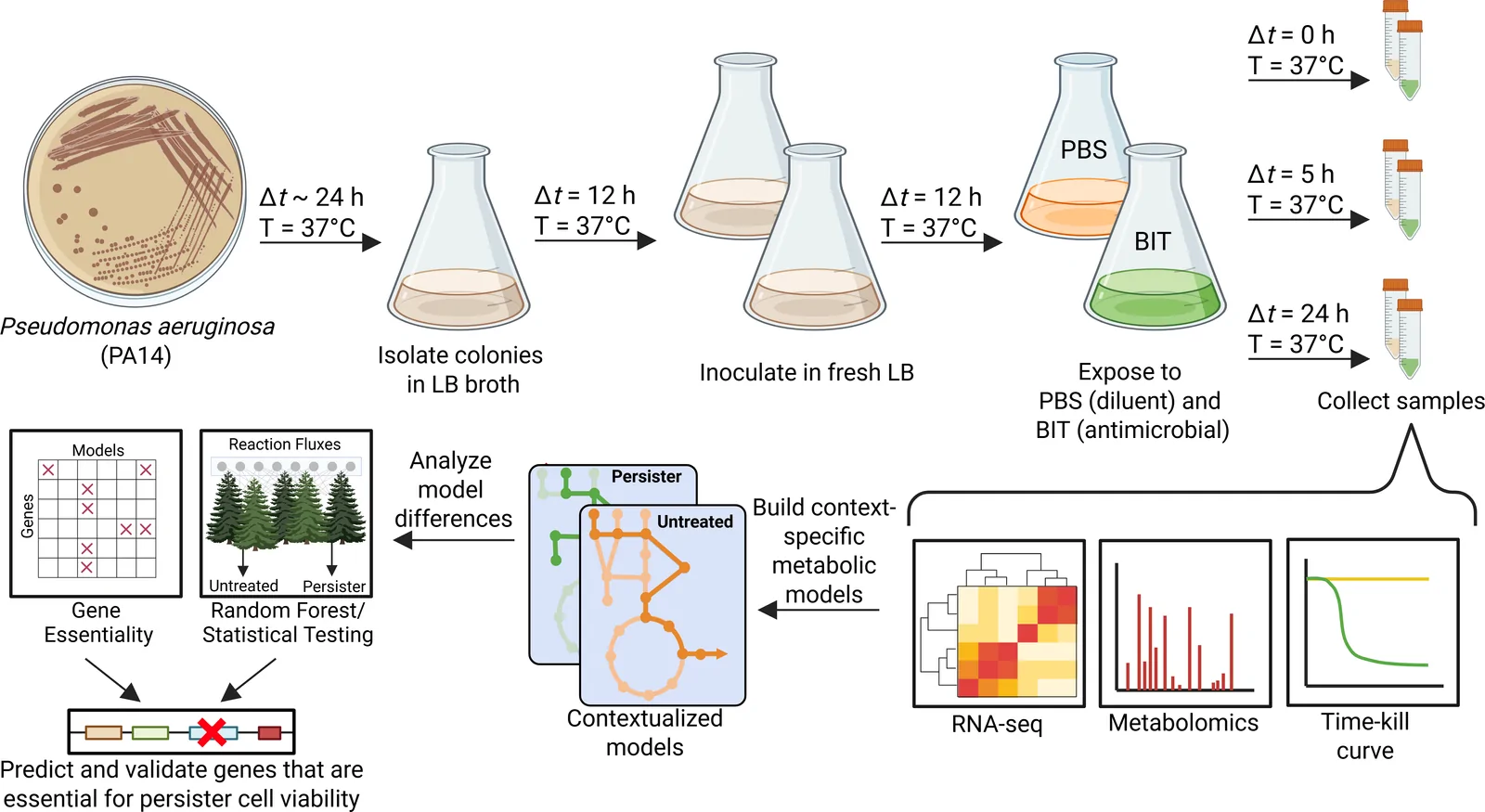
Experimental and computational systems biology approach for characterizing the metabolism of *Pseudomonas aeruginosa* PA14 persister cells. In this study, we streaked *Pseudomonas aeruginosa* (PA14) onto an LB agar plate. Individual colonies were inoculated into a 50 mL flask containing 10 mL of LB and grown overnight. The overnight cultures were diluted in fresh LB in 500 mL flasks to an optical density (OD)_600_ of 0.01 for a total volume of 50 mL and grown for 12 h to reach the stationary phase. The cultures were subsequently exposed to BIT by removing 5 mL of the culture and adding 5 mL of antimicrobial at various concentrations (phosphate-buffered saline [PBS] for untreated). The flasks were returned to the incubator for 24 h. Samples were collected at 0 h, 5 h, and 24 h after exposure to generate a time-kill curve. Samples were also collected to run metabolomics and RNA-seq and assess metabolic and functional changes in the persister state. The RNA-seq data were then used to build context-specific metabolic models to simulate the metabolism of persister and untreated cells. A random forest classifier and a Mann–Whitney U test were used to differentiate reaction fluxes in the persister group from those in the untreated group. Additionally, *in silico* gene essentiality was performed to identify the differences in essential genes between the persister and untreated models. The genes and reactions that best differentiate the persister models from the untreated models were then experimentally validated for their essentiality in the persister state by repeating the persister isolation on PA14 transposon mutants.

In this work, we performed a system-level analysis to quantitatively characterize the metabolic state of biocide persister cells (Fig. 1). We collected transcriptomics and metabolomics data from persister cells of the gram-negative, opportunistic pathogen *P. aeruginosa* exposed to the biocide benzisothiazolinone. To facilitate the system-level analysis of this persister cell metabolism, we employed genome-scale metabolic network reconstructions (GENREs), which are computational modeling frameworks that capture the genotype-to-phenotype relationship and enable the study of metabolic capabilities of an organism of interest. Through integration of transcriptomic and metabolomic data sets with a *P. aeruginosa* GENRE (26), we generated condition-specific metabolic models of the persister and untreated states. We then used these models to suggest targets of persister cell metabolism to inhibit persister cell formation. We validated these predictions by performing time-kill assays on transposon mutants of the desired genes and comparing the resulting persister cell count to that of the PA14 wild-type strain.

## RESULTS

### Persister cells enable *Pseudomonas aeruginosa* to tolerate treatment with BIT

**Fig 2:**
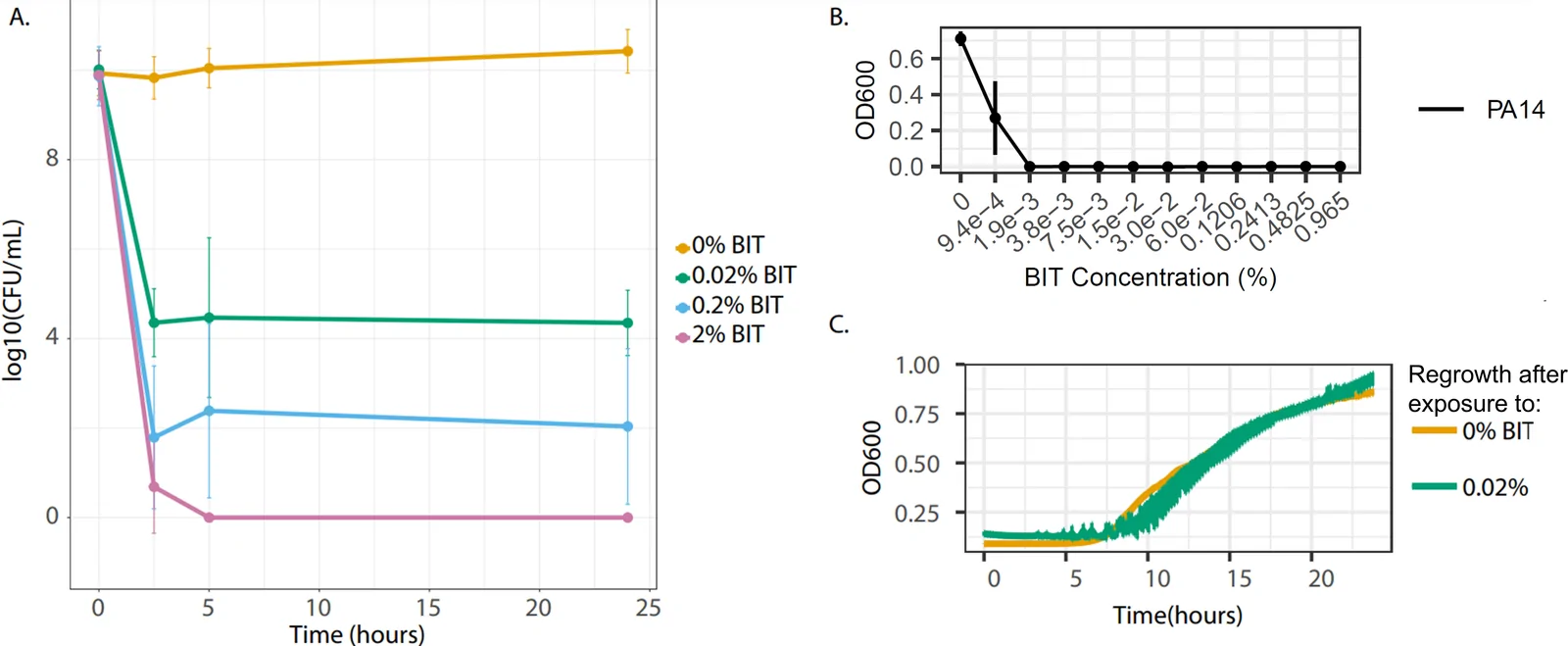
Killing of *P. aeruginosa* PA14 cultures by BIT over time. (**A**) Time-kill curve of *P. aeruginosa* cultures exposed to the antimicrobial BIT. Stationary-phase cultures of *P. aeruginosa* PA14 were exposed to various concentrations of BIT for 24 h. The number of CFUs in each culture was counted immediately before exposure at 0 h and 2.5 h, 5 h, and 24 h after exposure. Untreated cultures exposed to PBS (yellow line; 0% BIT); cultures exposed to 0.02% BIT (green line; 10× the MIC); cultures exposed to 0.2% BIT (blue line; 100× the MIC); and cultures exposed to 2% BIT (pink line; 1,000× the MIC). Each kill curve is comprised of three biological replicates (*n* = 3). (**B**) Dose–response curve of *P. aeruginosa* treated with BIT. Overnight cultures of PA14 were diluted and exposed to various concentrations of BIT. Growth was measured 24 h after inoculation via OD_600_ measurements. (**C**) Re-growth of untreated and 0.02% BIT-treated *P. aeruginosa* samples. Untreated and 0.02% BIT-treated samples were diluted to the same starting CFU concentration in LB media. Growth was measured for 24 h using OD_600_ measurements.

We first identified the minimum inhibitory concentration (MIC) of *P. aeruginosa* to BIT as 0.002% using broth microdilution (27) (Fig. 2B). To determine whether *P. aeruginosa* is able to tolerate treatment with BIT due to the presence of persister cells, we performed a standard time-kill assay (Fig. 2A). Stationary-phase cultures of *P. aeruginosa* were exposed to various concentrations of BIT corresponding to 0×, 10×, 100×, and 1,000× the MIC of BIT against *P. aeruginosa*. Colony-forming units (CFUs) were measured in the culture immediately before exposure (0 h), and 2.5 h, 5 h, and 24 h after exposure. It is important to note that BIT treatment does not necessarily generate persister cells. Persister cells naturally accumulate in the stationary phase, where nutrient limitation and slowed metabolism naturally enrich for dormant, drug-tolerant cells (28).

The time-kill curves of the 10× and 100× MIC cultures exhibited a characteristic biphasic behavior marked by an initial high rate of killing followed by a plateau in killing. The biphasic behavior is indicative of the presence of persister cells in the culture (29). In contrast, the 1,000× MIC culture exhibited complete killing by 5 h after exposure. To statistically compare CFU counts between BIT concentrations at each time point, CFU values were analyzed using a non-parametric Kruskal–Wallis test followed by Dunn’s post hoc test with Benjamini–Hochberg correction (Table S3). These analyses confirmed significant differences in bacterial survival between BIT concentrations, particularly at the 5-h and 24-h timepoints where CFU counts diverged the most. By the 5-h mark, the untreated cells had significantly higher CFU counts than the BIT-treated cells (*P*_adj_ ≤ 0.05). Persister cells were identified operationally based on their ability to survive lethal BIT treatment well above the MIC; no physical separation of live and dormant cells was performed. This functional definition of persistence, determined by survival rather than prior isolation, is a widely accepted method for quantifying persister populations in bacterial cultures (29, 30). To further confirm that the cells that survived treatment are indeed persister cells, we measured the re-growth of the untreated and 0.02% BIT (10× MIC) conditions (Fig. 2C). When the samples were diluted to the same starting CFU concentration, there was not an appreciable difference in the growth profiles of the two samples, further confirming that persister cells enable *P. aeruginosa* to tolerate treatment with the antimicrobial BIT. To rule out the possibility that cells surviving BIT treatment were not a permanently resistant sub-population, we performed MIC assays comparing the untreated parent population with multiple independent colonies that survived the 0.02% BIT treatment. As expected for a persister phenotype, the MIC of the BIT survivors was identical to that of the untreated control cells (0.004% BIT for both groups) (Fig. S1). The new MICs were slightly higher for both the untreated and persister cells than the MICs before the experiment. The uniform, low-level increase in MICs for both the survivors and the untreated parent group reflects a well-documented phenotypic transition of *P. aeruginosa* into the stationary phase, during which starvation and cell-density signals trigger a transient, collective upregulation of global stress responses and multidrug efflux pumps (31–33). Since the BIT-survivor group did not show any additional increase in MIC over the untreated group, they represent true phenotypic persister cells.

### The transcriptional state of persister cells is distinct from that of untreated cells

**Fig 3:**
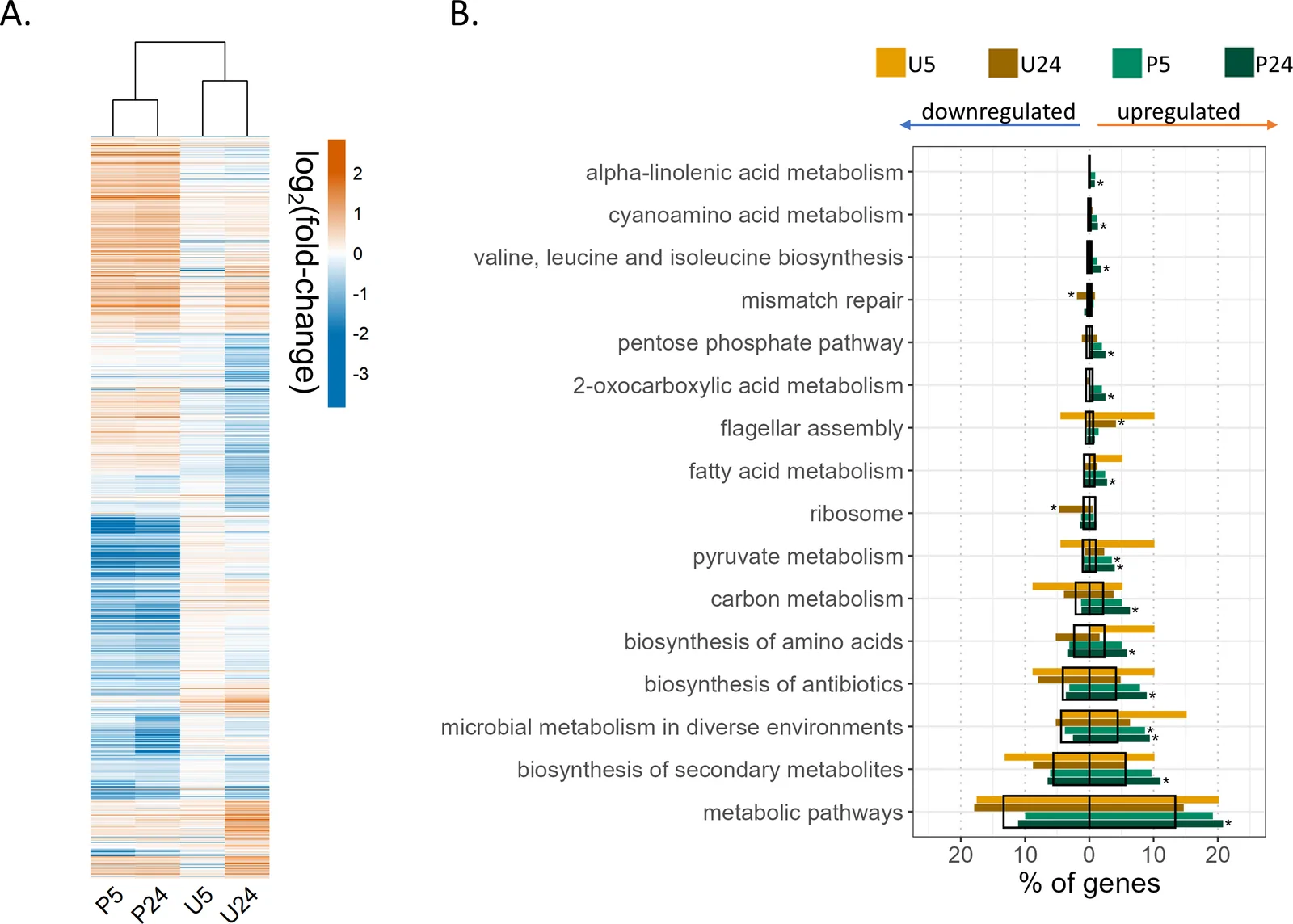
RNA-sequencing analysis of *P. aeruginosa* persister cells. Samples were collected for RNA sequencing from the untreated and persister conditions (0× and 10× the MIC, respectively) immediately before exposure and 5 h and 24 h after exposure. (**A**) Differential expression analysis was performed by comparing the 5-h and 24-h samples to the time 0 samples. The heatmap shows the log_2_(fold-change) for genes significantly differentially expressed in at least one group relative to the time 0 samples. (**B**) Gene enrichment analysis based on KEGG pathway classification was performed on the significantly increased and decreased genes for each comparison. Black boxes represent the fraction of the entire genome represented in the categories. Asterisks denote significant pathway enrichment (*P*_adj_ ≤ 0.05, hypergeometric test after Bonferroni correction).

We were then interested in comparing the transcriptional state of *P. aeruginosa* persister cells in the presence of BIT to that of untreated cells. We collected samples for RNA sequencing from the 0× and 10× MIC conditions (henceforth referred to as the untreated and persister conditions, respectively) immediately before exposure (i.e., 0 h) as well as 5 and 24 h after exposure. Differential expression analysis of the RNA-sequencing data revealed that persister cells are in a distinct transcriptional state from untreated cells (Fig. 3). When comparing the 5-h and 24-h samples of the persister and untreated conditions to the 0-h samples, the two persister conditions clustered separately from the untreated conditions (Fig. 3A). Indeed, the persister conditions are marked by distinct regions of increased and decreased expression compared to the untreated conditions.

To ensure that the captured transcriptomic signals uniquely reflected the physiology of viable, active *P. aeruginosa* persisters rather than residual mRNA from a vast excess of dying non-persisters, we evaluated cell morphology using live/dead staining and fluorescence microscopy 24 h post-BIT treatment. This validation is critical, as recent studies in *E. coli* and *Salmonella* have noted that non-persister cells can temporarily remain morphologically intact and structurally stable during and after initial antibiotic exposure windows (34, 35). Microscopy analysis revealed that while the number of propidium iodide (PI)-positive (dead) cells exceeded that of viable cells, these dead cells were distinctly characterized by substantial cell shrinkage, structural distortion, and extensive leaking of intracellular signal compared to the dead cells in the untreated population (Fig. S2). This widespread loss of cell envelope integrity directly exposes internal cellular contents, including nucleic acids, to periplasmic and extracellular nucleases. While previously reported studies found that reactive oxygen species (ROS) accumulation occurred during antimicrobial treatment windows of 4 h or less (34), the 24-h BIT treatment condition provides an extended timeline that allows for self-amplifying ROS cascades to run to completion, clearing the non-persister population long before sampling. Given that the half-life of most bacterial mRNA is exceptionally short, typically 2–4 min (36), and the vast majority of non-persister cell death occurs within the first hour of treatment, any mRNA from these structurally compromised cells would be degraded by the time samples are collected. Consequently, we interpret robust signatures in the transcriptomic data to capture the active response of true viable persisters.

We then assessed the overlap between the significantly increased genes for the persister and untreated conditions. The two persister conditions (5 h and 24 h) shared the largest number of genes with increased expression relative to the 0-h samples. The untreated condition at 24 h had the second-largest increase in expression compared to the 0-h samples (Fig. S3A). The same comparison was done for decreased expression across the conditions. Similar to the commonalities with increased gene expression, the two persister conditions (5 h and 24 h) also shared the largest number of genes with decreased expression relative to the 0-h samples (Fig. S3B). Overall, there were 519 common genes differentially expressed in the two persister conditions relative to the 0-h samples that were not differentially expressed in the untreated conditions at 5 h or 24 h.

To get a sense for system-level changes in the expression data, we performed gene enrichment analysis based on KEGG pathway classification systems (Fig. 3B). Interestingly, the increased gene expression in the persister conditions revealed significant enrichment of several metabolic pathways, including carbon metabolism and the biosynthesis of secondary metabolites. In contrast, the increased expression in the untreated samples was only enriched for flagellar assembly (both timepoints). The only metabolic pathways significantly enriched for in the untreated samples were the downregulation of mismatch repair at 24 h and the upregulation of flagellar assembly at 24 h (*P*_adj_ ≤ 0.01). As demonstrated in Fig. 3B, some of the gene sets had a high percentage of genes in specific sets, especially for the untreated samples at 5 h. The untreated samples at 5 h only had 20 total significantly upregulated and 23 significantly downregulated genes, while the persister samples had 421 upregulated and 457 downregulated genes. Because the hypergeometric test used in the term enrichment considers both the number of genes in the subset and the size of the universe, the small absolute number of differentially expressed genes (DEGs) in the untreated group at 5 h results in a lower statistical power to detect enrichment. In contrast, the larger number of differentially expressed genes in the other groups allows the test to achieve significance, even if the percentage is comparable or slightly lower. Overall, the persister samples had a higher number of differentially expressed genes relative to the untreated samples, demonstrating a greater shift in total metabolism and allowing for more significant pathway enrichment.

These enrichment results suggest an overall shift in the metabolic state of persister cells compared to untreated samples. Furthermore, enrichment of metabolic pathway expression in the persister conditions suggests that persister cells are in an active metabolic state. Interestingly, few pathways were significantly enriched for genes with decreased expression, suggesting that most pathways maintain a baseline level of activity regardless of the treatment conditions.

### The metabolite footprint of persister samples is different from that of untreated and dead samples

**Fig 4:**
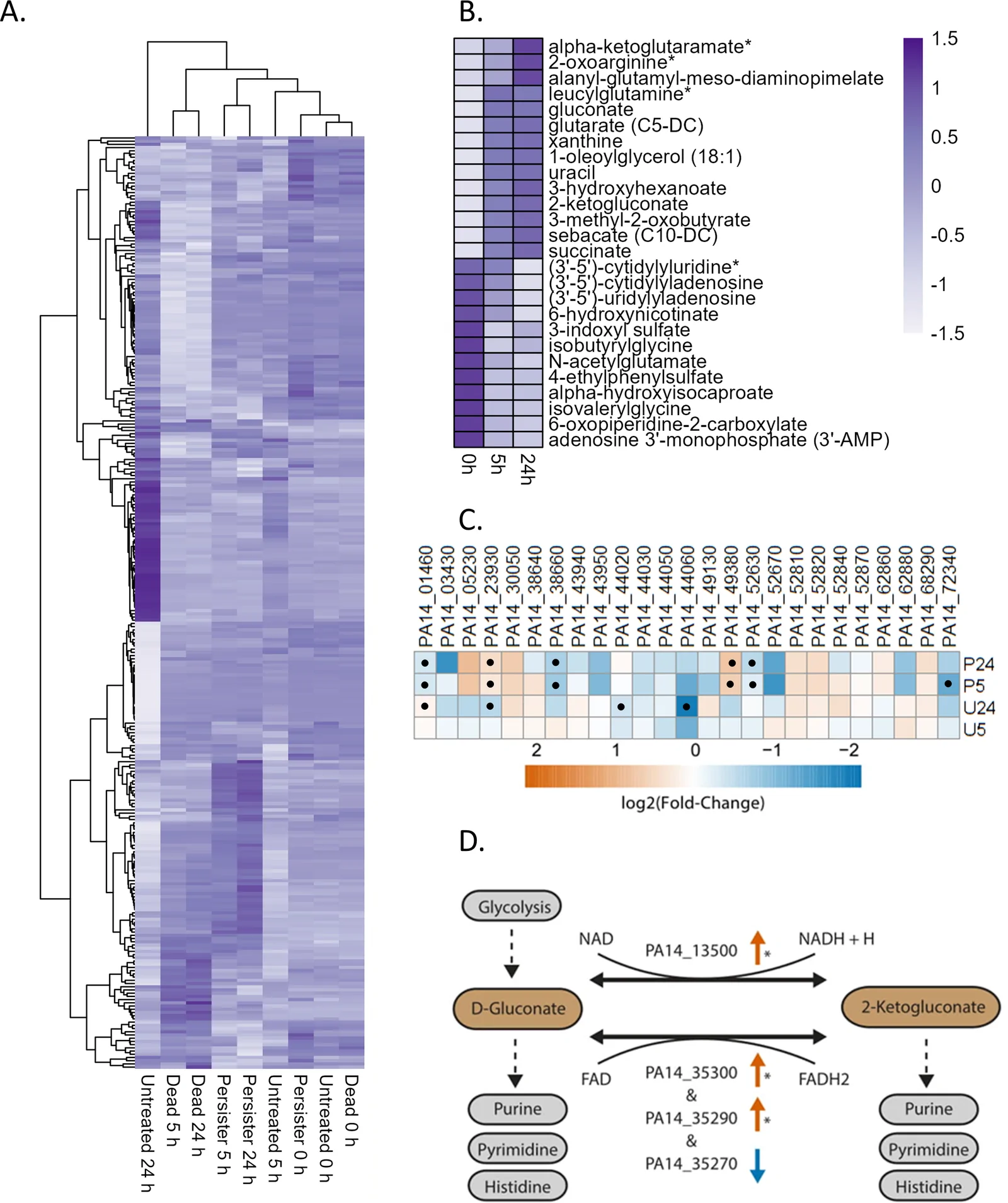
Metabolomics profiling of culture supernatants. Samples were collected for metabolomics profiling from the culture supernatants of the untreated, persister, and dead conditions (0, 10×, and 1,000× the MIC, respectively) immediately before exposure and 5 and 24 h after exposure. (**A**) Metabolites that significantly changed in abundance over time for each condition were identified. The heatmap shows the abundance for a metabolite in a given sample relative to the mean abundance for that metabolite across all samples. (**B**) Metabolites that changed in abundance over time only in the persister condition were identified. The heatmap shows the abundance for a metabolite at a given timepoint relative to the mean abundance of that metabolite across all persister timepoints. (**C**) Heatmap of the log_2_(fold-change) for genes involved in succinate metabolism. Dots denote an adjusted *P*-value ≤ 0.05. (**D**) Metabolism of d-Gluconate and 2-Ketogluconate. Brown boxes refer to metabolites, while gray boxes refer to other metabolic pathways. Orange arrows indicate upregulation in persister conditions relative to time 0 samples. Blue arrows indicate downregulation in persister conditions relative to time 0 samples. Asterisks denote significant differential expression (*P*_adj_ ≤ 0.01).

To probe the metabolism of *P. aeruginosa* persister cells further, culture supernatants were collected for metabolomics profiling from the persister and untreated conditions immediately before exposure and 5 and 24 h after exposure (same cultures used for RNA samples). Additionally, samples from the 1,000× MIC condition were collected (henceforth referred to as the dead condition) to filter metabolites associated with cell death due to BIT treatment. Analysis of the relative abundance of metabolites across the samples revealed that the persister metabolite footprint is distinct from the untreated and dead conditions (Fig. 4A). The two BIT-exposed persister conditions clustered separately from the other conditions. Furthermore, certain metabolites were markedly more abundant in the persister samples relative to the other samples. For example, 20 metabolites were significantly more abundant in the persister condition at both 5 and 24 h after exposure compared to the other conditions at those respective time points.

In contrast, 60 metabolites in culture supernatants significantly changed in abundance over time for every condition (Fig. S5). Interestingly, it varied whether these 60 metabolites were produced or consumed across the conditions. For example, while ribose was depleted for each condition, arginine was only depleted for the untreated condition but increased in abundance for the persister and dead conditions. The accumulation of arginine in the persister and dead samples is likely due to the prevalence of dying cells in both conditions. Other metabolites varied in abundance trajectories across all three conditions. For example, N6-acetyllysine accumulated rapidly for the dead condition, accumulated slowly for the untreated condition, and was depleted in the persister condition. Lysine acetylation is associated with cell-cell signaling, transcriptional regulation, and cell survival pathways in mammalian systems (37). Mounting evidence suggests that lysine acetylation in prokaryotes plays a similar role (37, 38). Thus, this difference in N6-acetyllysine dynamics between the persister and untreated and dead conditions may indicate a difference in regulatory activities.

Additionally, we identified metabolites that uniquely varied across time for a given condition (Fig. 4B; Fig. S4). For example, succinate accumulated over time for the persister condition but not for the untreated and dead conditions. Filtering the data this way allowed us to determine metabolites uniquely associated with the untreated (*n* = 53), dead (*n* = 35), and persister (*n* = 26) conditions. The reduced number of time-varying metabolites in persister cells may reflect limited exchange with the extracellular environment (39), resulting in fewer metabolites that accumulate or deplete over time. In contrast, untreated cells undergo active growth-associated uptake and secretion, while dead cells experience loss of membrane integrity and passive metabolite release, both of which promote broader temporal metabolic variation.

Transcriptomics analysis revealed that genes associated with the production and consumption of unique persister metabolites were differentially regulated. For example, the metabolomics data suggested that succinate production is uniquely associated with the persister phenotype. The RNA-sequencing data also indicated a difference in succinate metabolism, with unique differential regulation of succinate-associated genes across the conditions (Fig. 4C). Succinate is a key intermediate in the tricarboxylic acid (TCA) cycle. Previous studies have implicated the TCA cycle in persister cell formation and viability. Indeed, one study showed that accumulation of fumarate, the product of succinate oxidation, enhanced persister formation (40). Other studies showed that mutants of TCA genes, such as *sdh* and *sucB*, significantly reduced persister levels (13, 40, 41). The role of succinate appears highly dependent on experimental context. While introducing exogenous succinate to nutrient-exhausted cells can jumpstart cellular metabolism and successfully potentiate aminoglycoside killing (42), our study investigates a distinct phenomenon: the active accumulation and extracellular excretion of endogenous succinate following BIT treatment. Rather than acting as a metabolic potentiator in this context, the overproduction and efflux of succinate observed here likely reflect a downstream metabolic reallocation or overflow pathway characteristic of the stress-induced persister state, making it a valuable extracellular biomarker for this specific phenotype. Indeed, the previous study showed that carbon-source induced potentiation was specific to tobramycin and not applicable to other antibiotics (42). Additionally, another study found that inhibitors of the TCA cycle enhanced persister formation in gram-positive bacteria (12). Altogether, these results indicate that the TCA cycle plays an important, albeit heterogeneous, role in persister viability depending on the stressor.

In a second example, both the metabolomics data and transcriptomics data indicated enhanced gluconate metabolism in persister conditions (Fig. 4D). Additionally, genes associated with the conversion of gluconate and 2-ketogluconate were significantly increased in the persister samples but not the untreated samples. Both gluconate and 2-ketogluconate are important precursors of purine and pyrimidine metabolism; however, the role of these two metabolites is understudied. Interestingly, previous research implicated gluconate accumulation with multidrug-resistant *P. aeruginosa* clinical isolates (43). Together with our results, gluconate accumulation appears to be associated with antimicrobial inefficacy and may serve as a good biomarker for tolerant subpopulations. Future work will be necessary to determine whether targeting gluconate metabolism is a potential antimicrobial strategy.

### Condition-specific modeling of the persister and untreated states

Finally, to aid in the interpretation of the transcriptomics data set, we generated condition-specific metabolic models of the persister and untreated metabolic states. For our analyses, we explored two objective functions: biomass synthesis and ATP production. Because persister cells are clearly not attempting to maximize biomass synthesis, the choice of this objective function is to explore the requirements for cell growth and not an indication that the network itself is actively maximizing growth. We also investigated ATP production as an objective function, which has previously been shown to be associated with persistence (16). Additional objective functions to explore metabolic network functional capabilities could also be explored.

To build the models, we integrated the transcriptomics data set with a previously published GENRE of *Pseudomonas aeruginosa* strain PA14 (26). We integrated the RNA-sequencing data set with the GENRE using the Reaction Inclusion by Parsimony and Transcript Distribution (RIPTiDe) algorithm (44), which uses both transcriptomic abundances and parsimony of overall flux to identify the most efficient metabolic network activity in the cell. Each transcriptomic data replicate was used to create a different condition-specific model to investigate any heterogeneity between replicates. This process was repeated with both biomass and ATP as the objective functions. This process was used to create condition-specific models for the metabolism of the persister and untreated states at 0, 5, and 24 h after exposure to BIT. The resulting models differed in their predicted functionality. Flux sampling was performed on the context-specific models, and PCA was applied to reduce the dimensions of the resulting flux distributions to facilitate visualization (Fig. S6). Although there is an overlap between the treated and untreated metabolism, suggesting a common feasible metabolic state, there is a distinct cluster for the treated cells at 5 and 24 h, demonstrating that the treated cells can potentially adopt a distinct metabolic state. The models generated with ATP production as an objective function showed a different clustering pattern. Many of the persister models at 24 h still clustered together tightly, while the untreated models at 24 h demonstrated more dispersion. Additionally, some of the flux samples at 5 h, both treated and untreated, had high positive PC1 scores. The reactions with the highest loadings in PC1 for the ATP models were adenosine transport and adenosine kinase (ADK). These reactions help maintain the overall balance of adenine nucleotides, which are crucial for energy homeostasis and ATP supply, especially in times of stress (45). It is possible that some of the untreated 5-h models require additional ATP due to the energy requirements for exponential growth, while the persister models at 5 h may be using the ATP to tolerate the antimicrobial through mechanisms such as efflux pumps and ATP-dependent dynamic protein aggregation (46). Although the persister models optimized for biomass production clustered tighter and further away from the other biomass-optimized models, the persister models optimized for ATP production showed a difference when plotted in this reduced-dimensional space. Overall, this result demonstrated that the persister condition results in different flux distributions through the models, indicative of a different metabolic signature from the untreated conditions.

Using these condition-specific metabolic models, we sought to identify genes that, when inhibited, interrupted persister cell viability. We implemented three different strategies for both objective functions to identify these reactions. In the first strategy, we performed *in silico* single-reaction knockouts on the models and identified reactions predicted to be essential in persister models, but non-essential for the untreated models. In the second strategy, we ran a Mann–Whitney test to determine the reactions that were most significantly different between the persister state and the untreated state at 24 h. In the third strategy, we used the flux sampling data to train a random forest classifier to determine the reactions that were most predictive in differentiating between the persister and untreated models. The flux sampling data from models optimized for biomass synthesis achieved 93% accuracy in differentiating between persister and untreated, while the flux sampling data from the models optimized for ATP production only achieved an 85% accuracy. This is likely because biomass is a more complicated objective function, allowing for more varied pathways to produce it, while ATP is more restrictive in the metabolic pathways that lead to it. To arrive at a set of genes for experimental testing, we filtered this list of candidate target genes based on several factors, such as gene-product pathway and availability of transposon mutants in the PA14 mutant library (47). We made sure not to test mutants for genes that had the same product or were involved in the same reactions. The three strategies used yielded lists of potential targets with significant overlap, so many of the targets selected were identified by more than one of our strategies.

**Fig 5:**
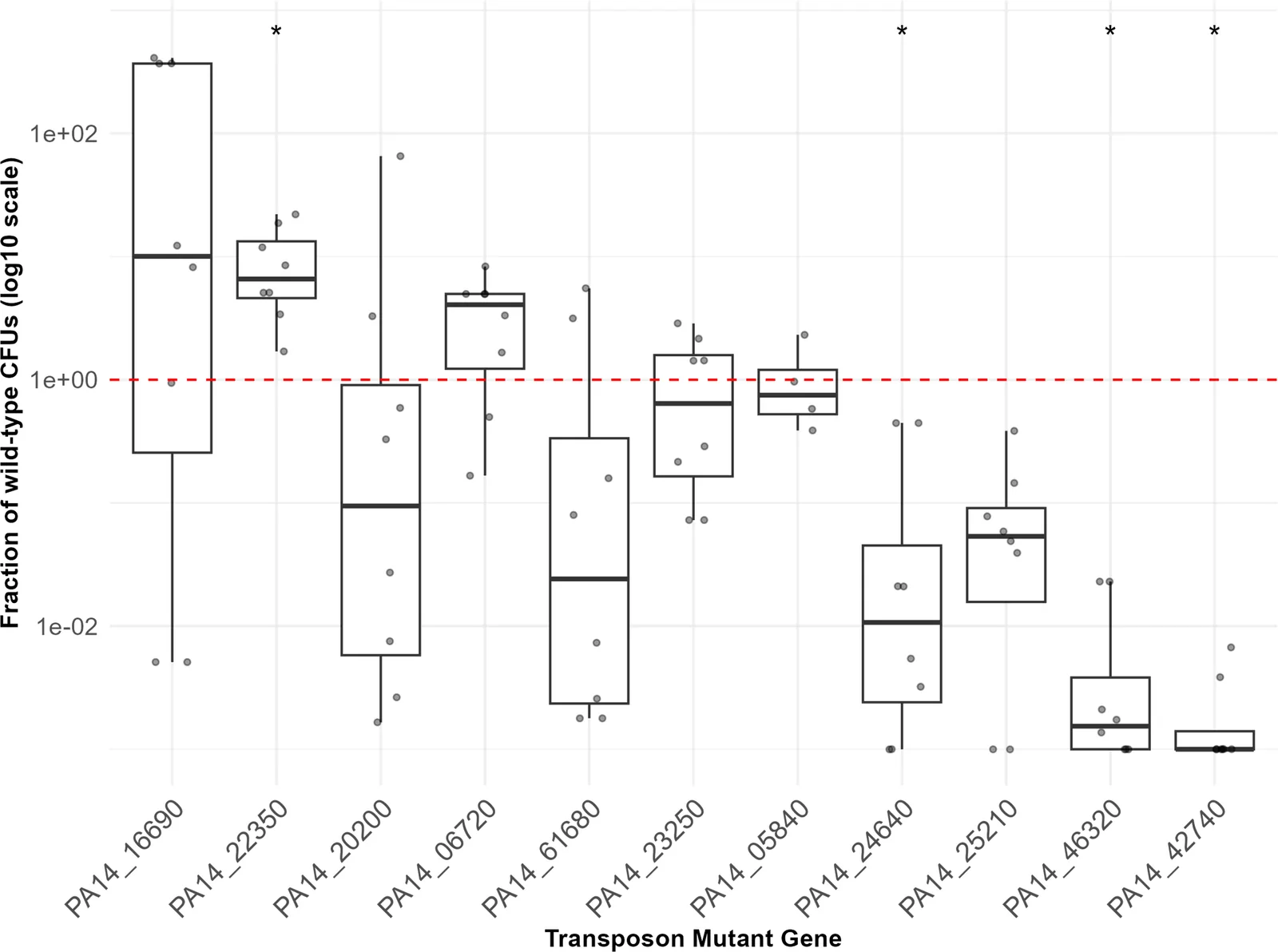
Time-kill assays for *P. aeruginosa* mutants. Mutants of genes predicted to be critical for persister viability were exposed to 10× the MIC of BIT (0.02% BIT) for 24 h. The number of persister CFUs in the culture at 24 h after BIT exposure was normalized to the wild-type PA14 by dividing the OD_600_ on the growth curve for each mutant at 24 h (Fig. S7) by that of the wild type at 24 h. This allows the resulting persister CFU counts to be compared irrespective of growth differences. At 24 h, asterisks denote significant difference in normalized CFU count compared to the ancestor (Wilcoxon test with BH correction *P*_adj_ ≤ 0.05).

Ultimately, we experimentally tested 11 PA14 single-gene mutants that were predicted to be critical for the biomass and ATP objective functions analyzed. Selected mutants and an overview of each selected gene can be found in Table S4. Following the same experimental procedure as before, we exposed the mutants to 10× the MIC of BIT and performed a time-kill assay (Fig. 5). Of the 11 mutants tested, 3 exhibited a significant reduction in the number of CFUs after 24 h of BIT exposure (*P*_adj_ ≤0.05).

A mutation in PA14_42740 significantly decreased persister formation, and a mutation in PA14_25210 caused a noticeable decrease in persister formation although not statistically significant. PA14_42740 encodes for a putative sugar aldolase, while PA14_25210 encodes for 5′-methylthioadenosine phosphorylase (MTAP), both of which are involved in the methionine salvage pathway. The methionine salvage pathway plays a crucial role in sulfur recycling and maintaining intracellular methionine pools (48). Methionine metabolism underpins key cellular processes, including methylation reactions, polyamine synthesis, and defense against oxidative stress, thereby helping persister cells withstand reactive oxygen species generated by antimicrobial treatments. Previous work has demonstrated that methionine availability can influence the formation of bacterial persisters by adding exogenous methionine, which decreased the formation of persisters (49). This indicates that methionine import might be an important driver of persister cell formation. The methionine salvage pathway allows for a low-cost method of recycling a byproduct of polyamine synthesis back into methionine, rather than using ATP for an ABC transporter. Targeting this pathway may cause the cell to adopt a more metabolically active phenotype to synthesize or import methionine, thus leading to a decrease in persisters.

The PA14_46320 mutation also decreased the formation of persister cells. PA14_46320 encodes for pyruvate carboxylase, which catalyzes the carboxylation of pyruvate to oxaloacetate. This reaction is important for replenishing the TCA cycle. While the TCA cycle inactivation has been linked to persister cell formation via ATP depletion (50), other bacteria have been shown to exhibit increased antimicrobial susceptibility when pyruvate carboxylase was disrupted (51). Pyruvate carboxylase disruption may actually decrease persister formation due to impaired metabolic flexibility through the TCA cycle, which would allow the cell to sustain an energy-efficient metabolism for adopting the persister state.

The final mutation that significantly decreased persister cell formation was PA14_24640 (*pyrD*), which encodes for dihydroorotate dehydrogenase 2, an enzyme involved in *de novo* pyrimidine biosynthesis. *pyrD* mutants have been shown to display defects in cytotoxicity, biofilm formation, and virulence in an acute mouse pneumonia model (52). Persister cells still require small amounts of DNA and RNA synthesis for repair and maintenance, especially in the presence of an antimicrobial that can result in oxidative stress, like BIT.

Eight of the eleven mutants yielded no significant decrease in persister cell formation. This is plausible because RIPTiDe is a parsimonious algorithm, producing the simplest model that sends flux through the highly expressed reactions. Some of the genes that were identified as potential targets have alternate pathways to reach the same products. For example, PA14_05840 encodes for glutaryl-CoA dehydrogenase, an enzyme involved in lysine and tryptophan metabolism. Catabolism of these substrates is often condition-specific and able to be bypassed. The energy and redox needs could be met from other carbons, so persister physiology may be unchanged (53). This redundancy in *P. aeruginosa* metabolism allows the bacterium to maintain critical functions despite single-gene disruptions, explaining the lack of impact on persister formation for these mutants. For targeting more complex reactions and processes that contain redundancies, multiple gene knockouts might need to be performed.

## DISCUSSION

Traditionally, persister cells are thought to be able to tolerate antimicrobial treatment due to a reduced metabolic state. However, the extent of persister cell metabolism is not well understood, especially in the presence of biocidal disinfection. In this study, we employed both experimental and computational systems biology techniques to better characterize the metabolism of biocide persister cells. We applied this approach to persister cells isolated from *P. aeruginosa* cultures exposed to the industrial antimicrobial benzisothiazolinone. We collected both transcriptomics and metabolomics data on persister cell samples, which indicated that biocide persister cells are in a distinct metabolic state relative to untreated samples, marked by increased activity in central metabolism. We then integrated the transcriptomics and metabolomics data sets with a genome-scale metabolic network reconstruction of *P. aeruginosa* to generate a condition-specific model of persister cell metabolism. Using this model and information from the experimental data, we investigated key differences in metabolic reaction essentiality and flux distribution. Ultimately, this experimental and computational approach enabled the characterization of persister cell metabolic activity and the elucidation of persister cell metabolic processes predicted to be essential that could be targets to interrupt persister cell functionality. A growing body of work supports the idea that persister cells maintain a controlled, low-flux but active metabolic state rather than complete dormancy. Both the transcriptomics and metabolomics data sets from this study indicate that biocide persister cells are not in a dormant state. Rather, these two data sets suggest that these persister cells are active in central metabolic processes. These findings align with previous studies demonstrating that persister cells often maintain or even increase transcription of metabolic genes relative to stationary-phase cells, reflecting a shift toward stress-adapted metabolism rather than shutdown (54). Central metabolic pathways, such as the TCA cycle, electron transport chain, and ATP synthase, have been shown to be crucial for sustaining persister levels within cell populations (55). Additional work has shown that persisters maintain basal proton motive force and redox balance to survive under these lethal stress conditions (56, 57). Together with our findings, this growing literature supports the view that the persister state is metabolically active rather than globally repressed (11, 13, 58, 59). However, because no single pathway was uniquely associated with the persister state, this result points to the heterogeneity and complexity of the persister phenotype.

The apparent variability in the percentage of genes in Fig. 3B, particularly in the untreated groups, could be interpreted as reflecting the natural variation in gene expression rather than biologically meaningful pathway-level changes. It is important to note that each experimental condition in this study was supported by eight independent RNA-seq replicates, and replicate-level variance was explicitly incorporated into differential expression testing using DESeq2. Adjusted *P-*values for each gene were therefore calculated based on both effect size and dispersion across replicates, and only genes meeting a stringent significance threshold (adjusted *P* ≤ 0.01) were included in subsequent gene set enrichment analyses. The higher apparent variance in the percentage of genes assigned to enriched pathways in untreated samples is primarily attributable to the substantially smaller number of differentially expressed genes detected under these conditions relative to the persister samples. When the absolute number of differentially expressed genes is low, small changes in gene membership can result in proportionally larger fluctuations in pathway representation, which reduces statistical power and increases apparent variability. Although this may contribute to minor variability in enrichment outcomes for the untreated groups, it does not undermine the robustness or biological relevance of the metabolic pathways consistently enriched in the persister conditions, where a much larger and more statistically powerful set of differentially expressed genes was observed.

The results from our computational modeling identified key reactions that could be targeted to decrease the number of persister cells that arise from antimicrobial treatment. We found that the PA14_24640 (*pyrD*) mutant displayed a significant decrease in persister formation. This is plausible because it has been demonstrated that *P. aeruginosa pyrD* mutants experience defects in cytotoxicity, biofilm formation, quorum-sensing, and virulence in an acute mouse pneumonia model (52). The inhibition of these mechanisms could be linked to an inability to form stable persister cells, especially since persister cells are enriched in biofilms (60). It is interesting that our PA14_06720 (*nirF*) mutant also did not experience a decrease in persister cell formation because *nirF* is required for the biosynthesis of heme d1 of nitrite reductase. *P. aeruginosa* ATCC 9027 strains with nitrate reductase (*nirS*) mutations experienced a decrease in biofilm formation and swimming motility, but not in planktonic growth, relative to wild type when exposed to BIT (61). Since our experiments evaluated planktonic cells in shaking liquid cultures, biofilm formation was not a prerequisite for persistence. This suggests that *pyrD* mutations impair persistence through an alternative pathway independent of biofilm production. Further work will be needed to better understand how *pyrD*-associated metabolic processes influence persister cell biology.

Strain-level differences in metabolism and gene regulation are well documented across bacterial species, and these distinctions may influence the persister behavior we observed in PA14 (62). Comparative genomics and multi-omics studies have demonstrated that closely related strains can vary in biosynthetic pathways such as methionine uptake and synthesis (63, 64). Moreover, a collection of genome-scale metabolic reconstructions from 55 *E. coli* and related strains showed that some strains completely lacked methionine synthesis pathways, demonstrating that metabolic potential can be strain-specific (64). Additionally, strain-specific metabolism has been linked with genomic variants in specific metabolic genes and differential expression of those corresponding genes (65). Taken together, it is possible that distinct *P. aeruginosa* strains, both clinical and environmental, may exhibit differential persister metabolism. Given these metabolic differences between bacterial strains, our choice to use a single *P. aeruginosa* strain represents a limitation in the present work. While PA14 is widely used because of its virulence, its regulatory and metabolic characteristics may not generalize to the entirety of the species. Future studies incorporating multiple strains will be necessary to determine whether the pyrimidine and methionine pathways represent viable targets for persister cell inhibition. Additionally, we did not perform genetic mutational analysis on persister cells to identify any genetic changes that may occur in the development of the persister phenotype. Although some studies show that persistence may be linked to mutation rates (66), persistence is classically defined as a form of tolerance that is not acquired through heritable mutations, but rather through phenotypic differentiation (67). Although mutational analysis can be valuable for understanding long-term evolutionary adaptation, it is not typically expected to explain the transient, reversible formation of persisters. Nonetheless, integrating variant analysis and functional protein-level assessment in future studies, particularly across multiple strains, may help determine whether specific genomic backgrounds predispose cells to distinct persister metabolic states.

Methionine metabolism plays an important role in shaping *P. aeruginosa* under stress, and recent studies have demonstrated that amino acid metabolism is tightly coupled to quorum-sensing and virulence regulation (68–70). Although our enrichment analysis did not show any significant change in the quorum-sensing gene sets, the *pqsABCDE* operon was upregulated in the persister cells at 24 h, and *lasR* was downregulated in the BIT-treated group at 5 h. These observations suggest that while canonical *las-* and *rhl*-mediated quorum-sensing signaling is not sustained during the persister state, components of the PQS branch may still be responsive to antibiotic stress or metabolic shifts. However, the transient nature of *lasR* repression and the lack of global quorum-sensing pathway enrichment indicate that quorum-sensing modulation is unlikely to be the primary driver of persister formation in this system. Instead, the induction of *pqsABCDE* may reflect a stress-associated or metabolic signaling role rather than a coordinated quorum-sensing-driven regulatory program. Nevertheless, because quorum-sensing systems broadly regulate stress-responsive gene expression and coordinate adaptive behaviors, these preliminary signals of *pqs* activation highlight an important avenue for future work aimed at understanding how quorum-sensing pathways may interact with metabolic pathways during persister formation.

While our transcriptomic and metabolomic analysis identified several key metabolic genes whose disruption significantly alters *P. aeruginosa* persistence during BIT exposure, we acknowledge that formal genetic complementation of these loci was not performed in this study. Consequently, the potential for secondary background mutations or minor polar effects on downstream operon architecture cannot be entirely ruled out. Nonetheless, the robust reproducibility of the persistence phenotypes across all independent biological replicates supports the conclusion that these metabolic pathways are fundamentally involved in modulating *P. aeruginosa* persistence. These genes represent high-priority targets for future targeted biochemical complementation and detailed mechanistic characterization to dissect exactly how they affect antibiotic tolerance.

BIT is widely used as a biocide in industrial products, including paints and detergents, where its concentration can vary substantially. Reported concentrations in United States paint products range from 29 to 1,111 ppm, and in European paints from 0.1 to 462 ppm, with typical formulations containing approximately 200–400 ppm, equivalent to 0.02%–0.04% BIT (71, 72). In this study, the 10× MIC BIT exposure corresponds to ~ 0.02%, aligning with concentrations commonly present in commercial products. The reported toxicity of BIT to aquatic organisms and potential adverse effects in mammals, including contributions to blood–brain barrier dysfunction and cardiovascular or neurological risks (73, 74), highlight the importance of understanding the bacterial response to this biocide. Our data indicate that even at concentrations within typical commercial ranges (~0.02% BIT), *P. aeruginosa* can tolerate BIT treatment via a subpopulation of persister cells, underscoring the need to consider broader environmental and health impacts when using BIT-containing products. Improved knowledge of persister biology may ultimately reduce the need for environmentally and physiologically harmful compounds in industrial and clinical settings.

We recognize that the biomass harvested for RNA sequencing represents an operational definition of persister cells. Operationally defining persisters by their capacity to withstand supra-MIC concentrations of an antimicrobial is a standard method for kill-curve generation (29). For bulk omics, however, this approach means the analyzed biomass represents the collective signature of all cells that have structurally resisted lysis and have intact RNA at the time of collection. This likely encompasses a mix of true, dormant persisters and non-viable but intact cells. As stated before, any residual mRNA within dead or structurally compromised cells would undergo progressive degradation over the 24-h BIT treatment period. Additionally, persistence is a transient phenotypic state, and other methods of isolation for transcriptomic analyses can introduce handling artifacts, trigger a mechanical stress response, or initiate a resuscitation of the persister phenotype (75). The fact that we resolve a robust, highly coordinated, and distinct transcriptional profile at both 5 h and 24 h, rather than a random or completely degraded transcript distribution, strongly indicates that the sequenced RNA reflects an actively maintained biological program. This active signature is further evidenced by the predictions of gene targets that inhibit the development of the persister cell phenotype.

In summary, biocide persister cell metabolism is distinct from untreated cells and may be more metabolically active than previously appreciated. Characterization of the biocide persister metabolic state enabled the identification of processes unique to the persister phenotype that may be crucial to persister cell functionality. Further work needs to be performed to determine whether these attributes are common to persister cells generated by other stressors as well as persister cells from other bacterial species. Finally, computational modeling of persister cell metabolism enabled the identification and validation of key reactions that are essential for the development of persister cells.

## MATERIALS AND METHODS

### Bacterial strains and growth conditions

Wild-type *Pseudomonas aeruginosa* PA14 was grown in LB media at 37°C and 125 RPM for liquid cultures. Desired concentrations of BIT (Lonza Proxel GXL) were made by diluting the antimicrobial in PBS. The Lonza Proxel GXL was passed through a 0.22 µm filter prior to use. We used LB media for this study because nutrient-rich conditions promote the high-density stationary-phase physiology from which persisters naturally arise (28).

### Minimum inhibitory concentration assay

This assay was performed via the broth microdilution method (27). A frozen stock of PA14 was streaked on an LB agar plate and grown overnight. Individual colonies were inoculated into 10 mL of LB and grown overnight with aeration. The culture was then diluted to an OD_600_ of 0.001 (approximately 10^6^ CFU/mL) and inoculated into a 96-well plate containing varying concentrations of BIT. The plate was incubated overnight in a plastic container to prevent evaporation at 37°C and 125 RPM. Approximately 16 h after exposure, the OD_600_ was measured using a plate reader (Tecan Infinite M200 Pro) as an approximation for growth. Background subtraction was performed, and the minimum inhibitory concentration was defined as the lowest concentration that prevented growth of PA14 (i.e., OD_600_ ≤ 0.1).

### Persister isolation for time-kill assay and re-growth

The persister isolation assay was performed similarly to previously published methods (30). A frozen stock of PA14 wild type or mutants was streaked on an LB agar plate and grown overnight. Individual colonies were inoculated into a 50 mL flask containing 10 mL of LB and grown overnight with aeration. The overnight cultures were diluted in fresh LB in 500 mL flasks to an OD_600_ of 0.01 for a total volume of 50 mL and grown for 12 h to reach the stationary phase. The cultures were then subsequently exposed to BIT by removing 5 mL of the culture and adding 5 mL of antimicrobial at various concentrations (PBS for untreated, 0.02% BIT for 10× MIC, 0.2% BIT for 100× MIC, and 2% BIT for 1,000× MIC). The flasks were returned to the incubator for 24 h. Milliliter samples were collected to determine the number of viable cells in the culture immediately before exposure (0 h), 2.5 h, 5 h, and 24 h after exposure. Samples were washed in PBS twice, followed by resuspension in 1 mL of PBS. The samples were then serially diluted in PBS, and 10 µL of culture was plated for each dilution on LB agar to measure the number of CFUs. CFU counts were plotted against time to generate the time-kill curve. For the time-kill curve, a Kruskal–Wallis test with Dunn’s post hoc test was used to determine differences between CFUs in the curves (Table S3).

To verify the re-growth of the persister cells, 1 mL samples were collected from the untreated and 10× MIC conditions. Samples were washed in PBS twice and resuspended in 1 mL PBS. The 10× MIC condition was diluted 1:100, and the untreated condition was diluted to approximately the same CFU concentration as the diluted 10× MIC condition (~10^3^ CFU/mL). The diluted cultures were plated in a 96-well plate, and the OD_600_ was measured using a plate reader (Tecan Infinite M200 Pro) for 24 h.

### Live/dead staining and imaging

To prepare for staining, 1 mL of each culture was washed by centrifuging at 5,000 × *g* for 5 min, discarding supernatant, and resuspending with 1 mL of 0.9% sodium chloride. This was repeated three times before a final resuspension in 1 mL 0.9% sodium chloride. Subsequently, treated samples were further diluted 1:2 and untreated samples 1:50 to a total volume of 1 mL. Equal volumes of the two components of the Live/Dead BacLight stain were mixed, and 3 µL was added to each sample. After incubation at room temperature for 30 min, each sample was concentrated onto black polycarbonate track-etched membranes using a Swinny filter holder. Membranes were imaged at 40× and 100× with a Keyence BZ-X800 using the GFP and TRITC filter cubes and processed using CellProfiler (76). For each condition (persister 24 h and untreated 24 h), we imaged three biological replicates, taking five fields of view for each replicate

### RNA sequencing

One-milliliter samples for RNA sequencing were collected immediately before exposure, 5 h and 24 h after exposure from the untreated and 10× MIC conditions. We did not isolate RNA from the dead conditions (1,000× MIC) as we did for the metabolomics analysis because RNA quality was extremely poor for this condition. Each condition included eight biological replicates. Samples were mixed with 2 mL of RNAprotect (Qiagen) to stabilize RNA and frozen at −80°C until RNA was isolated using the RNeasy kit (Qiagen). Samples were sent to Genewiz for further processing. Ribosomal RNA was depleted using the Ribo-Zero rRNA removal kit (Illumina). Libraries were sequenced on an Illumina HiSeq, 2 × 150 bp configuration. RNA concentrations and quality scores are provided in File S2.

Reads were aligned to the reference *P. aeruginosa* PA14 genome (NC_008463.1) using the BWA aligner with the MEM algorithm with default parameters (77). Aligned reads were tallied for each gene using FeatureCounts (78). Differential expression was then determined with DESeq2 (79). DESeq2 results can be found in Table S1. Principal component analysis (PCA) of variance-stabilized RNA-seq counts for each sample, with batch effects accounted for, can be seen in Fig. S8. Genes with an adjusted *P*-value ≤ 0.01 were deemed differentially expressed. DEGs were inspected, and functional class enrichment was performed using the provided “term_erich.py” Python script using gene-term associations present in KEGG classification databases obtained from Pseudomonas.com (80). Classes with a *P*-value (Hyper-geometric test) and adjusted *P*-value (Bonferroni correction for multiple tests) ≤0.01 were considered statistically significant. The code used to perform the enrichment analysis can be found in the project GitHub. Raw data from the RNA-Seq analysis gene expression are available on the GEO repository (https://www.ncbi.nlm.nih.gov/geo/) with the accession number GSE185914.

### Metabolomics profiling

Six-milliliter samples for supernatant metabolomics profiling were collected immediately before exposure, 5 and 24 h after exposure from the untreated, 10× MIC, and 1,000× MIC conditions. Each condition included eight biological replicates. To prevent media variability from confounding our biological findings, we utilized a balanced block design: each experimental group contained four replicates within each of the two batches. The samples were centrifuged for 5 min at 6,000 RCF. The resulting supernatant was filter sterilized (0.2 µ filter) in 1 mL aliquots and frozen at −20°C. The samples were shipped to Metabolon (Durham, NC) for metabolomics data collection. Briefly, all methods utilized a Waters ACQUITY ultra-performance liquid chromatography (UPLC) and a Thermo Scientific Q-Exactive high resolution/accurate mass spectrometer interfaced with a heated electrospray ionization (HESI-II) source, and an Orbitrap mass analyzer was operated at 35,000 mass resolution. Sample extract was dried and reconstituted in solvents compatible with each of the four methods described below. One aliquot was analyzed using acidic positive ion conditions optimized for hydrophilic compounds with a C18 column (Waters UPLC BEH C18−2.1 × 100 mm, 1.7 µm) using water and methanol, containing 0.05% perfluoropentanoic acid (PFPA) and 0.1% formic acid (FA). Another aliquot was analyzed using acidic positive ion conditions optimized for hydrophobic compounds with the same C18 column using methanol, acetonitrile, water, 0.05% PFPA, and 0.01% FA, and was operated at an overall higher organic content. Another aliquot was analyzed using basic negative ion optimized conditions with a separate dedicated C18 column. The basic extracts were gradient eluted from the column using methanol and water with 6.5 mM ammonium bicarbonate at pH 8. The fourth aliquot was analyzed via negative ionization following elution from a HILIC column (Waters UPLC BEH Amide 2.1 × 150 mm, 1.7 µm) using a gradient consisting of water and acetonitrile with 10 mM ammonium formate at pH 10.8. The MS analysis alternated between MS and data-dependent MS^n^ scans using dynamic exclusion. The scan range varied slightly between methods but covered 70–1,000 m/z. Library matches for each compound were checked for each sample and corrected if necessary. Peaks were quantified using the area under the curve. Missing values were imputed with half the minimum. Metabolomics data from Metabolon can be found in Table S2. Data were log_2_-transformed and centered around the mean. Following normalization of the metabolomics data, PCA was used to ensure that samples clustered by treatment condition rather than batch (Fig. S9). Metabolites significantly changing over time were identified using a Welch one-way ANOVA with a Games–Howell post hoc test (*P* ≤ 0.05). Metabolites significantly changing across the samples were identified using a one-way ANOVA with a Tukey post hoc test (*P* ≤ 0.05). A description of Metabolon’s internal processing of the metabolomics data can be found in File S1. (Note: The concentrations reported in the Metabolon report are the concentration of Proxel GXL, which is 20% BIT.)

### Condition-specific model generation

Transcriptomics data were integrated with the PA14 genome-scale metabolic network reconstruction (26) using the algorithm RIPTiDe (44) implemented with COBRApy (81). Briefly, RIPTiDe assumes a global minimization of flux (similar to other established methods like pFBA) while maximizing the inclusion of reactions corresponding to highly transcribed genes. Thus, RIPTiDe identifies the parsimonious pathways that best satisfy the objective function of the model while maximizing agreement with gene expression data. We chose to investigate biomass production because it is a standard, well-studied objective function in the field of metabolic modeling. However, because persister cells are traditionally thought to be in a dormant metabolic state, biomass production may not be the best-suited objective function. To complement our analysis, we also investigated ATP production, which has previously been shown to be associated with persistence (16). Two condition-specific models were created for each biological replicate sent for RNA sequencing, one with biomass as the objective, and one with ATP maintenance.

### Model prediction of persister targets

Three strategies were implemented to identify potential gene targets. In the first, we performed *in silico* single-gene knockouts on the condition-specific models and identified reactions essential for minimal flux through the objective function (threshold set at 0.0001 h^−1^). We repeated this simulation for models with ATP and those with the biomass objective. We then identified the genes that were the most essential in the persister models at 24 h and nonessential in the untreated models at 24 h.

In the second strategy, we used RIPTiDe with the built-in flux sampler, Gapsplit (82), to perform flux sampling on the condition-specific models. We ran a Mann–Whitney test to determine the reactions that were most significantly different between the persister state and the untreated state at 24 h.

In the third strategy, we used the flux sampling data to train a random forest classifier to distinguish between the untreated and treated samples at 5 and 24 h. We then mapped importance values to each reaction and selected the reactions that had the highest importance in both the biomass and ATP models.

## Data Availability

All data, code, and computational workflows associated with this study are made available in accordance with the FAIR (Findable, Accessible, Interoperable, and Reusable) principles. All data processing, modeling, and analysis scripts are publicly available through a version-controlled GitHub repository and archived on Zenodo (GitHub: https://github.com/csbl/Persister_project; Zenodo DOI: doi.org/10.5281/zenodo.17992667), ensuring long-term accessibility and citation. RNA-seq data generated and analyzed in this study have been deposited in the NCBI Gene Expression Omnibus under accession number GSE185914. Software dependencies and computational environments are explicitly defined using renv for R and pyproject.toml/Poetry for Python, enabling exact environment restoration and full computational reproducibility.doi.org

## SUPPLEMENTAL MATERIAL

File S1 jb.00373-25-s0001.pdf Metabolon report.

File S2 jb.00373-25-s0002.pdf Genewiz RNA quality and quantity.

Supplemental figures jb.00373-25-s0003.pdf Figures S1 to S9.

Table S1 jb.00373-25-s0004.xlsx The DESeq2 comparisons between experimental conditions.

Table S2 jb.00373-25-s0005.xlsx The processed metabolomics data from Metabolon.

Table S3 jb.00373-25-s0006.xlsx The statistics for comparing the time-kill curves.

Table S4 jb.00373-25-s0007.xlsx The gene descriptions for the PA14 single-gene mutants used in the mutant-validation experiment, including UniprotKB IDs.

ASM does not own the copyrights to Supplemental Material that may be linked to, or accessed through, an article. The authors have granted ASM a non-exclusive, world-wide license to publish the Supplemental Material files. Please contact the corresponding author directly for reuse.
