# Supplementary material for: Integrating multi-omics data with network modeling to characterize Pseudomonas aeruginosa persister cell metabolism
Source: J Bacteriol. 2026 Jul 20;208(8):e00373-25. doi: 10.1128/jb.00373-25 (PMC13492110; doi:10.1128/jb.00373-25)
Supplement: Supplementary file 1 [file jb.00373-25-s0001.pdf]

mView^{TM} **REPORT**

Metabolic response of *Pseudomonas aeruginosa* to BIT

UNVA-04-17VW

CLIENT: University of Virginia
Glynis Kolling, PhD

AUTHOR: Patricia A. Sheridan, PhD
APPROVAL: Edward D. Karoly, PhD
DATE: December 22, 2017

METABOLON®

Metabolon, Inc. • 617 Davis Drive, Suite 400, Durham, NC 27713 • (919) 572-1711 www.metabolon.com •
Contact: info@metabolon.com

Table of Contents

Objective 3

Experimental Procedures 3

Results and Biological Interpretation 3

Metabolite Summary and Significantly Altered Biochemicals 3

Biological Interpretation 6

Conclusions 9

Study Parameters 9

Data Quality: Instrument and Process Variability 9

Appendix 10

Metabolon Platform 10

Statistical Methods and Terminology 15

Objective

Purpose of Experiment

The goal of this study was to compare the metabolomic profile in spent media from *Pseudomonas aeruginosa* that has been exposed to 1,2-benzisothiazol-3(2H)-one1,1-dioxide (BIT).

Experimental Procedures

Experimental design

This 72 sample study examined global metabolic profiles in supernatant of *Pseudomonas aeruginosa* treated with BIT. Samples were collected at 0 hours, 5 hours, and 24 hours. Global metabolic profiles were determined from the experimental groups outlined in the table below.

Group	Description	n
0% BIT 0H	supernatant of *P. aeruginosa* untreated 0h	8
0.1% BIT 0H	supernatant of *P. aeruginosa* exposed to 0.1% BIT 0h	8
10% BIT 0H	supernatant of *P. aeruginosa* exposed to 10% BIT 0h	8
0% BIT 5H	supernatant of *P. aeruginosa* untreated 5h	8
0.1% BIT 5H	supernatant of *P. aeruginosa* exposed to 0.1% BIT 5h	8
10% BIT 5H	supernatant of *P. aeruginosa* exposed to 10% BIT 5h	8
0% BIT 24H	supernatant of *P. aeruginosa* untreated 24h	8
0.1% BIT 24H	supernatant of *P. aeruginosa* exposed to 0.1% BIT 24h	8
10% BIT 24H	supernatant of *P. aeruginosa* exposed to 10% BIT 24h	8

Results and Biological Interpretation

Metabolite Summary and Significantly Altered Biochemicals

The present dataset comprises a total of 395 compounds of known identity (named biochemicals). Following log transformation and imputation of missing values, if any, with the minimum observed value for each compound, ANOVA contrasts were used to identify biochemicals that differed significantly between experimental groups. A summary of the

numbers of biochemicals that achieved statistical significance ($p \leq 0.05$), as well as those approaching significance ($0.05 < p < 0.10$), is shown below. Analysis by two-way ANOVA identified biochemicals exhibiting significant interaction and main effects for experimental parameters of treatment and time.

An estimate of the false discovery rate (*q*-value) is calculated to take into account the multiple comparisons that normally occur in metabolomic-based studies. For example, when analyzing 200 compounds, we would expect to see about 10 compounds meeting the $p \leq 0.05$ cut-off by random chance. The *q*-value describes the false discovery rate; a low *q*-value ($q < 0.10$) is an indication of high confidence in a result. While a higher *q*-value indicates diminished confidence, it does not necessarily rule out the significance of a result. Other lines of evidence may be taken into consideration when determining whether a result merits further scrutiny. Such evidence may include a) significance in another dimension of the study, b) inclusion in a common pathway with a highly significant compound, or c) residing in a similar functional biochemical family with other significant compounds. Refer to the Appendix for general definitions and further descriptions of false discovery rate and other statistical tests used at Metabolon.

Statistical Comparisons

Statistically Significant Biochemicals	ANOVA Main Effects			ANOVA Contrasts								
				0% BIT			0.1% BIT			10% BIT		
	Treatment Effect	Time Effect	Treatment : Time Interaction	5H / 0H	24H / 0H	24H / 5H	5H / 0H	24H / 0H	24H / 5H	5H / 0H	24H / 0H	24H / 5H
Total biochemicals p≤0.05	328	323	313	146	263	198	195	238	96	218	214	49
Biochemicals (↑↓)	- - -	- - -	- - -	40 | 106	108 | 155	111 | 87	90 | 105	108 | 130	51 | 45	68 | 150	70 | 144	38 | 11
Total biochemicals 0.05<p<0.10	16	11	12	24	18	21	20	16	25	25	28	15
Biochemicals (↑↓)	- - -	- - -	- - -	8 | 16	11 | 7	15 | 6	4 | 16	3 | 13	12 | 13	5 | 20	9 | 19	11 | 4

Statistical Comparisons

Statistically Significant Biochemicals	ANOVA Contrasts								
	0H			5H			24H		
	0.1% BIT / 0% BIT	10% BIT / 0% BIT	10% BIT / 0.1% BIT	0.1% BIT / 0% BIT	10% BIT / 0% BIT	10% BIT / 0.1% BIT	0.1% BIT / 0% BIT	10% BIT / 0% BIT	10% BIT / 0.1% BIT
Total biochemicals p≤0.05	25	6	10	191	242	223	276	298	228
Biochemicals (↑↓)	22 | 3	5 | 1	1 | 9	124 | 67	105 | 137	56 | 167	153 | 123	137 | 161	63 | 165
Total biochemicals 0.05<p<0.10	26	11	15	20	20	18	15	16	17
Biochemicals (↑↓)	19 | 7	7 | 4	5 | 10	8 | 12	6 | 14	8 | 10	5 | 10	6 | 10	4 | 13

We have also included in the electronic deliverables, a file with data for each biochemical displayed as line plots like that shown in the example figure below.

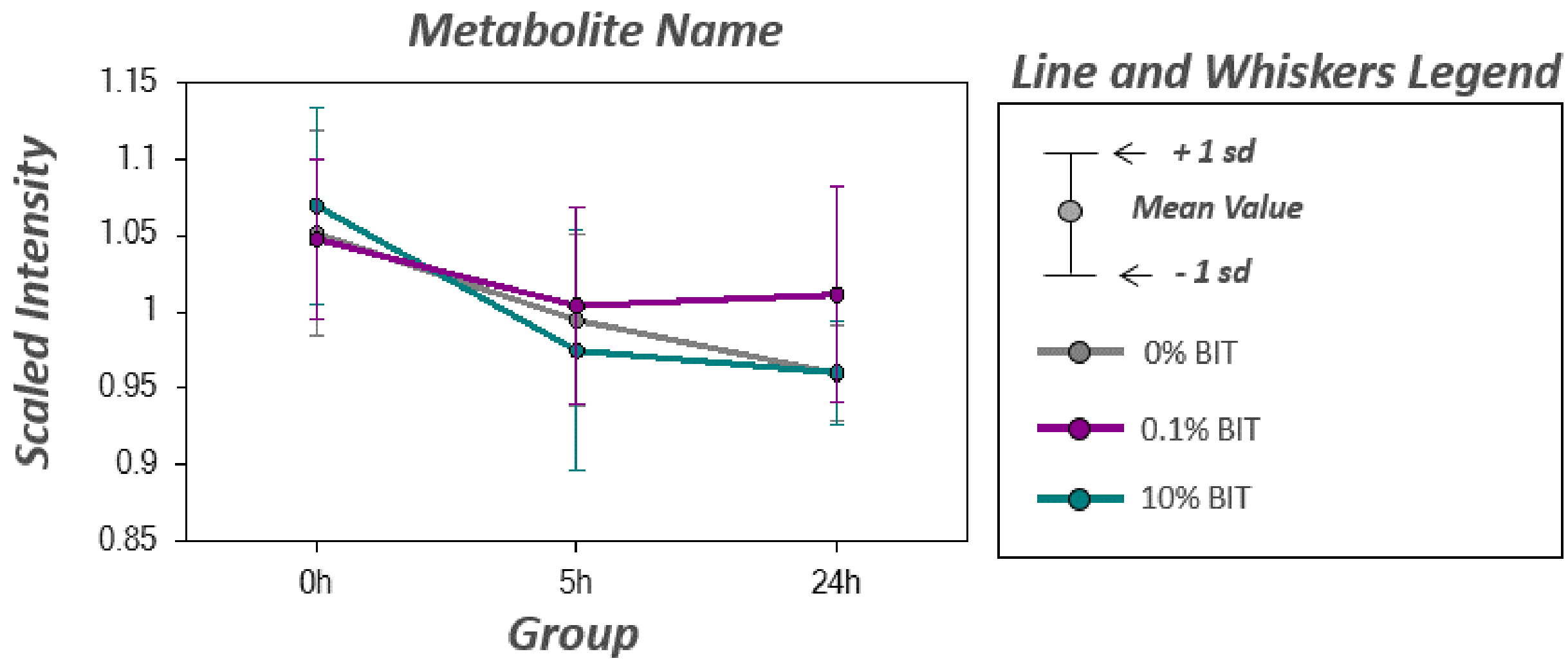

Biological Interpretation

Benzisothiazolinones (BIT) are a common biocides which inhibit the activity of many sulfur-containing enzymes. BITs are used extensively in agricultural, industrial and consumer products and are also incorporated into many products used in the hospital setting to reduce the risk of nosocomial infection. *Pseudomonas aeruginosa* (*P. aeruginosa*) is environmentally ubiquitous with many strains causing acute and chronic infections in humans. *P. aeruginosa* strain 14 (PA14) is a highly virulent clinical isolate that has been widely studied. In the current study, PA14 was exposed to 0, 0.1 or 10% BIT for 0, 5 and 24h. These time points correspond to log phase growth (5h) and stationary phase (24h) in normal, untreated PA14. The media was collected at these time points and sterile filtered. The sterile media was then analyzed on the HD4 Global platform.

All media derived samples were loaded onto the platform in an equivalent manner with no normalization required prior to the statistical analysis. Datasets provided in the mView product can be quite large and contain a great deal of information. Comparison of global biochemical profiles of the media revealed some key metabolic differences as highlighted below. For the discussion below, please refer to graphical illustrations in the accompanying PowerPoint file, or to the statistical tables, box plots, and other graphics found in the accompanying client data table. For convenience, biochemicals are highlighted in bold text in the report when they correspond to the data plots or heatmap figures shown in the accompanying PowerPoint file.

- **Principle component analysis (PCA)** is a mathematical procedure that allows differences across a large set of variables to be represented as a smaller set of variables. A description of the mathematical method is provided in the "Statistical Analysis" section of the report. PCA permits visualization of how individual samples, within a group, cluster with respect to their data-compressed "principle components". As such, this analysis tool aids in determining if samples can be segregated according to group, based on their global metabolite profiles**.** At 0h (prior to treatment) there is no separation among the groups, which supports that there were no significant difference among the metabolomic profiles of the cultures at baseline (**Slide 5**). By 5h, the samples separate by treatment and continue to separate at 24h. In the 10% treatment group, the 5h and 24h time points are close to each other but still form discrete clusters. Overall, there was significant separation among the groups by time and treatment.

- **Amino acid catabolism and excretion.** *Pseudomonas* are capable of using single amino acids as their sole carbon source. These amino acids replenish TCA cycle intermediates at various points to support growth (PMID:4981058). Consistent with this, the 0% BIT group had decreased amino acids in media over time (e.g. **tryptophan**, **lysine** and **leucine**) which would suggest increased import into the cells to support growth (**Slide 6**). In general, 0.1% and 10% BIT treatment resulted in little change in the amounts of amino acids present in the media overtime. This would support that BIT is decreasing the rate of growth in PA14 and thus requires less amino acid nutritive support.

Concomitant with the decrease in amino acids was an increase in acetyl-amino acids (*e.g.* **N2-acetyllysine** and **N-acetylleucine**) and catabolites (*e.g.* **indoleacetate** and **anthranilate**) in the 0% BIT cultures which is constant with an increase in protein turnover and use of the amino acids for growth support. In 0.1% and 10% BIT treated cultures, these increases were not seen suggesting that amino acids catabolism was decreased because the cells were growth inhibited by BIT.

- **Glucose utilization.** *P. aeruginosa* predominantly uses the Entner-Doudoroff pathway for glucose metabolism as they lack phosphofructokinase and phosphogluconate dehydrogenase, key enzymes in the Embden-Meyerhof and Pentose Phosphate Pathways, respectively. In 0% BIT cultures, glucose decreased in the media over time, while there was a decrease in **pyruvate** and little change in **lactate** in the cultures (**Slide 7**). These data are consistent with glucose utilization for growth in PA14 cells. The response in 0.1% and 10% Bit treated PA14 cultures was more complicated. In 0.1% media, pyruvate levels rose significantly over time. In the 10% BIT media cultures, pyruvate and lactate accumulated. BIT inhibits both pyruvate dehydrogenase (PDH) and lactate dehydrogenase (LDH; Williams, T.M. PowerPlant Chemistry, 2007). There may be a dose response effect on this inhibition that would explain the increase in lactate in 10%, but not 0.1% BIT PA14 media, while an inhibition of PDH would explain the increased accumulation of pyruvate. Taken together, these data support that BIT inhibits PA14 growth and part of the mechanism of action is targeting enzymes responsible for linking glucose utilization with energy production via the TCA cycle. In addition, dying PA14 cells may release these compounds into the media.

- **TCA cycle and electron transport.** The TCA cycle links the catabolism of carbohydrates, lipids and some amino acids to produce ATP via supplying reducing equivalents through oxidative phosphorylation. *P. aeruginosa,* is not a primary fermenter and as such relies on the TCA cycle for energy generation (PMID:25848053). BIT inhibits α-ketoglutarate dehydrogenase (α-KGDH), succinate dehydrogenase (SDH) and NADH dehydrogenase (PowerPlant Chemistry, 2007) and studies have demonstrated that the conversion of α-ketoglutarate to succinyl-CoA and succinate to fumarate are essential for P. aeruginosa growth and survival *(*PMID:25848053). Consistent with this mechanism of action, there was an accumulation of **α-ketoglutarate** and **succinate** in 0.1% and 10% BIT PA14 culture media that was not observed in the 0% cultures (**Slide 8**). Interestingly, **fumarate** decreased in 0% and 0.1% BIT cells, suggesting it was taken up by the cells to support malate production, while **malate** increased in 0% cultures. It is possible that the effect on PA14 growth is such that the cells are too damaged (or dead) to import malate for use in the TCA cycle. Inhibition of NADH dehydrogenase by BIT leads to the accumulation of **NAD+** in 0.1% and 10% BIT media (**Slide 9**). Slide 9 also provides an integrative overview of the targets of BIT. Taken together, these data are consistent with the known mechanism of action for BIT.

- **Methionine and glutathione.** Methionine is an essential amino acid for most *P. aeruginosa* isolates. Methionine is metabolized to cysteine which supports the

maintenance of redox homeostasis through glutathione and gamma-glutamyl amino acids. In *E. coli*, BIT-induced growth inhibition can be reversed by adding glutathione and/or cysteine to the culture. These thiol-containing compounds may act as a competitive inhibitor to reduce binding of BIT to the thiol-containing enzymes that were discussed above (PMID:2292520). One could hypothesis that upregulation of the methionine-glutathione system would be a mechanism used by PA14 to overcome BIT growth inhibition. In the current study, there were clear differences in 0.1% and 10% BIT cultures. In 0.1% cultures, **methionine** was relatively unchanged overtime, while **cysteine** decreased, likely entering the PA14 cells to support glutathione (**Slide 10**). **Oxidized glutathione (GSSG)** decreased in the 0.1% culture, while **ophthalmate** initially increased then declined and **γ-glutamylcysteine** significantly decreased by 5h. Because *P. aeruginosa* use glutamate-cysteine ligase (gshA) and glutathione synthetase (gshB) in a two-step process to produce glutathione, these changes suggest an increase in glutathione synthetase activity which may indicate an attempt by the PA14 cells to produce more glutathione in response to BIT. In 10% PA14 culture media, methionine, and cysteine both decreased by 5h, as did GSSG and γ-glutamylcysteine, while ophthalmate increased significantly over time. These data are consistent with an increased demand for glutathione and attempts to increase glutathione production.

- **Quorum sensing and membrane breakdown**. Quorum sensing (QS) is a method of communication between bacteria that controls population dependent gene expression. Monitoring the density of the bacterial population facilitates optimum growth rates and survival. PA14 uses two QS systems: The N-acylhomoserine lactone-dependent QS which results in the activation of genes including RhII which encodes N-butanoyl-homoserine lactone. This QS system is linked to another QS which produces 2-heptyl-1H-quinolin-4-one (HHQ). In the current study, PA14 media from 0% BIT cultures had increased **N-butanoyl-homoserine lactone** and **HHQ (Slide 11)**. In 0.1% BIT media, N-butanoyl-homoserine lactone decreased significantly over time, while in 10% BIT media, N-butanoyl-homoserine lactone decreased and HHQ significantly increased. These data are suggestive of differences in the population density of the PA14 in each of these cultures. Additionally, there was an accumulation of membrane components (e.g. **glycerophosphorylcholine**, **glycerophosphoethanolamine** and **phosphoethanolamine**) in the 0.1% and 10% BIT media which may suggest that these are fragments from dead or dying bacteria. Taken together, these data provide further support of BIT-induced changes in growth kinetics and increased PA14 death.

Conclusions

In conclusion, these data support that BIT functions as a biocide which slows PA14 growth and increased bacterial cell death (amino acid use, quorum sensing, membrane degradation) and these data confirm the mechanism by which BIT promotes PA14 death (TCA cycle, oxidative phosphorylation and glutathione pathways). There were many other significant changes which could not be addressed due to the high level overview nature of this report. However, many of these changes coincide with differences in growth dynamics and cell death (nucleotide, nicotinate and nicotinamide metabolism, for example).

There are several paths forward for these studies. Correlating the cell numbers and growth kinetics with metabolites would help to determine which changes are associated with a defense response to BIT and those which are markers of dead or dying cells. Additionally, metabolomics on the PA14 cells themselves would confirm the mechanism of action for BIT, much of which is inferred in this report based on the media data. Finally, a rescue experiment with the addition of glutathione or cysteine to the cultures would extend previous studies performed in *E. coli* into *Pseudomonas aeruginosa.*

Study Parameters

Data Quality: Instrument and Process Variability

QC Sample	*Measurement*	*Median RSD*
Internal Standards	Instrument Variability	6%
Endogenous Biochemicals	Total Process Variability	9%

Instrument variability was determined by calculating the median relative standard deviation (RSD) for the internal standards that were added to each sample prior to injection into the mass spectrometers. Overall process variability was determined by calculating the median RSD for all endogenous metabolites (i.e., non-instrument standards) present in 100% of the Client Matrix samples, which are technical replicates of pooled client samples. Values for instrument and process variability meet Metabolon's acceptance criteria as shown in the table above.

Metabolon Platform

Sample Accessioning: Following receipt, samples were inventoried and immediately stored at -80ºC. Each sample received was accessioned into the Metabolon LIMS system and was assigned by the LIMS a unique identifier that was associated with the original source identifier only. This identifier was used to track all sample handling, tasks, results, etc. The samples (and all derived aliquots) were tracked by the LIMS system. All portions of any sample were automatically assigned their own unique identifiers by the LIMS when a new task was created; the relationship of these samples was also tracked. All samples were maintained at -80ºC until processed.

Sample Preparation: Samples were prepared using the automated MicroLab STAR® system from Hamilton Company. Several recovery standards were added prior to the first step in the extraction process for QC purposes. To remove protein, dissociate small molecules bound to protein or trapped in the precipitated protein matrix, and to recover chemically diverse metabolites, proteins were precipitated with methanol under vigorous shaking for 2 min (Glen Mills GenoGrinder 2000) followed by centrifugation. The resulting extract was divided into five fractions: two for analysis by two separate reverse phase (RP)/UPLC-MS/MS methods with positive ion mode electrospray ionization (ESI), one for analysis by RP/UPLC-MS/MS with negative ion mode ESI, one for analysis by HILIC/UPLC-MS/MS with negative ion mode ESI, and one sample was reserved for backup. Samples were placed briefly on a TurboVap® (Zymark) to remove the organic solvent. The sample extracts were stored overnight under nitrogen before preparation for analysis.

QA/QC: Several types of controls were analyzed in concert with the experimental samples: a pooled matrix sample generated by taking a small volume of each experimental sample (or alternatively, use of a pool of well-characterized human plasma) served as a technical replicate throughout the data set; extracted water samples served as process blanks; and a cocktail of QC standards that were carefully chosen not to interfere with the measurement of endogenous compounds were spiked into every analyzed sample, allowed instrument performance monitoring and aided chromatographic alignment. Tables 1 and 2 describe these QC samples and standards. Instrument variability was determined by calculating the median relative standard deviation (RSD) for the standards that were added to each sample prior to injection into the mass spectrometers. Overall process variability was determined by calculating the median RSD for all endogenous metabolites (i.e., non-instrument standards) present in 100% of the pooled matrix samples. Experimental samples were randomized across the platform run with QC samples spaced evenly among the injections, as outlined in Figure 1.

Table 1: Description of Metabolon QC Samples

Type	Description	Purpose
MTRX	Large pool of human plasma maintained by Metabolon that has been characterized extensively.	Assure that all aspects of the Metabolon process are operating within specifications.
CMTRX	Pool created by taking a small aliquot from every customer sample.	Assess the effect of a non-plasma matrix on the Metabolon process and distinguish biological variability from process variability.
PRCS	Aliquot of ultra-pure water	Process Blank used to assess the contribution to compound signals from the process.
SOLV	Aliquot of solvents used in extraction.	Solvent Blank used to segregate contamination sources in the extraction.

Table 2: Metabolon QC Standards

Type	Description	Purpose
RS	Recovery Standard	Assess variability and verify performance of extraction and instrumentation.
IS	Internal Standard	Assess variability and performance of instrument.

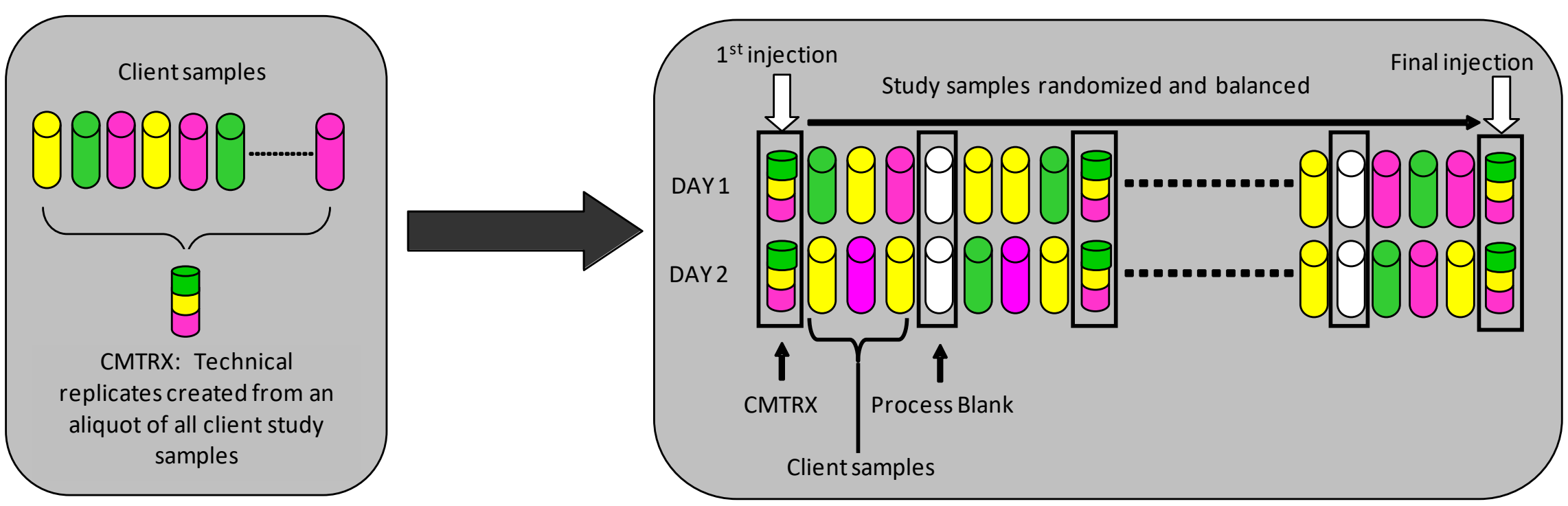

Figure 1. Preparation of client-specific technical replicates. A small aliquot of each client sample (colored cylinders) is pooled to create a CMTRX technical replicate sample (multi-colored cylinder), which is then injected periodically throughout the platform run. Variability among consistently detected biochemicals can be used to calculate an estimate of overall process and platform variability.

Ultrahigh Performance Liquid Chromatography-Tandem Mass Spectroscopy (UPLC-MS/MS): All methods utilized a Waters ACQUITY ultra-performance liquid chromatography (UPLC) and a Thermo Scientific Q-Exactive high resolution/accurate mass spectrometer interfaced with a heated electrospray ionization (HESI-II) source and Orbitrap mass analyzer operated at 35,000 mass resolution. The sample extract was dried then reconstituted in solvents compatible to each of the four methods. Each reconstitution solvent contained a series of standards at fixed concentrations to ensure injection and chromatographic consistency. One aliquot was analyzed using acidic positive ion conditions, chromatographically optimized for more hydrophilic compounds. In this method, the extract was gradient eluted from a C18 column (Waters UPLC BEH C18-2.1x100 mm, 1.7 µm) using water and methanol, containing 0.05% perfluoropentanoic acid (PFPA) and 0.1% formic acid (FA). Another aliquot was also analyzed using acidic positive ion conditions, however it was chromatographically optimized for more hydrophobic compounds. In this method, the extract was gradient eluted from the same afore mentioned C18 column using methanol, acetonitrile, water, 0.05% PFPA and 0.01% FA and was operated at an overall higher organic content. Another aliquot was analyzed using basic negative ion optimized conditions using a separate dedicated C18 column. The basic extracts were gradient eluted from the column using methanol and water, however with 6.5mM Ammonium Bicarbonate at pH 8. The fourth aliquot was analyzed via negative ionization following elution from a HILIC column (Waters UPLC BEH Amide 2.1x150 mm, 1.7 µm) using a gradient consisting of water and acetonitrile with 10mM Ammonium Formate, pH 10.8. The MS analysis alternated between MS and data-dependent MS^n scans using dynamic exclusion. The scan range varied slighted between methods but covered 70-1000 m/z. Raw data files are archived and extracted as described below.

Bioinformatics: The informatics system consisted of four major components, the Laboratory Information Management System (LIMS), the data extraction and peak-identification software, data processing tools for QC and compound identification, and a collection of information interpretation and visualization tools for use by data analysts. The hardware and software foundations for these informatics components were the LAN backbone, and a database server running Oracle 10.2.0.1 Enterprise Edition.

LIMS: The purpose of the Metabolon LIMS system was to enable fully auditable laboratory automation through a secure, easy to use, and highly specialized system. The scope of the Metabolon LIMS system encompasses sample accessioning, sample preparation and instrumental analysis and reporting and advanced data analysis. All of the subsequent software systems are grounded in the LIMS data structures. It has been modified to leverage and interface with the in-house information extraction and data visualization systems, as well as third party instrumentation and data analysis software.

Data Extraction and Compound Identification: Raw data was extracted, peak-identified and QC processed using Metabolon's hardware and software. These systems are built on a web-service platform utilizing Microsoft's .NET technologies, which run on high-performance application servers and fiber-channel storage arrays in clusters to provide active failover and load-balancing. Compounds were identified by comparison to library entries of purified

standards or recurrent unknown entities. Metabolon maintains a library based on authenticated standards that contains the retention time/index (RI), mass to charge ratio (*m/z)*, and chromatographic data (including MS/MS spectral data) on all molecules present in the library. Furthermore, biochemical identifications are based on three criteria: retention index within a narrow RI window of the proposed identification, accurate mass match to the library +/- 10 ppm, and the MS/MS forward and reverse scores between the experimental data and authentic standards. The MS/MS scores are based on a comparison of the ions present in the experimental spectrum to the ions present in the library spectrum. While there may be similarities between these molecules based on one of these factors, the use of all three data points can be utilized to distinguish and differentiate biochemicals. More than 3300 commercially available purified standard compounds have been acquired and registered into LIMS for analysis on all platforms for determination of their analytical characteristics. Additional mass spectral entries have been created for structurally unnamed biochemicals, which have been identified by virtue of their recurrent nature (both chromatographic and mass spectral). These compounds have the potential to be identified by future acquisition of a matching purified standard or by classical structural analysis.

Curation: A variety of curation procedures were carried out to ensure that a high quality data set was made available for statistical analysis and data interpretation. The QC and curation processes were designed to ensure accurate and consistent identification of true chemical entities, and to remove those representing system artifacts, mis-assignments, and background noise. Metabolon data analysts use proprietary visualization and interpretation software to confirm the consistency of peak identification among the various samples. Library matches for each compound were checked for each sample and corrected if necessary.

Metabolite Quantification and Data Normalization: Peaks were quantified using area-under-the-curve. For studies spanning multiple days, a data normalization step was performed to correct variation resulting from instrument inter-day tuning differences. Essentially, each compound was corrected in run-day blocks by registering the medians to equal one (1.00) and normalizing each data point proportionately (termed the “block correction”; Figure 2). For studies that did not require more than one day of analysis, no normalization is necessary, other than for purposes of data visualization. In certain instances, biochemical data may have been normalized to an additional factor (e.g., cell counts, total protein as determined by Bradford assay, osmolality, etc.) to account for differences in metabolite levels due to differences in the amount of material present in each sample.

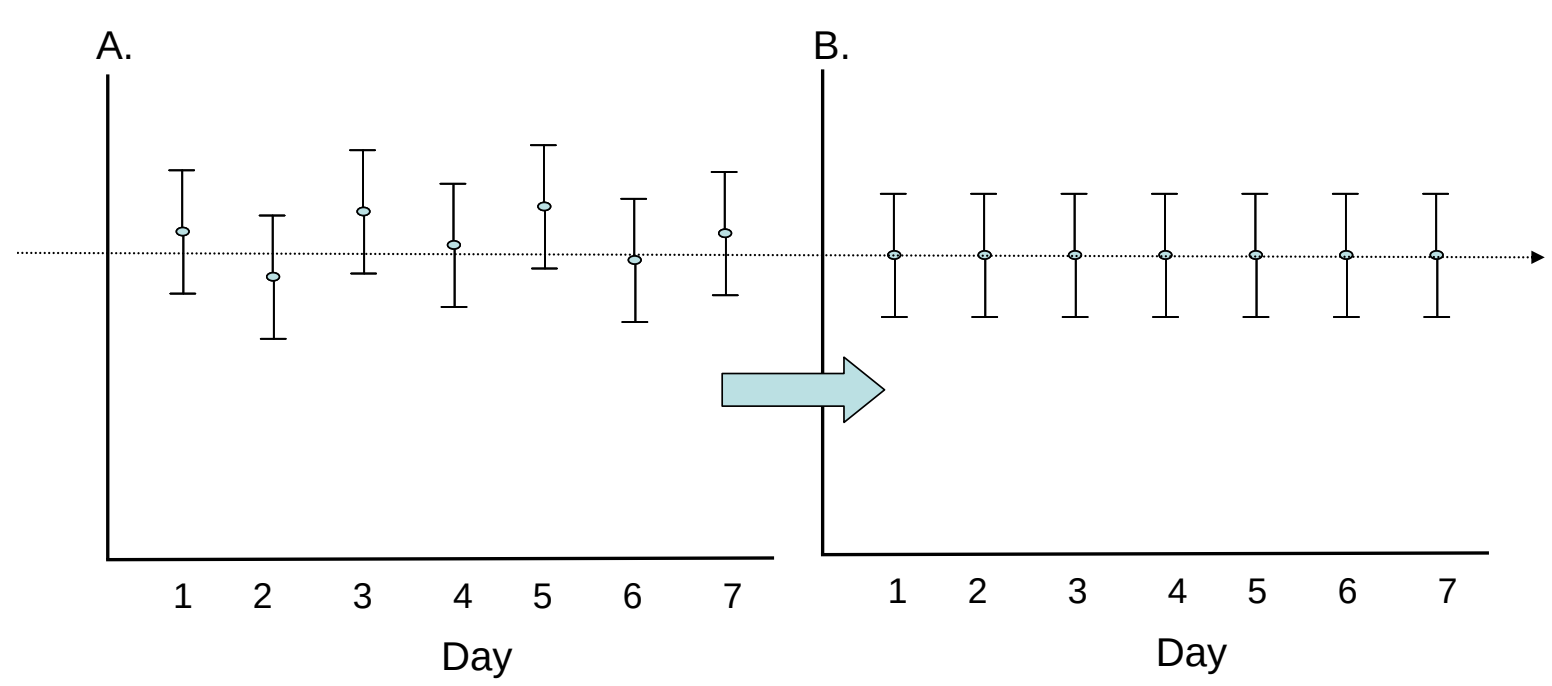

Figure 2: Visualization of data normalization steps for a multiday platform run.

Statistical Methods and Terminology

Statistical Calculations: For many studies, two types of statistical analysis are usually performed: (1) significance tests and (2) classification analysis. Standard statistical analyses are performed in ArrayStudio on log transformed data. For those analyses not standard in ArrayStudio, the programs R (http://cran.r-project.org/) or JMP are used. Below are examples of frequently employed significance tests and classification methods followed by a discussion of p- and q-value significance thresholds.

1. **Welch's two-sample *t*-test**

   Welch's two-sample *t*-test is used to test whether two unknown means are different from two independent populations.

   This version of the two-sample *t*-test allows for unequal variances (variance is the square of the standard deviation) and has an *approximate t*-distribution with degrees of freedom estimated using Satterthwaite's approximation. The test statistic is given by $t=(\bar{x}_1-\bar{x}_2)/\sqrt{s_1^2/n_1+s_2^2/n_2}$, and the degrees of freedom is given by $\left(\frac{s_1^2}{n_1}+\frac{s_2^2}{n_2}\right)^2 / \left(\frac{\left(\frac{s_1^2}{n_1}\right)^2}{n_1-1}+\frac{\left(\frac{s_2^2}{n_2}\right)^2}{n_2-1}\right)$, where $\bar{x}_1$, $\bar{x}_2$ are the sample means, s_1, s_2, are the sample standard deviations, and n_1, n_2 are the samples sizes from groups 1 and 2, respectively. We typically use a two-sided test (tests whether the means are different) as opposed to a one-sided test (tests whether one mean is greater than the other).

2. **Matched pairs *t*-test**

   The matched pairs *t*-test is used to test whether two unknown means are different from paired observations taken on the same subjects.

   The matched pairs *t*-test is equivalent to the one-sample *t*-test performed on the differences of the observations taken on each subject (i.e., calculate $(x_1 - x_2)$ for each subject; test whether the mean difference is zero or not). The test statistic is given by $t = (\bar{x}_1-\bar{x}_2)/n$, with *n* – 1 degrees of freedom, where $\bar{x}_1$, $\bar{x}_2$ are the sample means for groups 1 and 2, respectively, s_d is the standard deviation of the differences, *n* is the number of *subjects* (so there are 2*n* observations).

3. **One-way ANOVA**

   ANOVA stands for analysis of variance. For ANOVA, it is assumed that all populations have the same variances. One-way ANOVA is used to test whether at least two unknown means are all equal or whether at least one pair of means is different. For the case of two means, ANOVA gives the same result as a two-sided *t*-test with a pooled estimate of the variance.

An ANOVA uses an F-test which has two parameters – the numerator degrees of freedom and the denominator degrees of freedom. The degrees of freedom in the numerator are equal to $g - 1$, where g is the number of groups. If n is the total number of observations ($n_1 + n_2$), then, the denominator degrees of freedom is equal to $n - g$. The F-statistic is the ratio of the between-groups variance to the within-groups variance, hence the higher the F-statistic the more evidence we have that the means are different.

Often within ANOVA, one performs linear contrasts for specific comparisons of interest. For example, suppose we have three groups A, B, C, then examples of some contrasts are A vs. B, the average of A and B vs. C, etc. For single-degree of freedom contrasts, these give the same result as a two-sided *t*-test with the pooled estimate of the variance from the ANOVA and degrees of freedom $n - g$. Below, we show the three formulas for A vs. B from a three group design as shown above. The numerator is same in each case, but the denominator differs by the estimates of the variances, and the degrees of freedom are different for each (if the theoretical assumptions hold, then the contrast has the most power, as it has the largest degrees of freedom).

Welch's two-sample *t*-test

By $t = (\bar{x}_A - \bar{x}_B)/\sqrt{s_A^2/n_A + s_B^2/n_B}$, and the degrees of freedom is given by $\left(\frac{s_A^2}{n_A} + \frac{s_B^2}{n_B}\right)^2 / \left(\frac{\left(\frac{s_A^2}{n_A}\right)^2}{n_A - 1} + \frac{\left(\frac{s_B^2}{n_B}\right)^2}{n_B - 1}\right)$

Two-sample *t*-test with pooled estimate of variance from A and B

$$t = (\bar{x}_A - \bar{x}_B)/\sqrt{s_{AB}^2(1/n_A + /n_B)}$$

where $s_{AB}^2 = \left((n_A - 1)s_A^2 + (n_B - 1)s_B^2\right)/(n_A + n_B - 2)$, where the degrees of freedom is $n_A + n_B - 2$.

The contrast from the ANOVA,

$$t = (\bar{x}_A - \bar{x}_B)/\sqrt{s^2(1/n_A + /n_B)}$$

where $s^2 = \left((n_A - 1)s_A^2 + (n_B - 1)s_B^2 + (n_C - 1)s_C^2\right)/(n_A + n_B + n_C - 3)$, where the degrees of freedom is given by where the degrees of freedom is $n_A + n_B + n_C - 3$.

4. Two-way ANOVA

ANOVA stands for analysis of variance. For ANOVA, it is assumed that all populations have the same variances. For a two-way ANOVA, three statistical tests are typically performed: the main effect of each factor and the interaction. Suppose we have two factors A and B, where A represent the genotype and B represent the diet in a mouse study. Suppose each of these factors has two levels (A: wild type, knock out; B: standard diet, high fat diet). For this example, there are 4 combinations ("treatments"): A1B1, A1B2, A2B1, A2B2. The overall ANOVA F-test gives the p-value for testing

whether all four of these means are equal or whether at least one pair is different. However, we are also interested in the effect of the genotype and diet. A main effect is a contrast that tests one factor across the levels of the other factor. Hence the A main effect compares (A1B1 + A1B2)/2 vs. (A2B1 + A2B2)/2, and the B-main effect compares (A1B1 + A2B2)/2 vs. (A1B2 + A2B2)/2. The interaction is a contrast that tests whether the mean difference for one factor depends on the level of the other factor, which is (A1B2 + A2B1)/2 vs. (A1B1 + A2B2)/2.

Some sample plots follow. For the first plot, there is a B main effect, but no A main effect and no interaction, as the effect of B does not depend on the level of A. For the second plot, notice how the mean difference for B is the same at each level of A and the difference in A is the same for each level of B, hence there is no statistical interaction. The final plot also has main effects for A and B, but here also has an interaction: we see the effect of B depends on the level of A (0 for A1 but 2 for A2), i.e., the effect of the diet depends on the genotype. We also see here the interpretation of the main effects depends on whether there is an interaction or not.

Main Effect for B, but no Main Effect for A, no Interaction

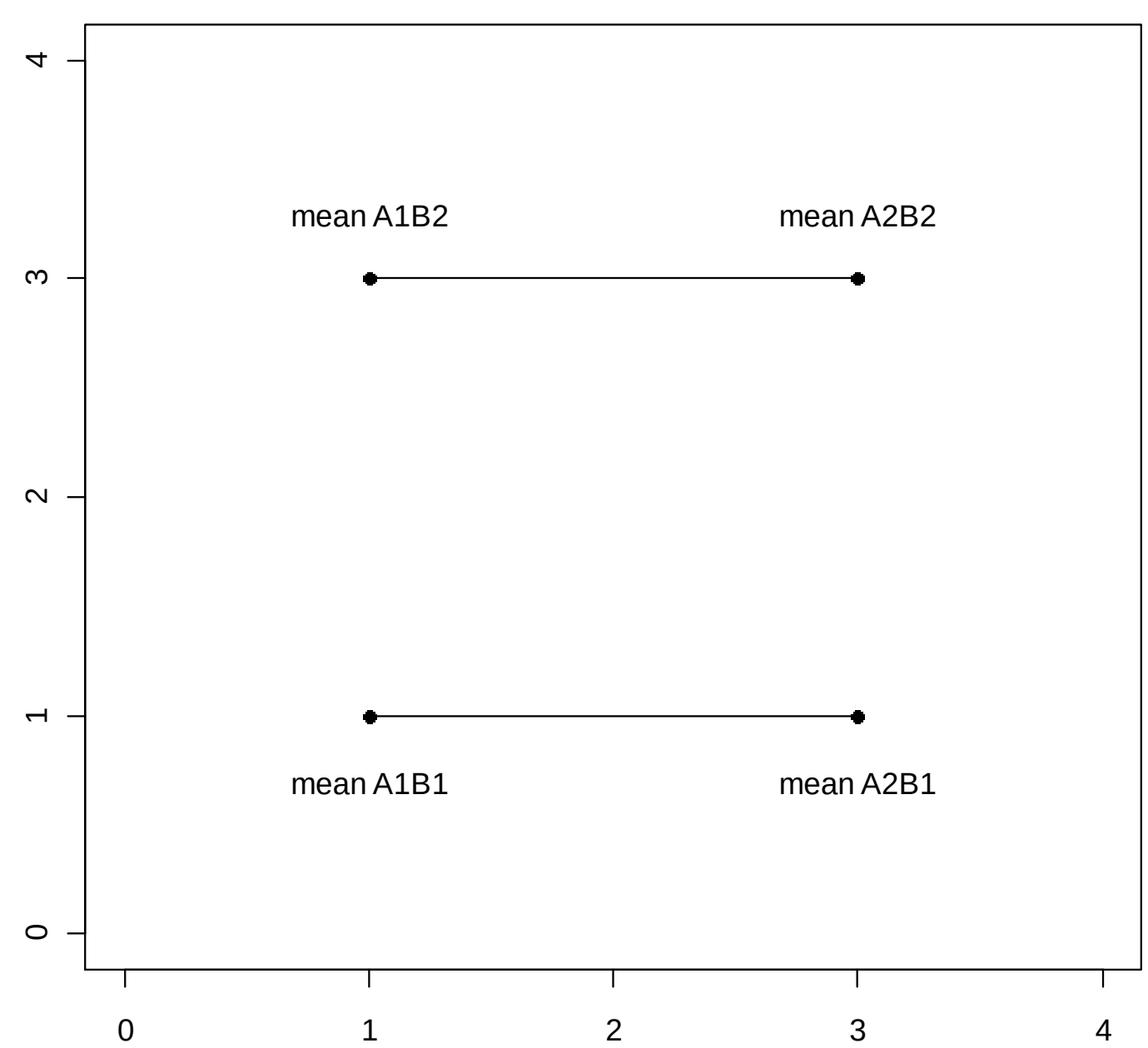

Main Effect for A, Main Effect for B, No Interaction

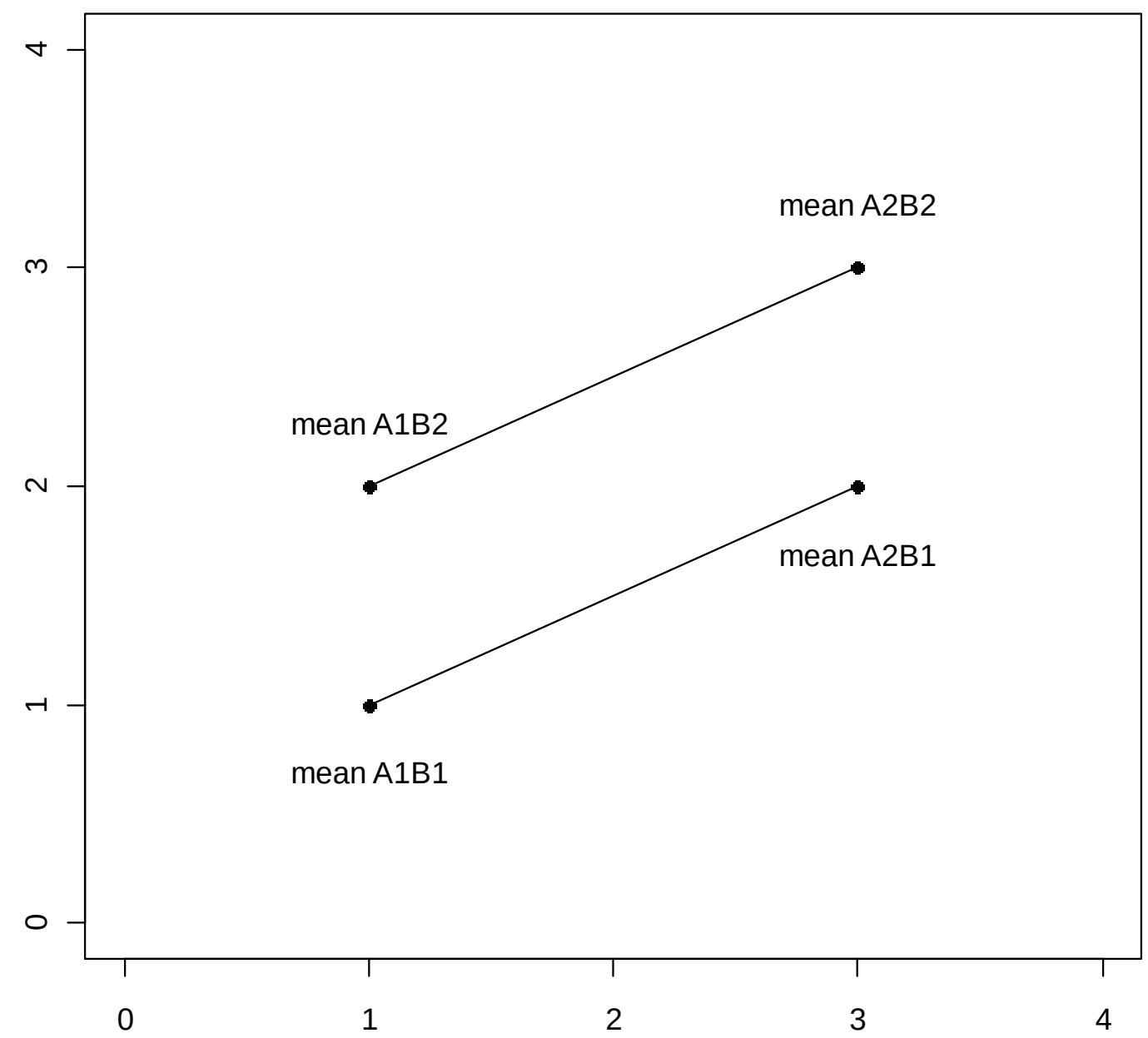

Main Effect for A, Main Effect for B, with Interaction

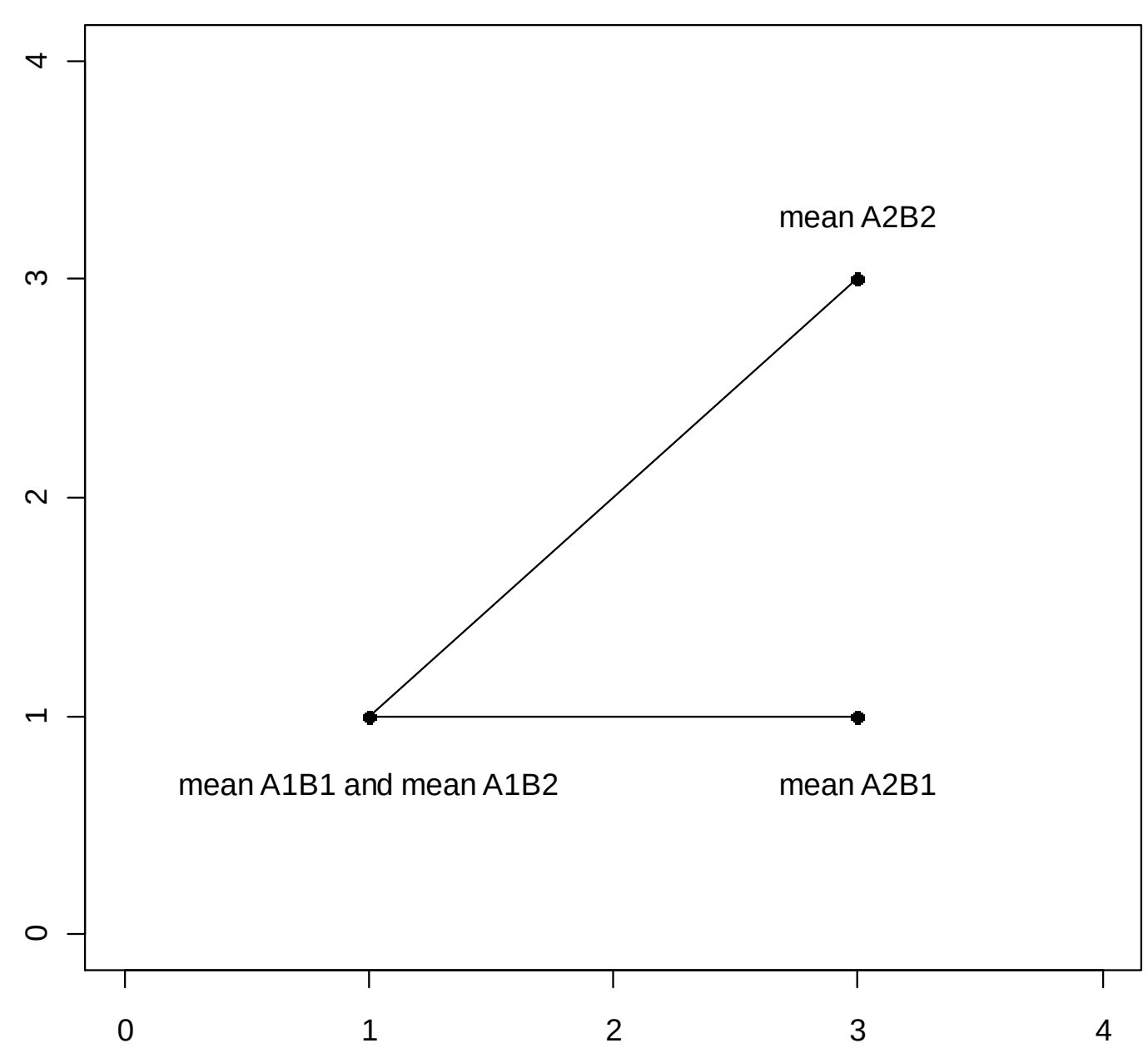

5. **Two-way Repeated Measures ANOVA**

This is typically an ANOVA where one factor is applied to each subject and the second factor is a time point. See two-way ANOVA as many of the details are similar except that the model takes into account the repeated measures, i.e., the treatments are given to the same subject over time. The two main effects and the interaction are assessed, with particular interest to the interaction, as this shows where the time profiles are parallel or not for the treatments (parallel mean no interaction).

One additional note, the standard analysis assumes a condition referred to as compound symmetry, which assumes the correlation between each pair of levels of the repeated-measures factor is the same. Thus, for the case of time, it assumes the correlation is the same between time points 1 and 2, 1 and 3, and 2 and 3.

6. **Correlation**

Correlation measures the strength and direction of a *linear* association between two variables. The statistical test for correlation tests whether the true correlation is zero or not.

The square of the correlation is the percentage of the total variation explained by a linear relationship between the two variables. Thus, with large sample sizes there may be a sample correlation of 0.1 that is statistically significant. This means we have high confidence that the true correlation is zero, however, only 100*(0.1*0.1)% = 1% of the variation of one variable is explained by a linear relationship with the other variable, so while there is an association, it has little predictive ability.

7. **Hotelling's T^2 test**

The Hotelling's T^2 test is a multivariate generalization of the *t*-test, but here we are testing whether the mean vectors are different or not (the vector consists of multiple metabolites).

The Hotelling statistic is: $t^2 = \left(\frac{n_x n_y}{n_x+n_y}\right) * (\overline{\boldsymbol{x}} - \overline{\boldsymbol{y}})^T \boldsymbol{S}^{-1} (\overline{\boldsymbol{x}} - \overline{\boldsymbol{y}})$, where n_x and n_y are the numbers of samples in each group, $\overline{\boldsymbol{x}}$ is the mean vector of the variables from group 1, $\overline{\boldsymbol{y}}$ is the mean vector of variables from group 2 and **S** is the pooled estimate of the variance-covariance matrix of the variables. This analysis assumes the underlying variance-covariance matrix is the same for each group. Notice that in the case of uncorrelated variables, this is simply a weighted average of the squared mean differences with weights inversely proportional to the sample variances (i.e., the metabolites less variable within a group are given higher weights).

8. **p-values**

For statistical significance testing, p-values are given. The lower the p-value, the more evidence we have that the null hypothesis (typically that two population means are

equal) is not true. If "statistical significance" is declared for p-values less than 0.05, then 5% of the time we incorrectly conclude the means are different, when actually they are the same.

The p-value is the probability that the test statistic is at least as extreme as observed in this experiment given that the null hypothesis is true. Hence, the more extreme the statistic, the lower the p-value and the more evidence the data gives against the null hypothesis.

9. q-values

The level of 0.05 is the false positive rate when there is one test. However, for a large number of tests we need to account for false positives. There are different methods to correct for multiple testing. The oldest methods are family-wise error rate adjustments (Bonferroni, Tukey, etc.), but these tend to be extremely conservative for a very large number of tests. With gene arrays, using the False Discovery Rate (FDR) is more common. The family-wise error rate adjustments give one a high degree of confidence that there are zero false discoveries. However, with FDR methods, one can allow for a small number of false discoveries. The FDR for a given set of compounds can be estimated using the q-value (see Storey J and Tibshirani R. (2003) Statistical significance for genomewide studies. Proc. Natl. Acad. Sci. USA 100: 9440-9445; PMID: 12883005).

In order to interpret the q-value, the data must first be sorted by the p-value then choose the cutoff for significance (typically $p<0.05$). The q-value gives the false discovery rate for the selected list (i.e., an estimate of the proportion of false discoveries for the list of compounds whose p-value is below the cutoff for significance). For Table 1 below, if the whole list is declared significant, then the false discovery rate is approximately 10%. If everything from Compound 079 and above is declared significant, then the false discovery rate is approximately 2.5%.

Table 1: Example of q-value interpretation

Compound	*p*-value	*q*-value
Compound 103	0.0002	0.0122
Compound 212	0.0004	0.0122
Compound 076	0.0004	0.0122
Compound 002	0.0005	0.0122
Compound 168	0.0006	0.0122
Compound 079	0.0016	0.0258
Compound 113	0.0052	0.0631
Compound 050	0.0053	0.0631
Compound 098	0.0061	0.0647
Compound 267	0.0098	0.0939

10. Random Forest

Random forest is a supervised classification technique based on an ensemble of decision trees (see Breiman L. (2001) Random Forests. Machine Learning. 45: 5-32; http://link.springer.com/article/10.1023%2FA%3A1010933404324). For a given decision tree, a random subset of the data with identifying true class information is

selected to build the tree ("bootstrap sample" or "training set"), and then the remaining data, the "out-of-bag" (OOB) variables, are passed down the tree to obtain a class prediction for each sample. This process is repeated thousands of times to produce the forest. The final classification of each sample is determined by computing the class prediction frequency ("votes") for the OOB variables over the whole forest. For example, suppose the random forest consists of 50,000 trees and that 25,000 trees had a prediction for sample 1. Of these 25,000, suppose 15,000 trees classified the sample as belonging to Group A and the remaining 10,000 classified it as belonging to Group B. Then the votes are 0.6 for Group A and 0.4 for Group B, and hence the final classification is Group A. This method is unbiased since the prediction for each sample is based on trees built from a subset of samples that do not include that sample. When the full forest is grown, the class predictions are compared to the true classes, generating the "OOB error rate" as a measure of prediction accuracy. Thus, the prediction accuracy is an unbiased estimate of how well one can predict sample class in a new data set. Random forest has several advantages – it makes no parametric assumptions, variable selection is not needed, it does not overfit, it is invariant to transformation, and it is fairly easy to implement with R.

To determine which variables (biochemicals) make the largest contribution to the classification, a "variable importance" measure is computed. We use the "Mean Decrease Accuracy" (MDA) as this metric. The MDA is determined by randomly permuting a variable, running the observed values through the trees, and then reassessing the prediction accuracy. If a variable is not important, then this procedure will have little change in the accuracy of the class prediction (permuting random noise will give random noise). By contrast, if a variable is important to the classification, the prediction accuracy will drop after such a permutation, which we record as the MDA. Thus, the random forest analysis provides an "importance" rank ordering of biochemicals; we typically output the top 30 biochemicals in the list as potentially worthy of further investigation.

11. Hierarchical Clustering

Hierarchical clustering is an unsupervised method for clustering the data, and can show large-scale differences. There are several types of hierarchical clustering and many distance metrics that can be used. A common method is complete clustering using the Euclidean distance, where each sample is a vector with all of the metabolite values. The differences seen in the cluster may be unrelated to the treatment groups or study design.

12. Principal Components Analysis (PCA)

Principal components analysis is an unsupervised analysis that reduces the dimension of the data. Each principal component is a linear combination of every metabolite and the principal components are uncorrelated. The number of principal components is equal to the number of observations.

The first principal component is computed by determining the coefficients of the metabolites that maximizes the variance of the linear combination. The second component finds the coefficients that maximize the variance with the condition that the second component is orthogonal to the first. The third component is orthogonal to the first two components and so on. The total variance is defined as the sum of the variances of the predicted values of each component (the variance is the square of the standard deviation), and for each component, the proportion of the total variance is computed. For example, if the standard deviation of the predicted values of the first principal component is 0.4 and the total variance = 1, then 100*0.4*0.4/1 = 16% of the total variance is explained by the first component. Since this is an unsupervised method, the main components may be unrelated to the treatment groups, and the "separation" does not give an estimate of the true predictive ability.
