# Supplementary material for: Integrating multi-omics data with network modeling to characterize Pseudomonas aeruginosa persister cell metabolism
Source: J Bacteriol. 2026 Jul 20;208(8):e00373-25. doi: 10.1128/jb.00373-25 (PMC13492110; doi:10.1128/jb.00373-25)
Supplement: Supplementary file 2 [file jb.00373-25-s0002.pdf]

Filename: SampleQC-01-48- CG1710274_R2-11102017.cRNA

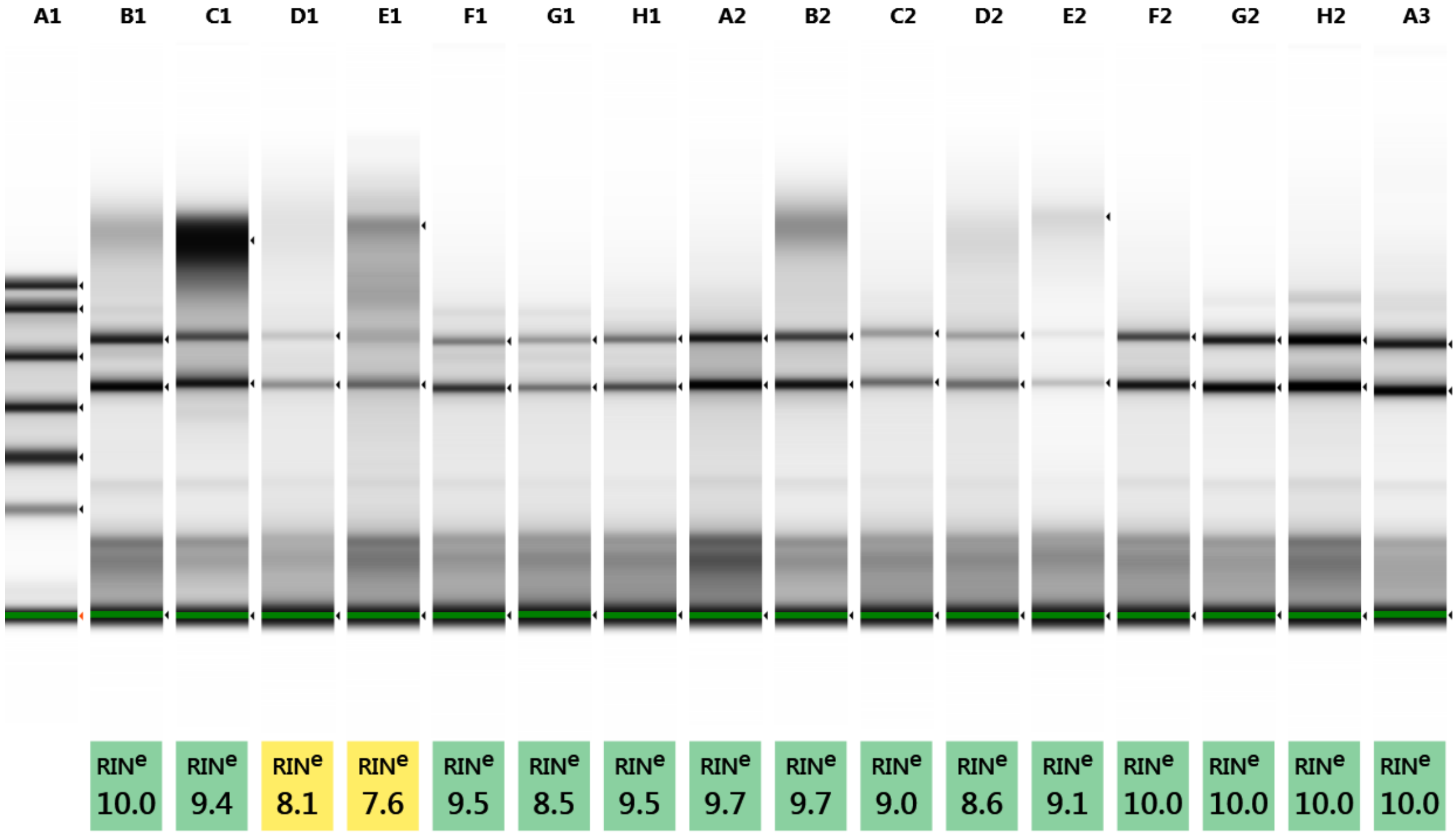

Default image (Contrast 100%)

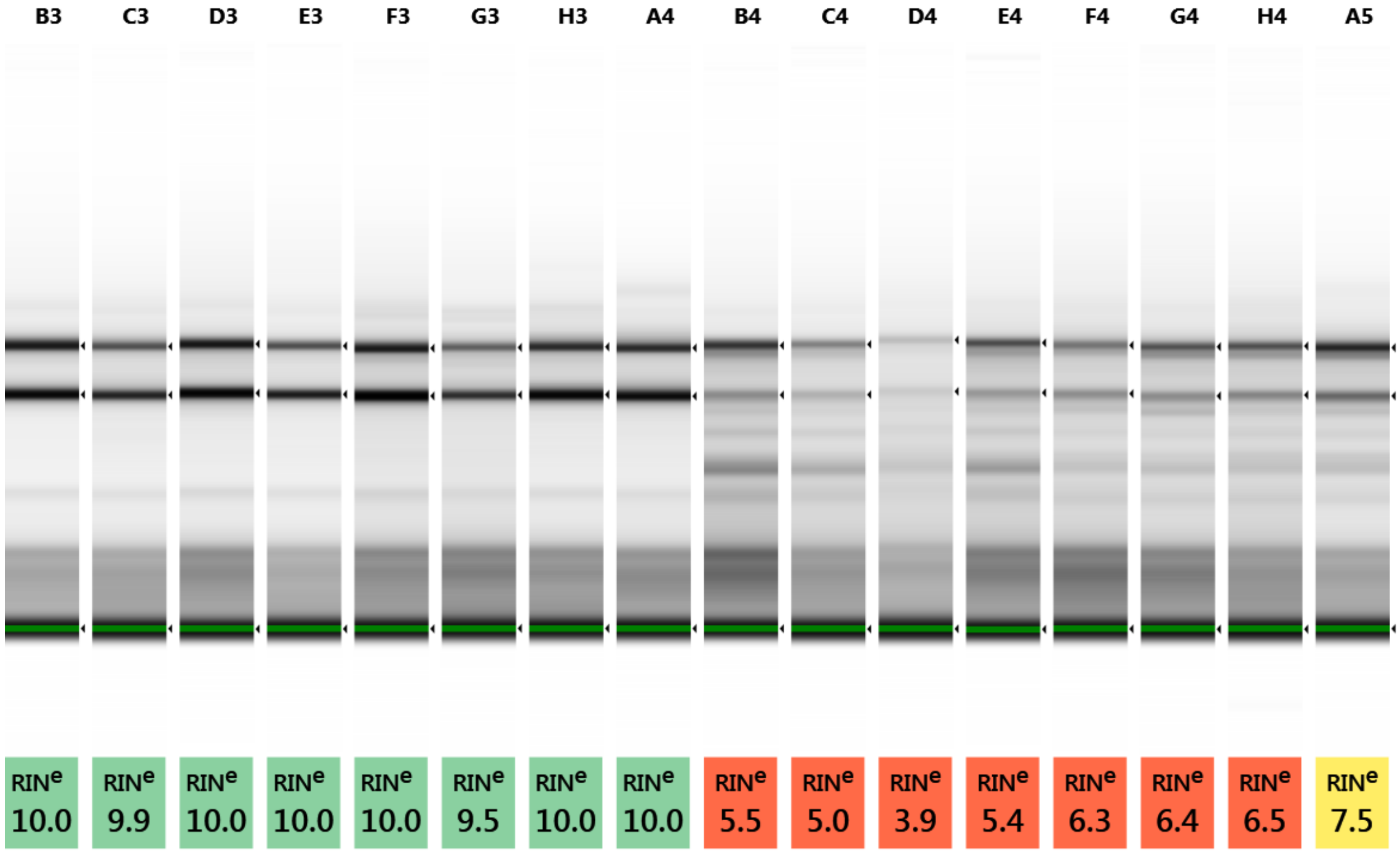

Default image (Contrast 100%)

 © Agilent Technologies, Inc. 2017

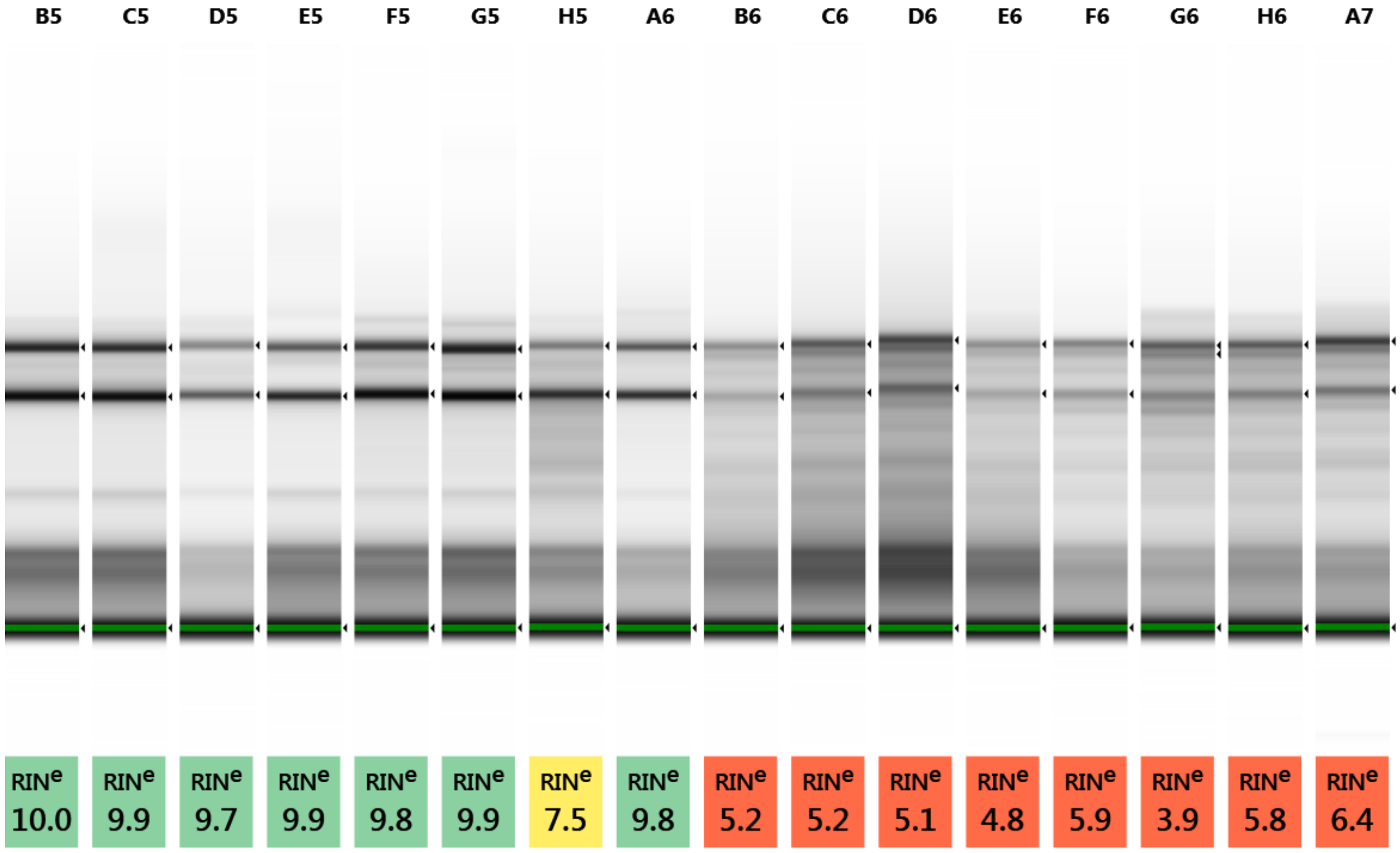

Default image (Contrast 100%)

Sample Info

Well	RINe	28S/18S (Area)	Conc. [ng/µl]	Sample Description	Alert	Observations
A1	-	-	84.9	Electronic Ladder		Ladder
B1	10.0	0.8	74.4	GK01-ASB-8-10-17-RNA-PA14-w-BIT-0-0A-3		
C1	9.4	2.7	129	GK02-ASB-8-10-17-RNA-PA14-w-BIT-0-0B-3		
D1	8.1	0.5	32.7	GK03-ASB-8-10-17-RNA-PA14-w-BIT-0-0C-3		
E1	7.6	1.6	76.1	GK04-ASB-8-10-17-RNA-PA14-w-BIT-0-0D-3		
F1	9.5	0.5	39.9	GK05-ASB-8-30-17-RNA-PA14-w-BIT-0-0A-2		
G1	8.5	0.6	33.8	GK06-ASB-8-30-17-RNA-PA14-w-BIT-0-0B-2		
H1	9.5	0.7	37.2	GK07-ASB-8-30-17-RNA-PA14-w-BIT-0-0C-2		
A2	9.7	0.7	73.3	GK08-ASB-8-30-17-RNA-PA14-w-BIT-0-0D-2		
B2	9.7	0.7	71.8	GK09-ASB-8-10-17-RNA-PA14-w-BIT-0-0-1A-3		
C2	9.0	0.7	36.9	GK10-ASB-8-10-17-RNA-PA14-w-BIT-0--0-1B-3		
D2	8.6	0.6	44.5	GK11-ASB-8-10-17-RNA-PA14-w-BIT-0-0-1C-3		
E2	9.1	2.2	27.1	GK12-ASB-8-10-17-RNA-PA14-w-BIT-0-0-1F-3		
F2	10.0	0.7	45.1	GK13-ASB-8-30-17-RNA-PA14-w-BIT-0--0-1A-2		
G2	10.0	0.7	47.2	GK14-ASB-8-30-17-RNA-PA14-w-BIT-0-0-1B-2		
H2	10.0	0.9	73.2	GK15-ASB-8-30-17-RNA-PA14-w-BIT-0-0-1D-2		
A3	10.0	0.8	54.1	GK16-ASB-8-30-17-RNA-PA14-w-BIT-0-0-1F-2		
B3	10.0	0.9	47.8	GK17-ASB-8-10-17-RNA-PA14-w-BIT-5-0A-3		
C3	9.9	0.7	39.6	GK18-ASB-8-10-17-RNA-PA14-w-BIT-5-0B-3		
D3	10.0	0.8	54.4	GK19-ASB-8-10-17-RNA-PA14-w-BIT-5-0C-3		
E3	10.0	0.7	37.4	GK20-ASB-8-10-17-RNA-PA14-w-BIT-5-0D-3		

 © Agilent Technologies, Inc. 2017

F3	10.0	0.7	58.4	GK21-ASB-8-30-17-RNA-PA14-w-BIT-5-0A-2		
G3	9.5	0.7	44.3	GK22-ASB-8-30-17-RNA-PA14-w-BIT-5-0B-2		
H3	10.0	0.7	50.2	GK23-ASB-8-30-17-RNA-PA14-w-BIT-5-0C-2		
A4	10.0	0.7	50.2	GK24-ASB-8-30-17-RNA-PA14-w-BIT-5-0D-2		
B4	5.5	1.4	66.7	GK25-ASB-8-10-17-RNA-PA14-w-BIT-5-0-1A-3		
C4	5.0	1.0	41.5	GK26-ASB-8-10-17-RNA-PA14-w-BIT-5--0-1B-3		
D4	3.9	1.2	31.5	GK27-ASB-8-10-17-RNA-PA14-w-BIT-5-0-1C-3		
E4	5.4	1.2	56.3	GK28-ASB-8-10-17-RNA-PA14-w-BIT-5-0-1F-3		
F4	6.3	1.4	51.5	GK29-ASB-8-30-17-RNA-PA14-w-BIT-5-0-1A-2		
G4	6.4	1.2	51.0	GK30-ASB-8-30-17-RNA-PA14-w-BIT-5--0-1B-2		
H4	6.5	1.1	46.7	GK31-ASB-8-30-17-RNA-PA14-w-BIT-5-0-1D-2		
A5	7.5	1.3	46.3	GK32-ASB-8-30-17-RNA-PA14-w-BIT-5-0-1F-2		
B5	10.0	0.7	57.7	GK33-ASB-8-10-17-RNA-PA14-w-BIT-24-0A-3		
C5	9.9	0.7	59.3	GK34-ASB-8-10-17-RNA-PA14-w-BIT-24-0B-3		
D5	9.7	0.7	26.6	GK35-ASB-8-10-17-RNA-PA14-w-BIT-24-0C-3		
E5	9.9	0.6	43.6	GK36-ASB-8-10-17-RNA-PA14-w-BIT-24-0D-3		
F5	9.8	0.6	54.6	GK37-ASB-8-30-17-RNA-PA14-w-BIT-24-0A-2		
G5	9.9	0.8	61.2	GK38-ASB-8-30-17-RNA-PA14-w-BIT-24-0B-2		
H5	7.5	0.4	51.7	GK39-ASB-8-30-17-RNA-PA14-w-BIT-24-0C-2		
A6	9.8	0.7	33.1	GK40-ASB-8-30-17-RNA-PA14-w-BIT-24-0D-2		
B6	5.2	0.9	49.4	GK41-ASB-8-10-17-RNA-PA14-w-BIT-24-0-1A-3		
C6	5.2	0.8	79.7	GK42-ASB-8-10-17-RNA-PA14-w-BIT-24--0-1B-3		
D6	5.1	0.9	95.2	GK43-ASB-8-10-17-RNA-PA14-w-BIT-24-0-1C-3		
E6	4.8	1.1	54.1	GK44-ASB-8-10-17-RNA-PA14-w-BIT-24-0-1F-3		
F6	5.9	0.8	35.6	GK45-ASB-8-30-17-RNA-PA14-w-BIT-24-0-1A-2		
G6	3.9	1.5	48.2	GK46-ASB-8-30-17-RNA-PA14-w-BIT-24-0-1B-2		
H6	5.8	1.0	47.7	GK47-ASB-8-30-17-RNA-PA14-w-BIT-24-0-1D-2		
A7	6.4	1.1	48.9	GK48-ASB-8-30-17-RNA-PA14-w-BIT-24-0-1F-2		

A1: Electronic Ladder

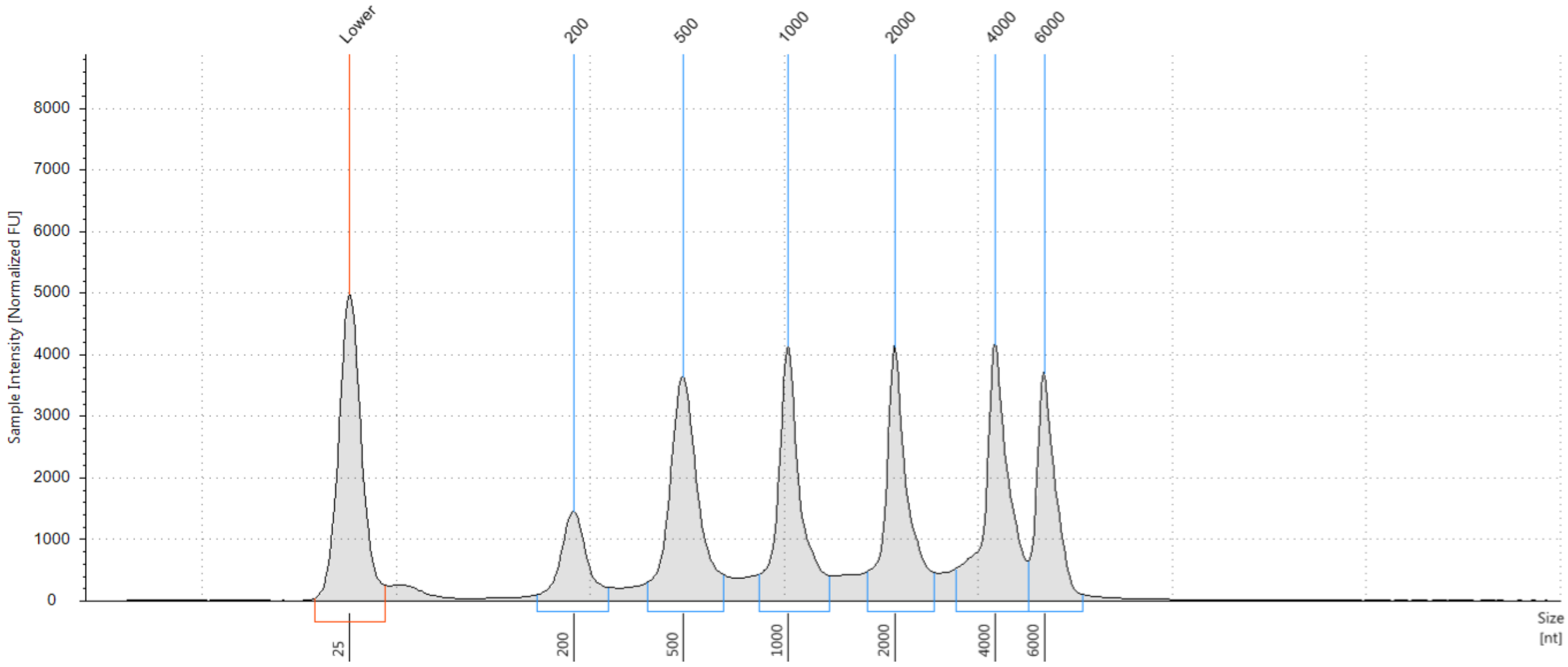

Sample Table

Well	RINe	28S/18S (Area)	Conc. [ng/µl]	Sample Description	Alert	Observations
A1	-	-	84.9	Electronic Ladder		Ladder

Peak Table

Size [nt]	Calibrated Conc. [ng/µl]	Assigned Conc. [ng/µl]	Peak Molarity [nmol/l]	% Integrated Area	Peak Comment	Observations
25	40.0	40.0	4710	-		Lower Marker
200	5.94	-	87.4	7.80		
500	15.9	-	93.6	20.88		
1000	14.2	-	41.8	18.62		
2000	13.8	-	20.3	18.11		
4000	15.5	-	11.4	20.38		
6000	10.8	-	5.31	14.22		

 © Agilent Technologies, Inc. 2017

B1: GK01-ASB-8-10-17-RNA-PA14-w-BIT-0-0A-3

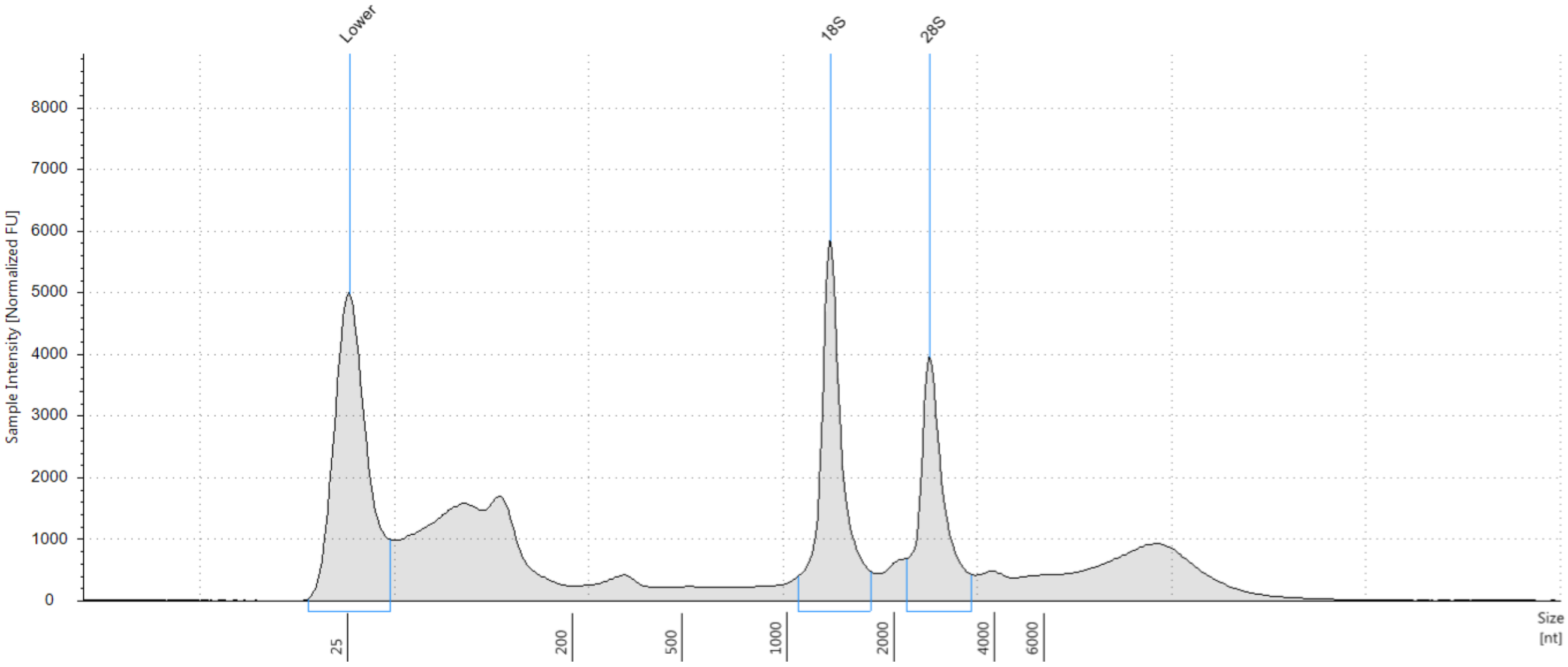

Sample Table

Well	RINe	28S/18S (Area)	Conc. [ng/µl]	Sample Description	Alert	Observations
B1	10.0	0.8	74.4	GK01-ASB-8-10-17-RNA-PA14-w-BIT-0-0A-3		

Peak Table

Size [nt]	Calibrated Conc. [ng/µl]	Assigned Conc. [ng/µl]	Peak Molarity [nmol/l]	% Integrated Area	Peak Comment	Observations
25	40.0	40.0	4710	-		Lower Marker
1312	15.8	-	35.3	57.13		18S
2546	11.8	-	13.7	42.87		28S

C1: GK02-ASB-8-10-17-RNA-PA14-w-BIT-0-0B-3

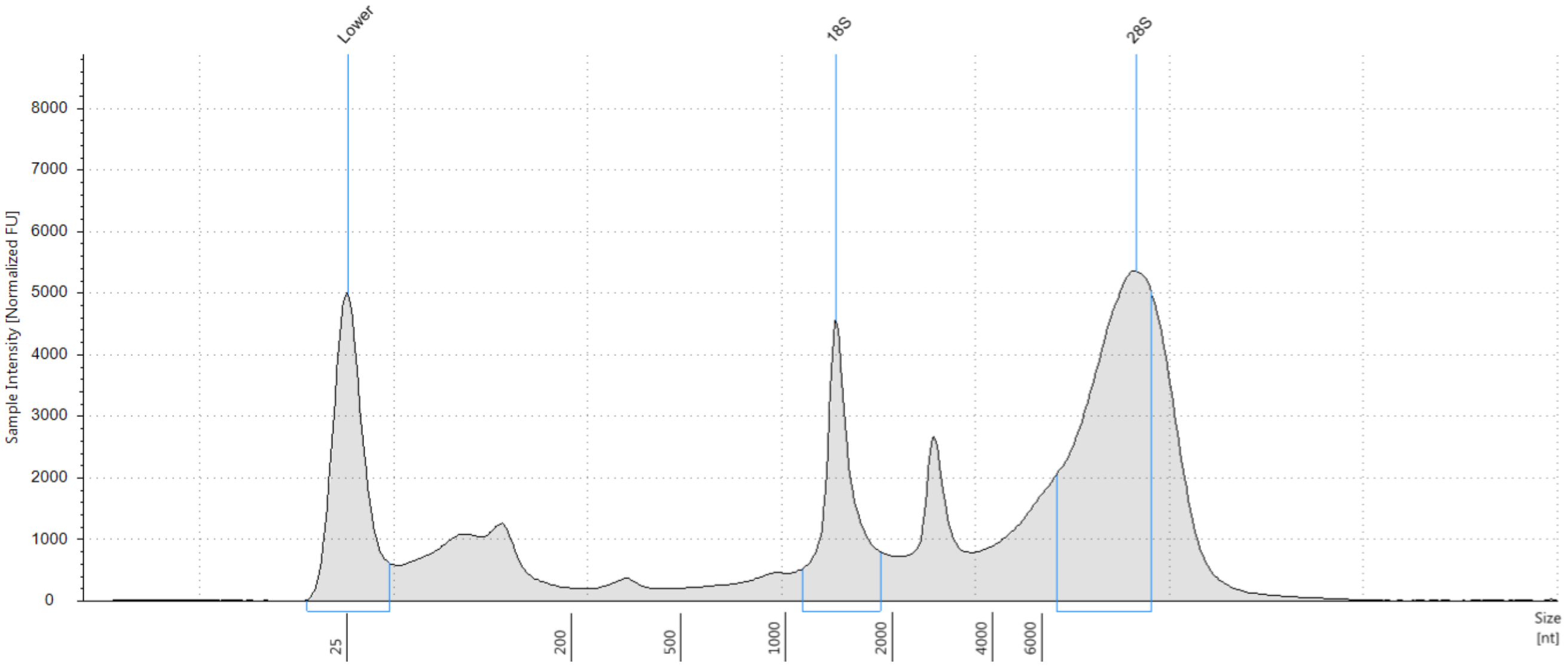

Sample Table

Well	RINe	28S/18S (Area)	Conc. [ng/µl]	Sample Description	Alert	Observations
C1	9.4	2.7	129	GK02-ASB-8-10-17-RNA-PA14-w-BIT-0-0B-3		

Peak Table

Size [nt]	Calibrated Conc. [ng/µl]	Assigned Conc. [ng/µl]	Peak Molarity [nmol/l]	% Integrated Area	Peak Comment	Observations
25	40.0	40.0	4710	-		Lower Marker
1381	16.5	-	35.1	26.73		18S
13135	45.2	-	10.1	73.27		28S

 © Agilent Technologies, Inc. 2017

D1: GK03-ASB-8-10-17-RNA-PA14-w-BIT-0-0C-3

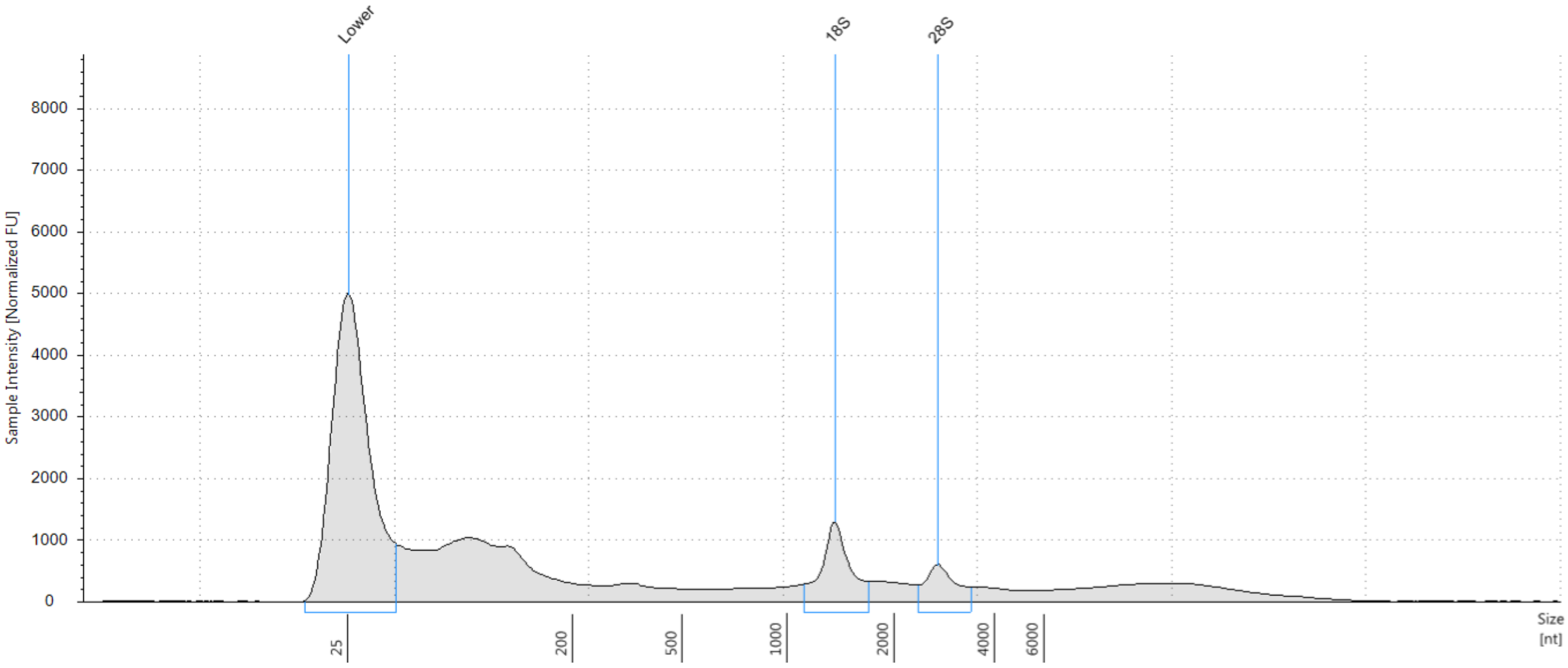

Sample Table

Well	RINe	28S/18S (Area)	Conc. [ng/µl]	Sample Description	Alert	Observations
D1	8.1	0.5	32.7	GK03-ASB-8-10-17-RNA-PA14-w-BIT-0-0C-3		

Peak Table

Size [nt]	Calibrated Conc. [ng/µl]	Assigned Conc. [ng/µl]	Peak Molarity [nmol/l]	% Integrated Area	Peak Comment	Observations
25	40.0	40.0	4710	-		Lower Marker
1360	3.97	-	8.59	66.78		18S
2702	1.97	-	2.15	33.22		28S

 © Agilent Technologies, Inc. 2017

E1: GK04-ASB-8-10-17-RNA-PA14-w-BIT-0-0D-3

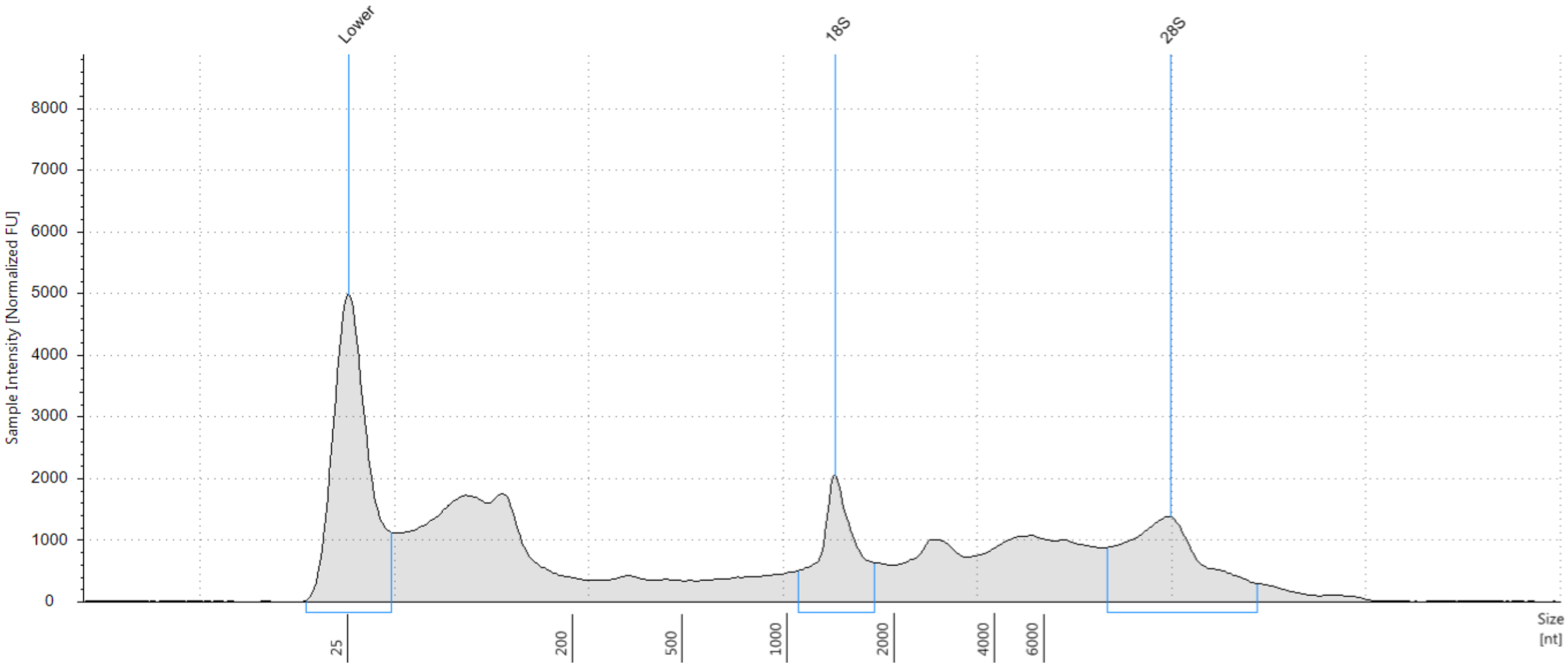

Sample Table

Well	RINe	28S/18S (Area)	Conc. [ng/µl]	Sample Description	Alert	Observations
E1	7.6	1.6	76.1	GK04-ASB-8-10-17-RNA-PA14-w-BIT-0-0D-3		

Peak Table

Size [nt]	Calibrated Conc. [ng/µl]	Assigned Conc. [ng/µl]	Peak Molarity [nmol/l]	% Integrated Area	Peak Comment	Observations
25	40.0	40.0	4710	-		Lower Marker
1360	8.32	-	18.0	38.42		18S
17091	13.3	-	2.30	61.58		28S

F1: GK05-ASB-8-30-17-RNA-PA14-w-BIT-0-0A-2

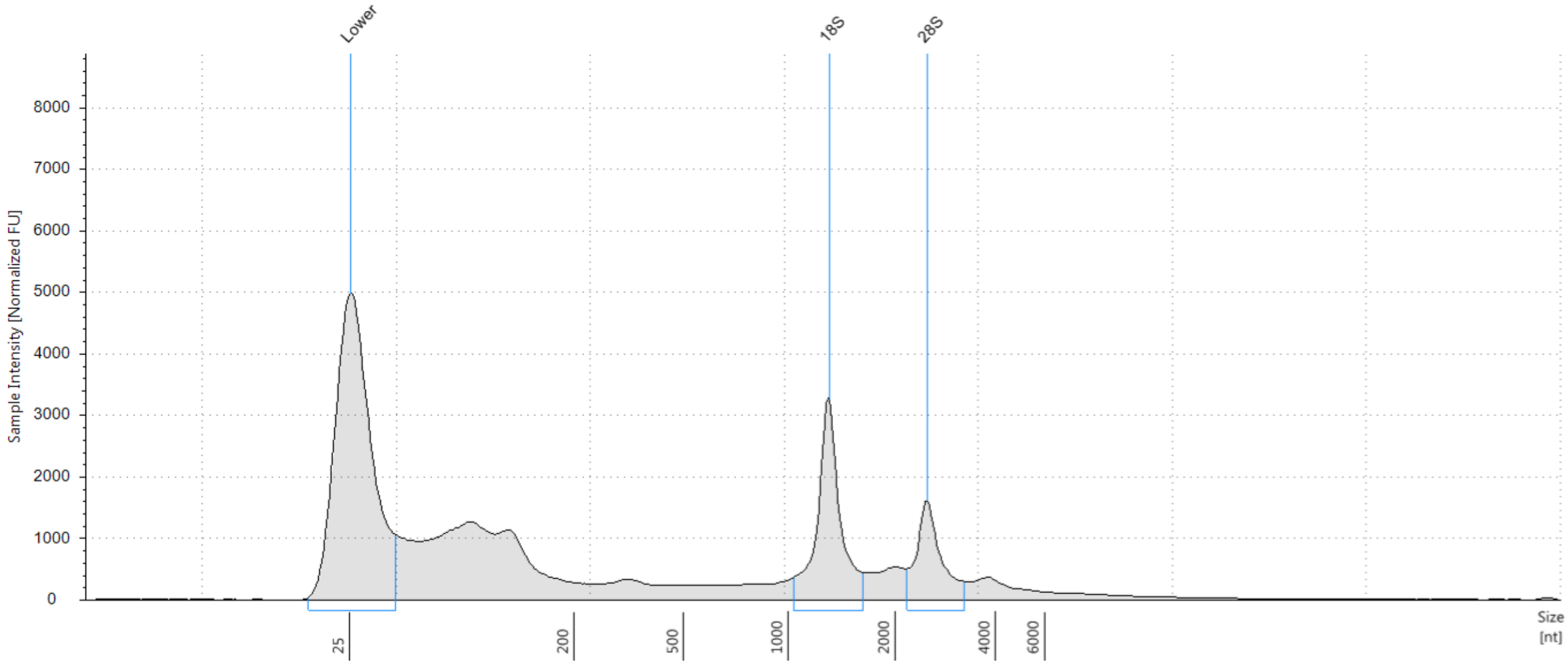

Sample Table

Well	RINe	28S/18S (Area)	Conc. [ng/µl]	Sample Description	Alert	Observations
F1	9.5	0.5	39.9	GK05-ASB-8-30-17-RNA-PA14-w-BIT-0-0A-2		

Peak Table

Size [nt]	Calibrated Conc. [ng/µl]	Assigned Conc. [ng/µl]	Peak Molarity [nmol/l]	% Integrated Area	Peak Comment	Observations
25	40.0	40.0	4710	-		Lower Marker
1296	8.54	-	19.4	65.31		18S
2485	4.53	-	5.37	34.69		28S

G1: GK06-ASB-8-30-17-RNA-PA14-w-BIT-0-0B-2

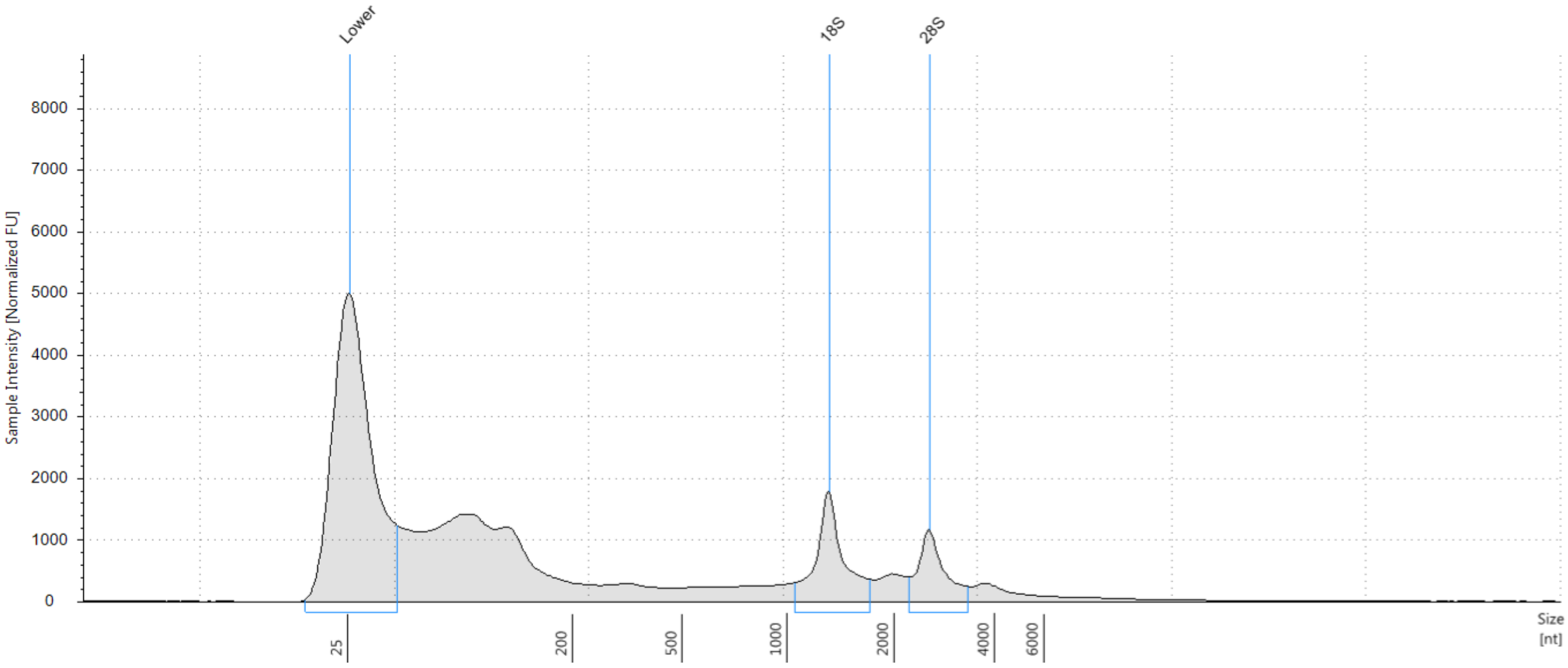

Sample Table

Well	RINe	28S/18S (Area)	Conc. [ng/µl]	Sample Description	Alert	Observations
G1	8.5	0.6	33.8	GK06-ASB-8-30-17-RNA-PA14-w-BIT-0-0B-2		

Peak Table

Size [nt]	Calibrated Conc. [ng/µl]	Assigned Conc. [ng/µl]	Peak Molarity [nmol/l]	% Integrated Area	Peak Comment	Observations
25	40.0	40.0	4710	-		Lower Marker
1308	5.13	-	11.5	61.11		18S
2535	3.27	-	3.79	38.89		28S

H1: GK07-ASB-8-30-17-RNA-PA14-w-BIT-0-0C-2

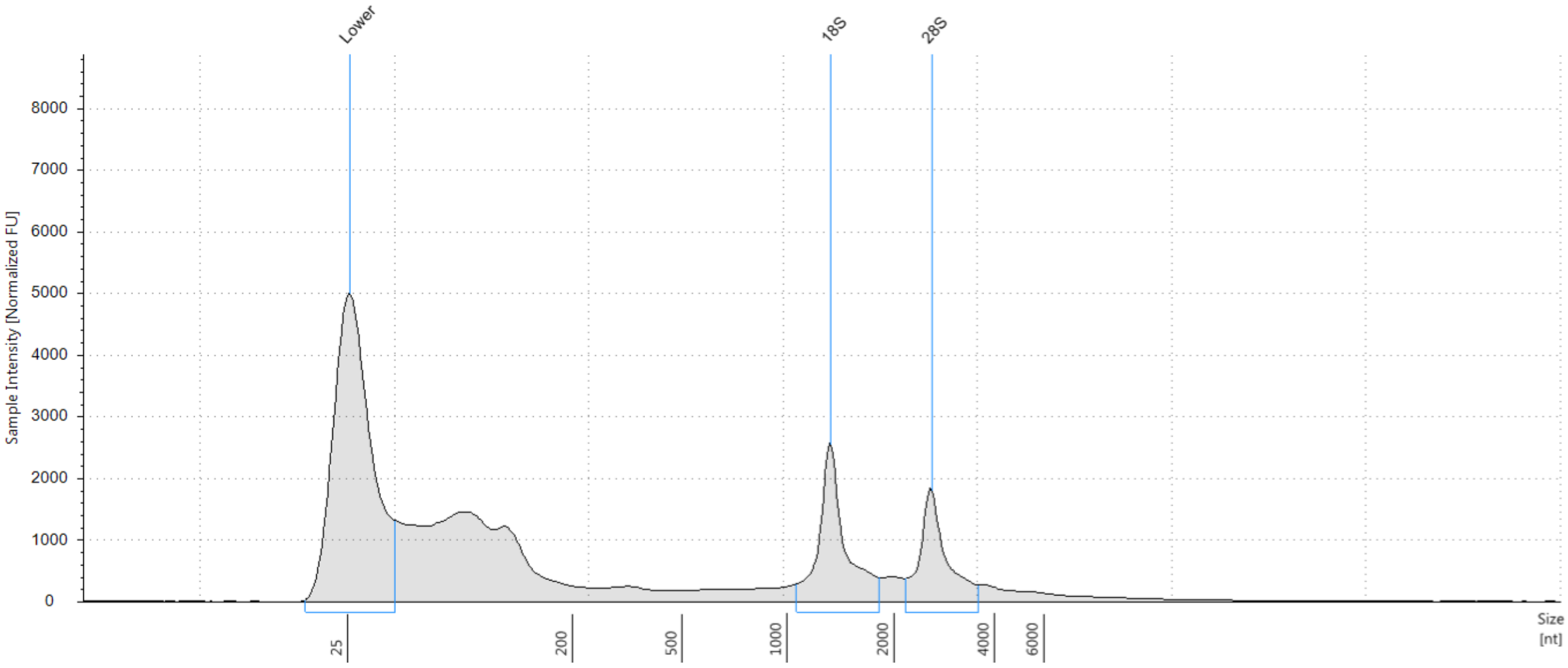

Sample Table

Well	RINe	28S/18S (Area)	Conc. [ng/µl]	Sample Description	Alert	Observations
H1	9.5	0.7	37.2	GK07-ASB-8-30-17-RNA-PA14-w-BIT-0-0C-2		

Peak Table

Size [nt]	Calibrated Conc. [ng/µl]	Assigned Conc. [ng/µl]	Peak Molarity [nmol/l]	% Integrated Area	Peak Comment	Observations
25	40.0	40.0	4710	-		Lower Marker
1318	7.18	-	16.0	58.06		18S
2568	5.19	-	5.94	41.94		28S

 © Agilent Technologies, Inc. 2017

A2: GK08-ASB-8-30-17-RNA-PA14-w-BIT-0-0D-2

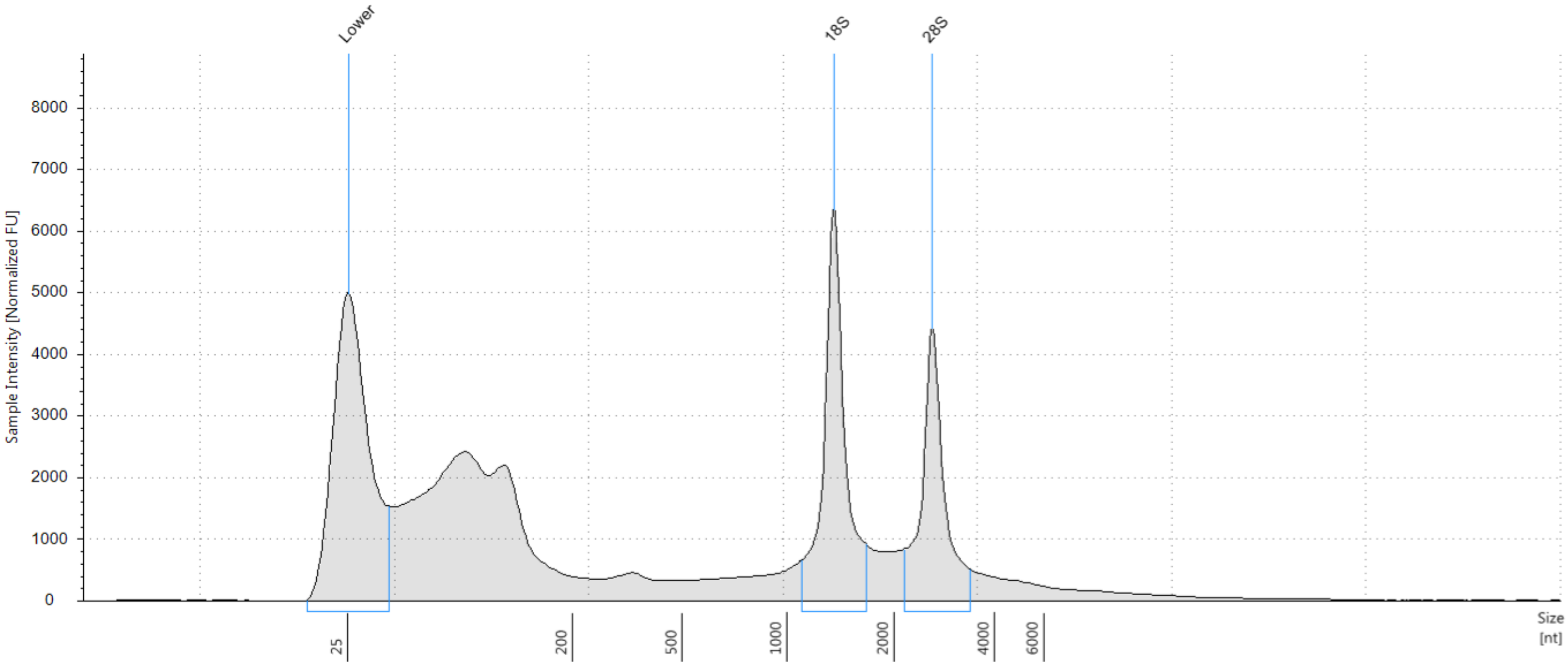

Sample Table

Well	RINe	28S/18S (Area)	Conc. [ng/µl]	Sample Description	Alert	Observations
A2	9.7	0.7	73.3	GK08-ASB-8-30-17-RNA-PA14-w-BIT-0-0D-2		

Peak Table

Size [nt]	Calibrated Conc. [ng/µl]	Assigned Conc. [ng/µl]	Peak Molarity [nmol/l]	% Integrated Area	Peak Comment	Observations
25	40.0	40.0	4710	-		Lower Marker
1349	15.4	-	33.6	57.36		18S
2590	11.5	-	13.0	42.64		28S

 © Agilent Technologies, Inc. 2017

B2: GK09-ASB-8-10-17-RNA-PA14-w-BIT-0-0-1A-3

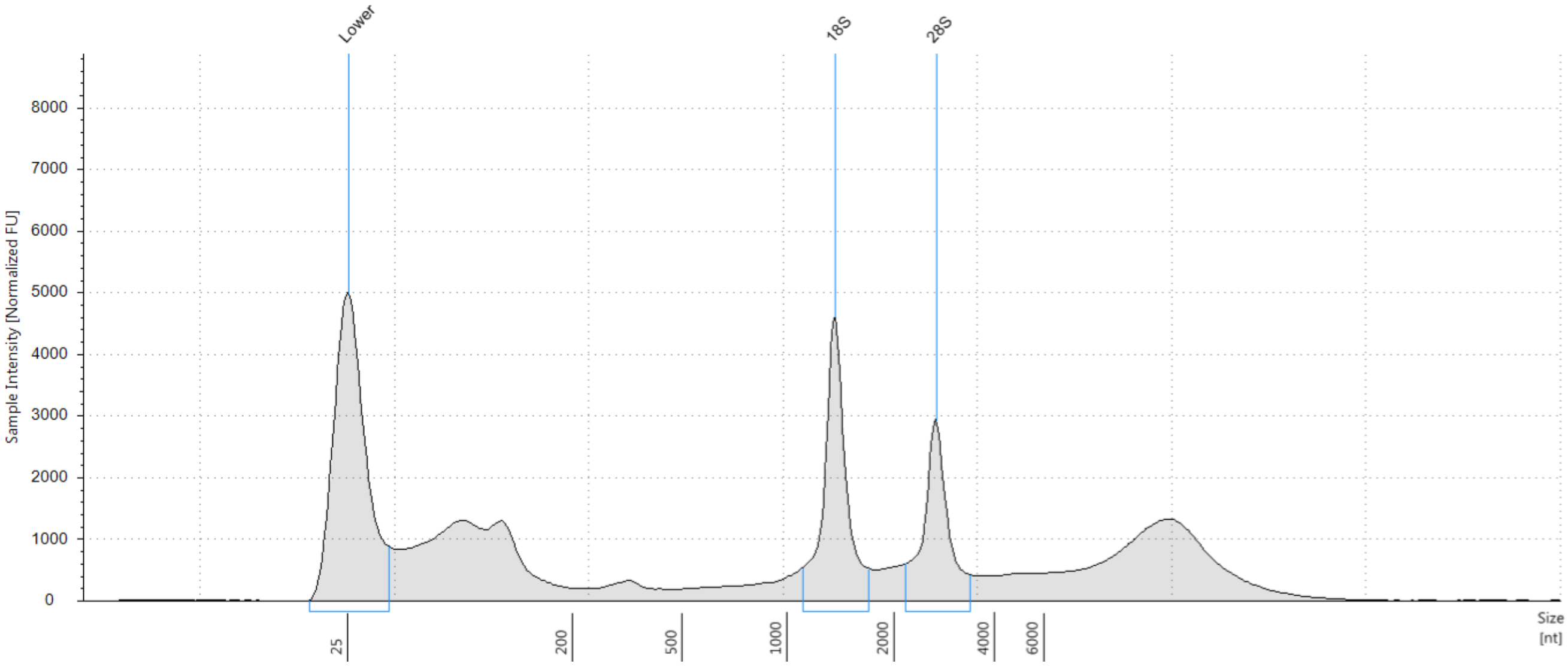

Sample Table

Well	RINe	28S/18S (Area)	Conc. [ng/µl]	Sample Description	Alert	Observations
B2	9.7	0.7	71.8	GK09-ASB-8-10-17-RNA-PA14-w-BIT-0-0-1A-3		

Peak Table

Size [nt]	Calibrated Conc. [ng/µl]	Assigned Conc. [ng/µl]	Peak Molarity [nmol/l]	% Integrated Area	Peak Comment	Observations
25	40.0	40.0	4710	-		Lower Marker
1360	13.1	-	28.2	58.59		18S
2663	9.22	-	10.2	41.41		28S

 © Agilent Technologies, Inc. 2017

C2: GK10-ASB-8-10-17-RNA-PA14-w-BIT-0--0-1B-3

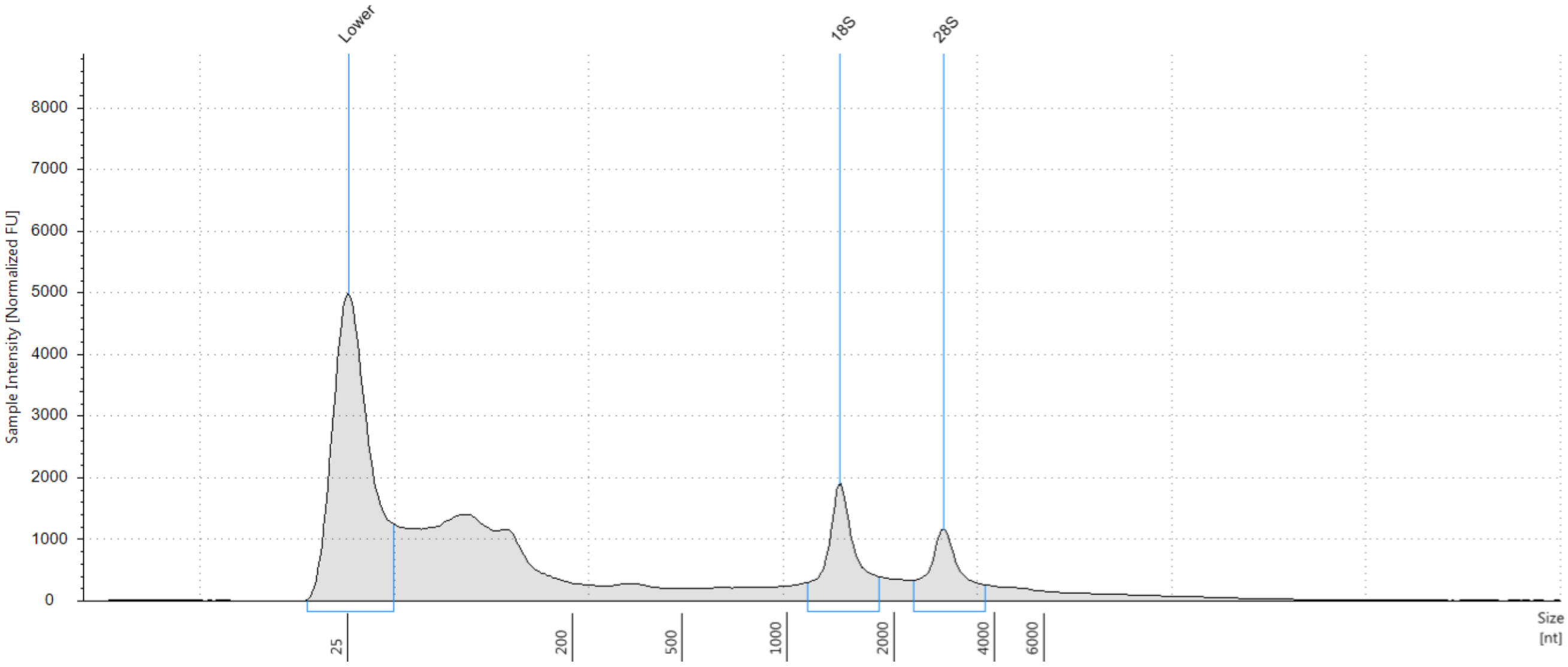

Sample Table

Well	RINe	28S/18S (Area)	Conc. [ng/µl]	Sample Description	Alert	Observations
C2	9.0	0.7	36.9	GK10-ASB-8-10-17-RNA-PA14-w-BIT-0--0-1B-3		

Peak Table

Size [nt]	Calibrated Conc. [ng/µl]	Assigned Conc. [ng/µl]	Peak Molarity [nmol/l]	% Integrated Area	Peak Comment	Observations
25	40.0	40.0	4710	-		Lower Marker
1402	5.88	-	12.3	59.21		18S
2785	4.05	-	4.28	40.79		28S

© Agilent Technologies, Inc. 2017

D2: GK11-ASB-8-10-17-RNA-PA14-w-BIT-0-0-1C-3

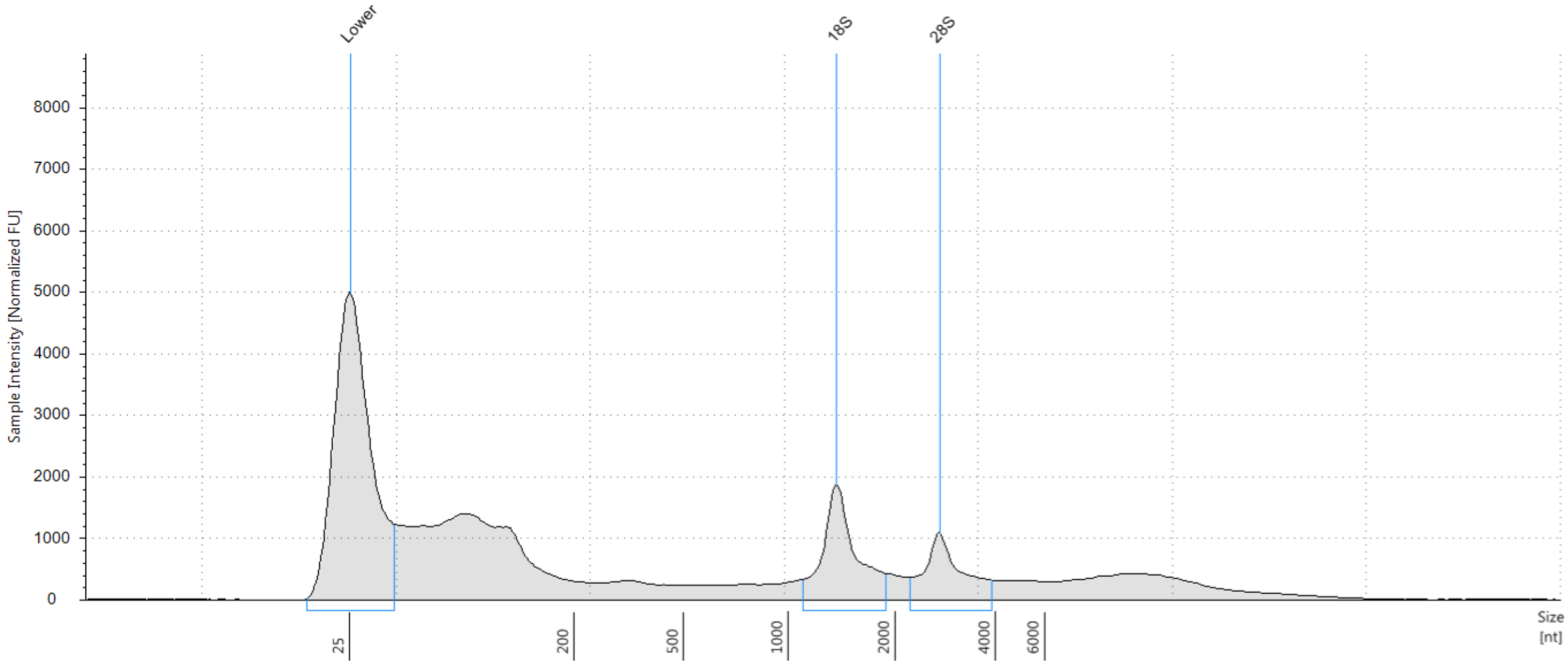

Sample Table

Well	RINe	28S/18S (Area)	Conc. [ng/µl]	Sample Description	Alert	Observations
D2	8.6	0.6	44.5	GK11-ASB-8-10-17-RNA-PA14-w-BIT-0-0-1C-3		

Peak Table

Size [nt]	Calibrated Conc. [ng/µl]	Assigned Conc. [ng/µl]	Peak Molarity [nmol/l]	% Integrated Area	Peak Comment	Observations
25	40.0	40.0	4710	-		Lower Marker
1364	6.89	-	14.9	60.89		18S
2711	4.43	-	4.80	39.11		28S

 © Agilent Technologies, Inc. 2017

E2: GK12-ASB-8-10-17-RNA-PA14-w-BIT-0-0-1F-3

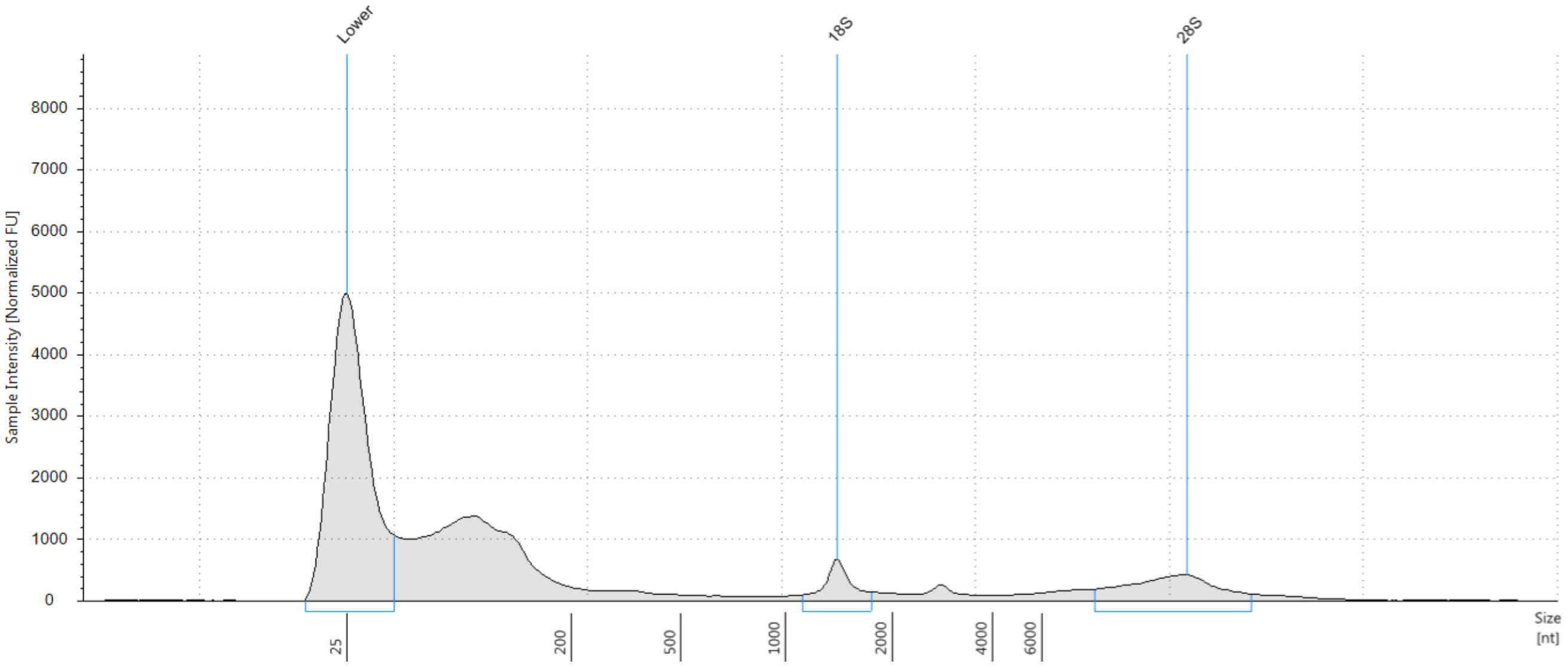

Sample Table

Well	RINe	28S/18S (Area)	Conc. [ng/µl]	Sample Description	Alert	Observations
E2	9.1	2.2	27.1	GK12-ASB-8-10-17-RNA-PA14-w-BIT-0-0-1F-3		

Peak Table

Size [nt]	Calibrated Conc. [ng/µl]	Assigned Conc. [ng/µl]	Peak Molarity [nmol/l]	% Integrated Area	Peak Comment	Observations
25	40.0	40.0	4710	-		Lower Marker
1399	1.84	-	3.87	31.51		18S
19993	4.00	-	0.588	68.49		28S

© Agilent Technologies, Inc. 2017

F2: GK13-ASB-8-30-17-RNA-PA14-w-BIT-0--0-1A-2

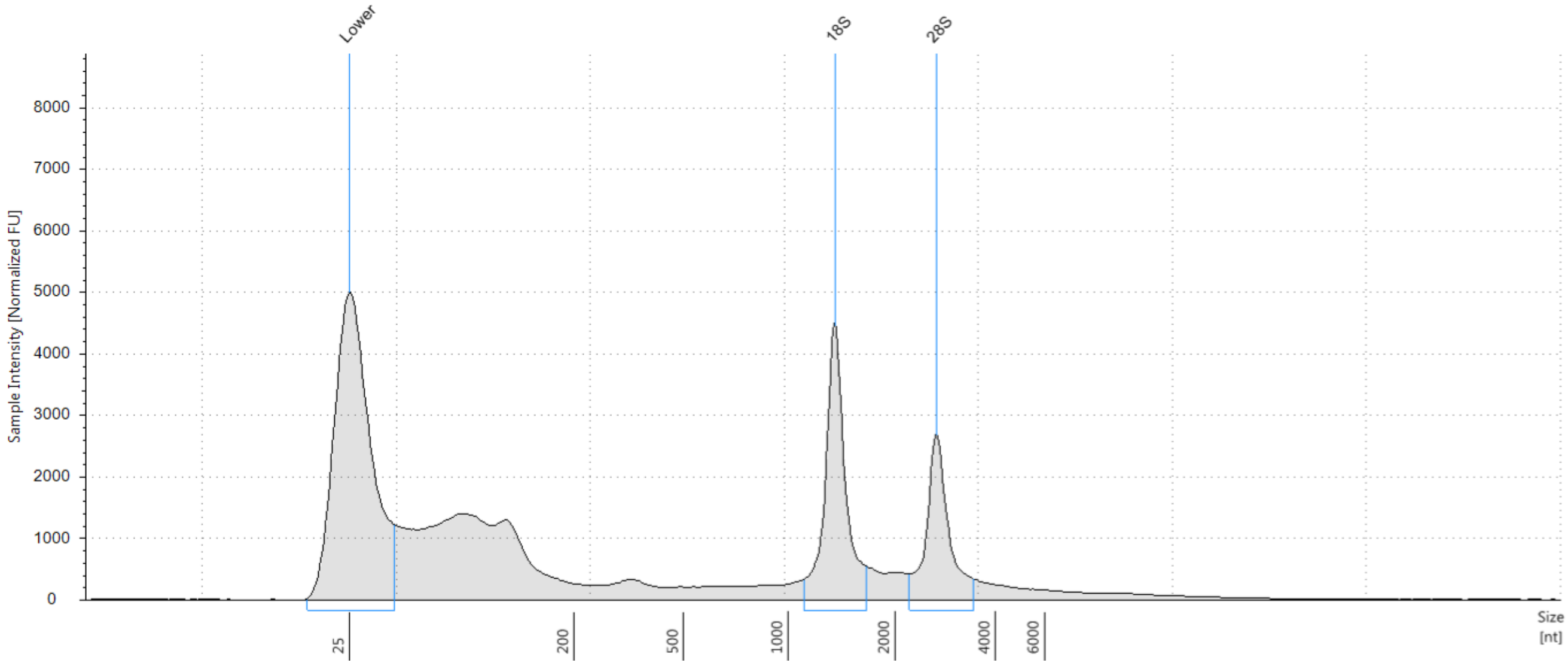

Sample Table

Well	RINe	28S/18S (Area)	Conc. [ng/µl]	Sample Description	Alert	Observations
F2	10.0	0.7	45.1	GK13-ASB-8-30-17-RNA-PA14-w-BIT-0--0-1A-2		

Peak Table

Size [nt]	Calibrated Conc. [ng/µl]	Assigned Conc. [ng/µl]	Peak Molarity [nmol/l]	% Integrated Area	Peak Comment	Observations
25	40.0	40.0	4710	-		Lower Marker
1359	10.2	-	22.0	60.02		18S
2666	6.78	-	7.49	39.98		28S

 © Agilent Technologies, Inc. 2017

G2: GK14-ASB-8-30-17-RNA-PA14-w-BIT-0-0-1B-2

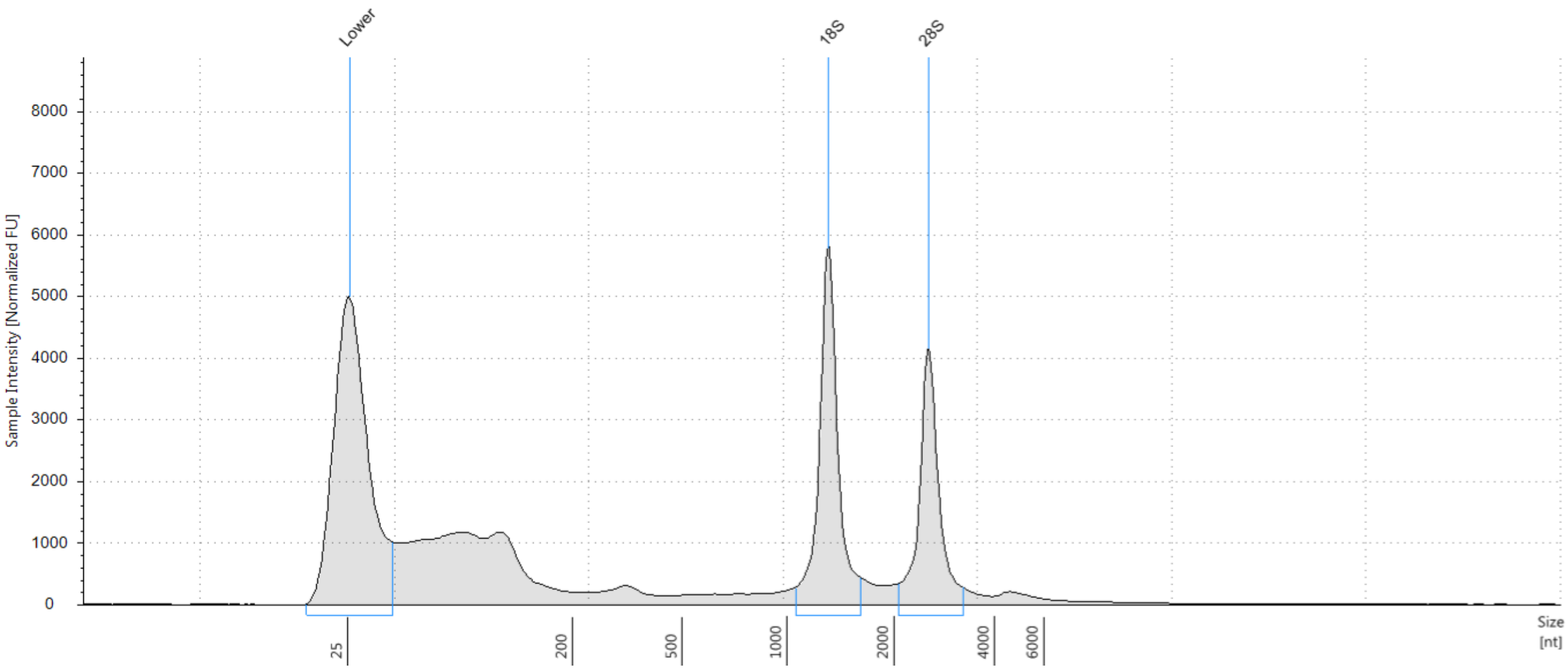

Sample Table

Well	RINe	28S/18S (Area)	Conc. [ng/µl]	Sample Description	Alert	Observations
G2	10.0	0.7	47.2	GK14-ASB-8-30-17-RNA-PA14-w-BIT-0-0-1B-2		

Peak Table

Size [nt]	Calibrated Conc. [ng/µl]	Assigned Conc. [ng/µl]	Peak Molarity [nmol/l]	% Integrated Area	Peak Comment	Observations
25	40.0	40.0	4710	-		Lower Marker
1304	13.2	-	29.8	57.24		18S
2524	9.87	-	11.5	42.76		28S

 © Agilent Technologies, Inc. 2017

H2: GK15-ASB-8-30-17-RNA-PA14-w-BIT-0-0-1D-2

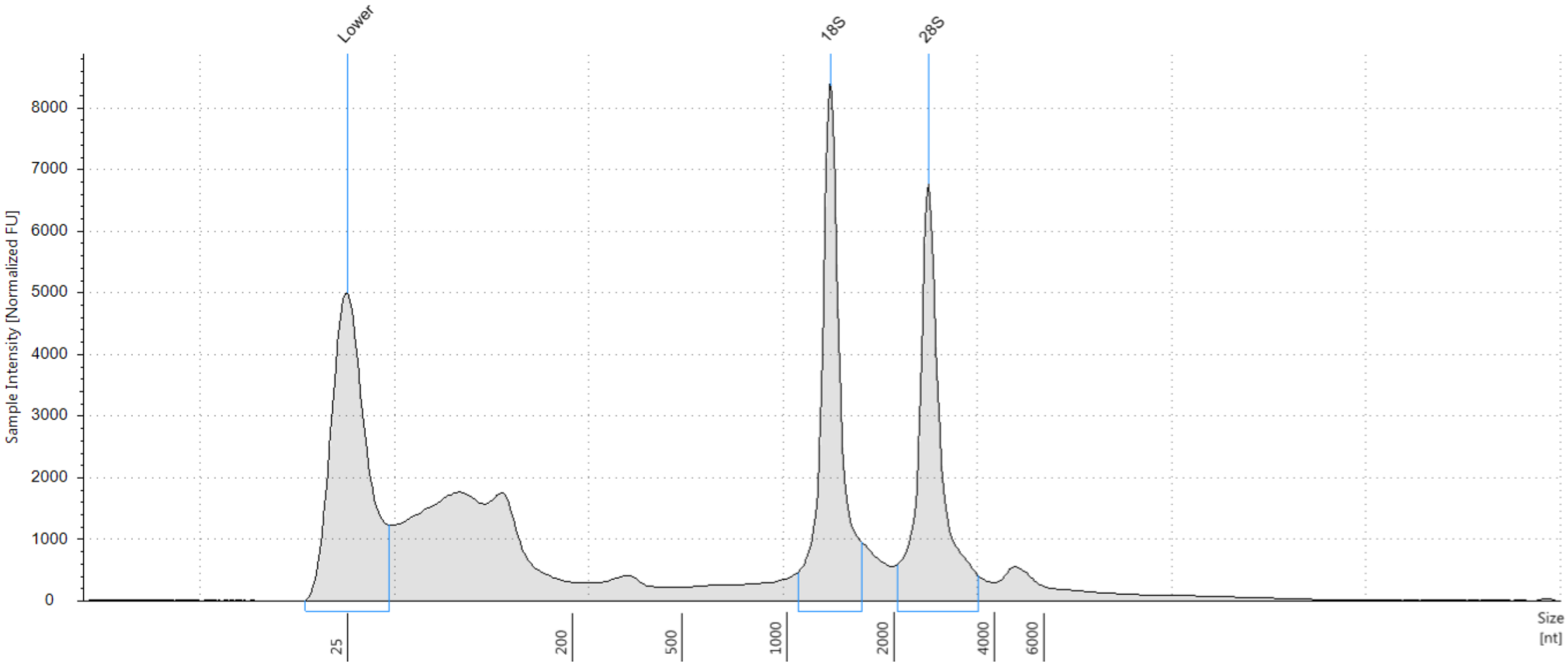

Sample Table

Well	RINe	28S/18S (Area)	Conc. [ng/µl]	Sample Description	Alert	Observations
H2	10.0	0.9	73.2	GK15-ASB-8-30-17-RNA-PA14-w-BIT-0-0-1D-2		

Peak Table

Size [nt]	Calibrated Conc. [ng/µl]	Assigned Conc. [ng/µl]	Peak Molarity [nmol/l]	% Integrated Area	Peak Comment	Observations
25	40.0	40.0	4710	-		Lower Marker
1323	18.6	-	41.5	52.04		18S
2542	17.2	-	19.9	47.96		28S

A3: GK16-ASB-8-30-17-RNA-PA14-w-BIT-0-0-1F-2

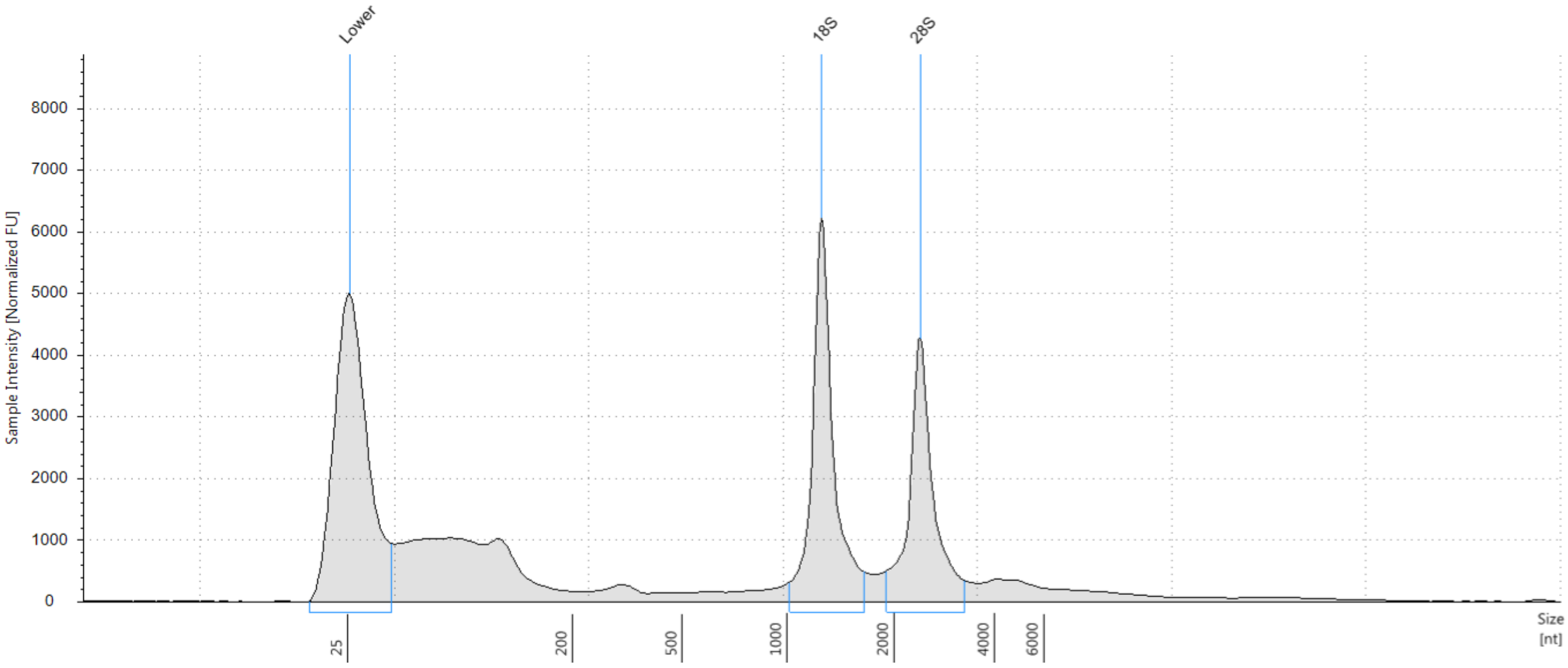

Sample Table

Well	RINe	28S/18S (Area)	Conc. [ng/µl]	Sample Description	Alert	Observations
A3	10.0	0.8	54.1	GK16-ASB-8-30-17-RNA-PA14-w-BIT-0-0-1F-2		

Peak Table

Size [nt]	Calibrated Conc. [ng/µl]	Assigned Conc. [ng/µl]	Peak Molarity [nmol/l]	% Integrated Area	Peak Comment	Observations
25	40.0	40.0	4710	-		Lower Marker
1242	15.9	-	37.7	56.02		18S
2368	12.5	-	15.5	43.98		28S

B3: GK17-ASB-8-10-17-RNA-PA14-w-BIT-5-0A-3

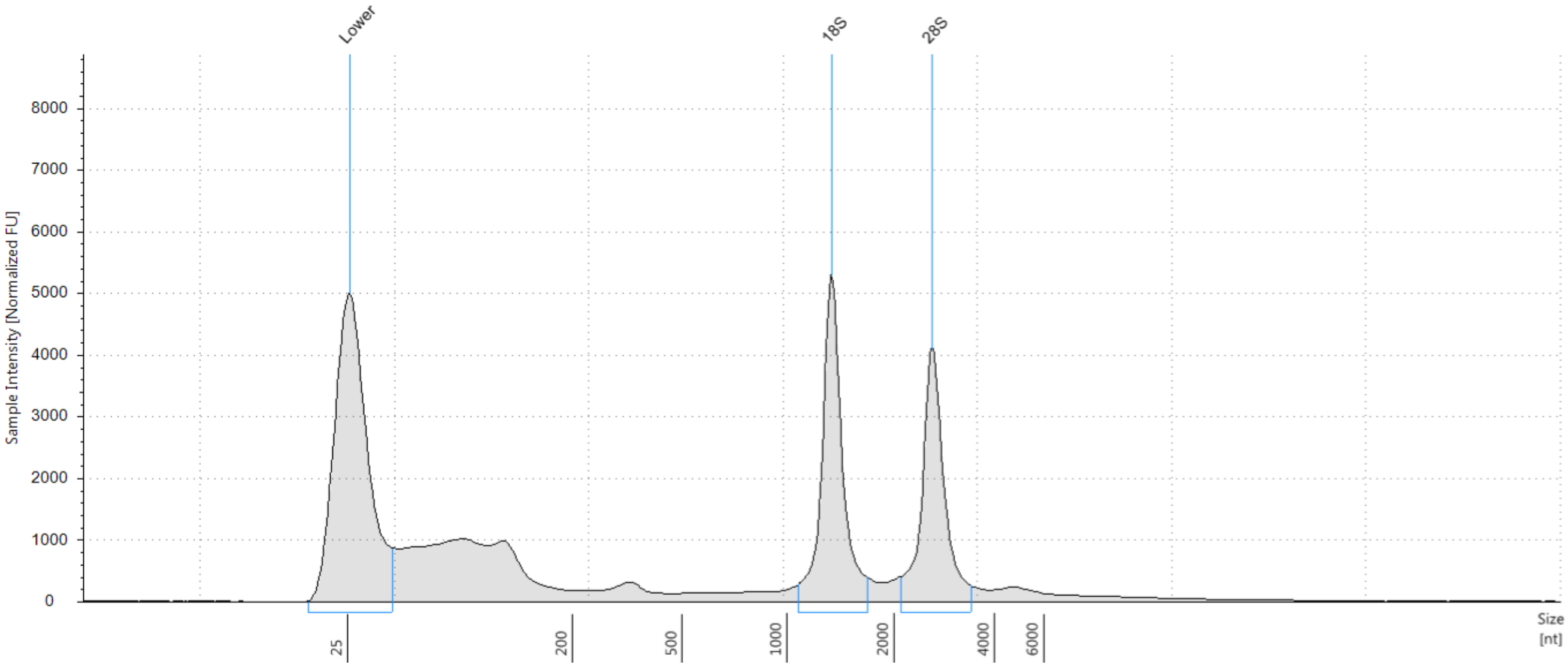

Sample Table

Well	RINe	28S/18S (Area)	Conc. [ng/µl]	Sample Description	Alert	Observations
B3	10.0	0.9	47.8	GK17-ASB-8-10-17-RNA-PA14-w-BIT-5-0A-3		

Peak Table

Size [nt]	Calibrated Conc. [ng/µl]	Assigned Conc. [ng/µl]	Peak Molarity [nmol/l]	% Integrated Area	Peak Comment	Observations
25	40.0	40.0	4710	-		Lower Marker
1325	13.4	-	29.7	54.04		18S
2582	11.4	-	13.0	45.96		28S

 © Agilent Technologies, Inc. 2017

C3: GK18-ASB-8-10-17-RNA-PA14-w-BIT-5-0B-3

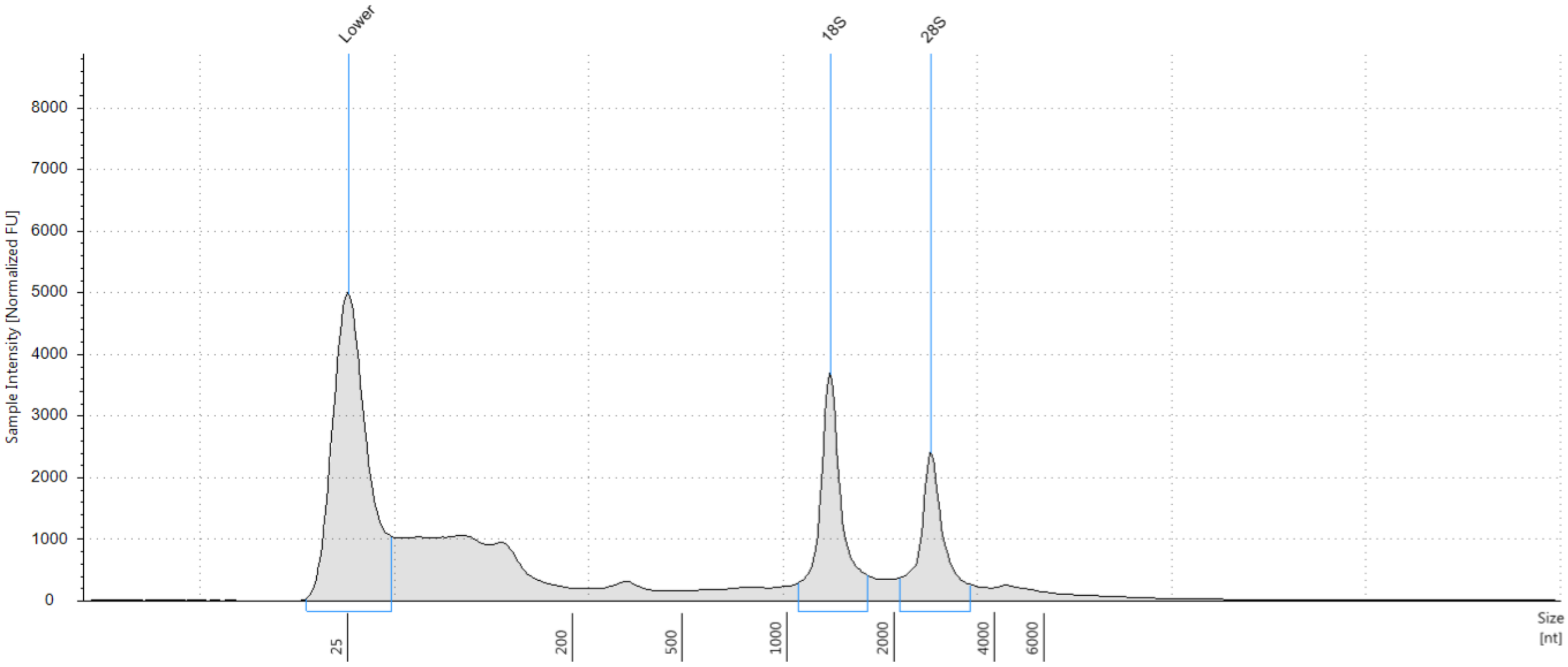

Sample Table

Well	RINe	28S/18S (Area)	Conc. [ng/µl]	Sample Description	Alert	Observations
C3	9.9	0.7	39.6	GK18-ASB-8-10-17-RNA-PA14-w-BIT-5-0B-3		

Peak Table

Size [nt]	Calibrated Conc. [ng/µl]	Assigned Conc. [ng/µl]	Peak Molarity [nmol/l]	% Integrated Area	Peak Comment	Observations
25	40.0	40.0	4710	-		Lower Marker
1319	9.48	-	21.1	58.30		18S
2556	6.78	-	7.80	41.70		28S

 © Agilent Technologies, Inc. 2017

D3: GK19-ASB-8-10-17-RNA-PA14-w-BIT-5-0C-3

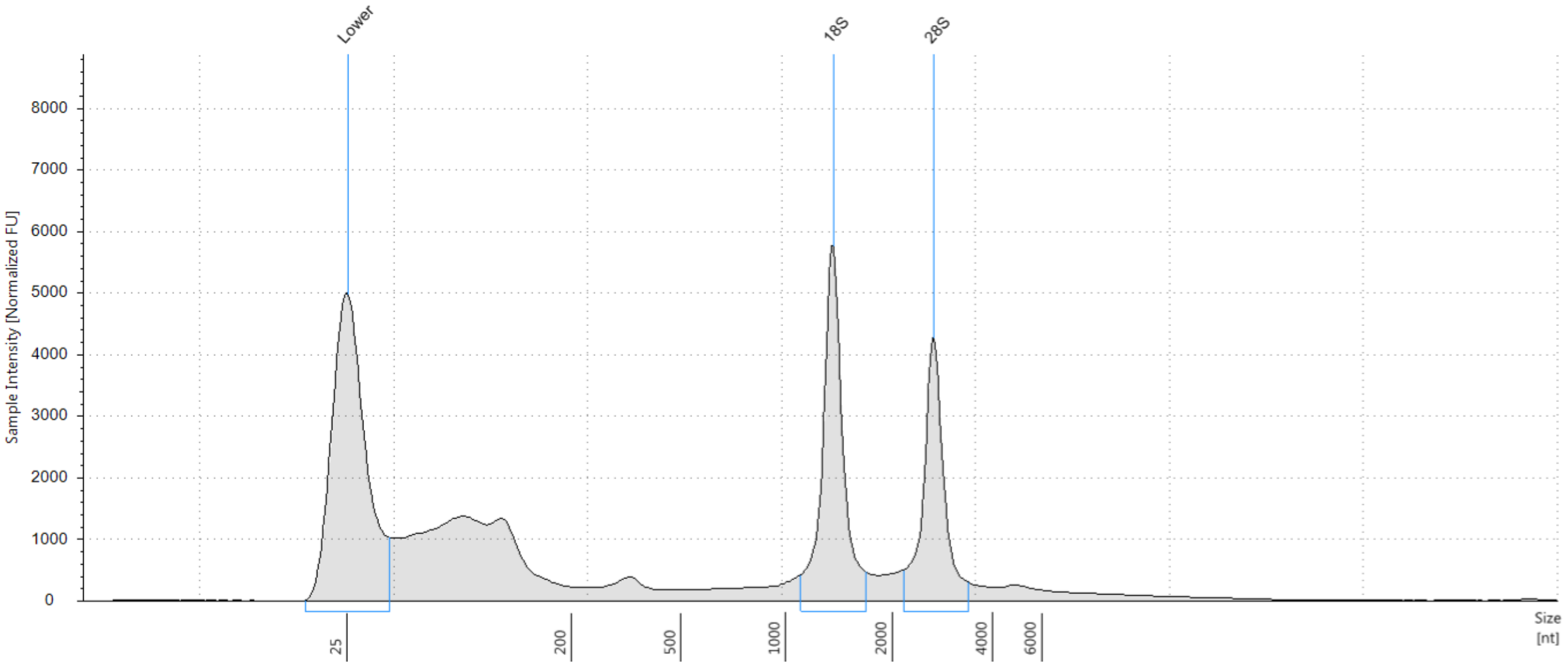

Sample Table

Well	RINe	28S/18S (Area)	Conc. [ng/µl]	Sample Description	Alert	Observations
D3	10.0	0.8	54.4	GK19-ASB-8-10-17-RNA-PA14-w-BIT-5-0C-3		

Peak Table

Size [nt]	Calibrated Conc. [ng/µl]	Assigned Conc. [ng/µl]	Peak Molarity [nmol/l]	% Integrated Area	Peak Comment	Observations
25	40.0	40.0	4710	-		Lower Marker
1357	14.1	-	30.6	56.92		18S
2661	10.7	-	11.8	43.08		28S

E3: GK20-ASB-8-10-17-RNA-PA14-w-BIT-5-0D-3

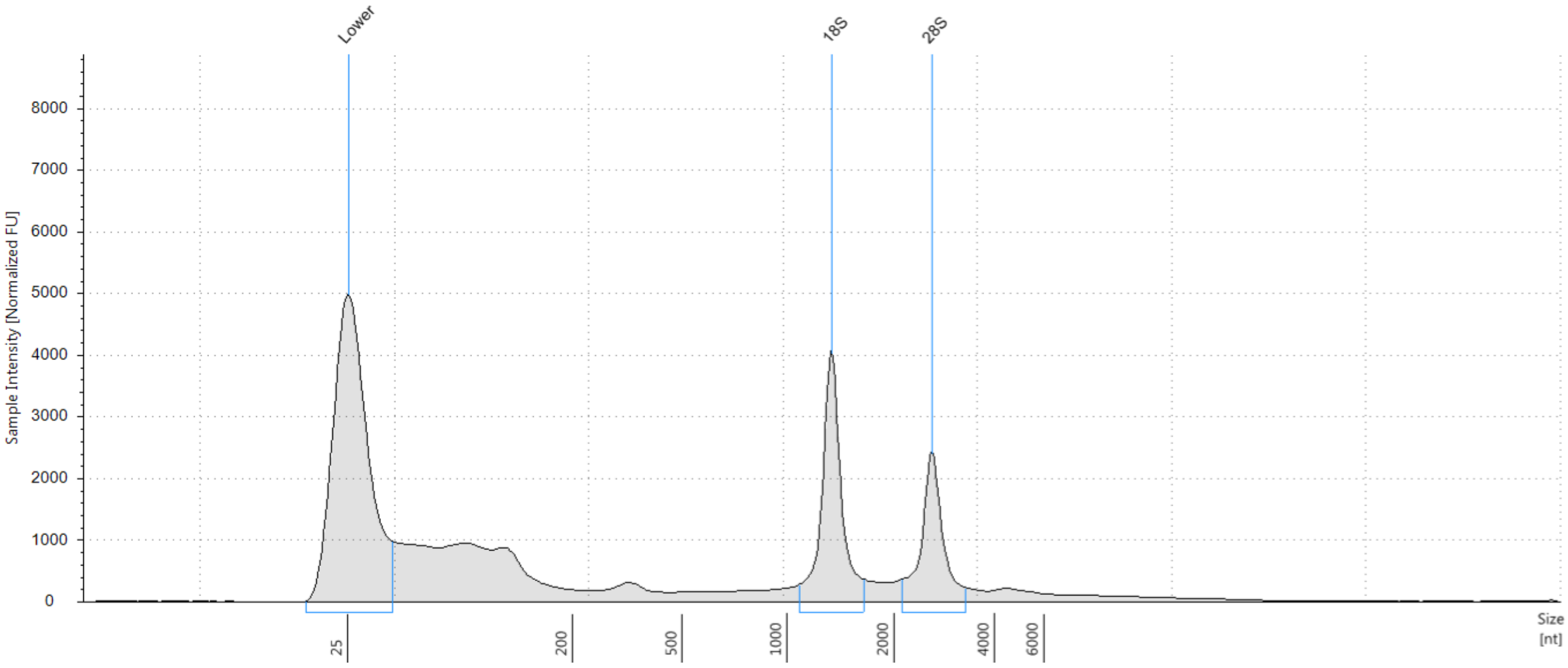

Sample Table

Well	RINe	28S/18S (Area)	Conc. [ng/µl]	Sample Description	Alert	Observations
E3	10.0	0.7	37.4	GK20-ASB-8-10-17-RNA-PA14-w-BIT-5-0D-3		

Peak Table

Size [nt]	Calibrated Conc. [ng/µl]	Assigned Conc. [ng/µl]	Peak Molarity [nmol/l]	% Integrated Area	Peak Comment	Observations
25	40.0	40.0	4710	-		Lower Marker
1334	9.28	-	20.5	60.25		18S
2583	6.12	-	6.97	39.75		28S

 © Agilent Technologies, Inc. 2017

F3: GK21-ASB-8-30-17-RNA-PA14-w-BIT-5-0A-2

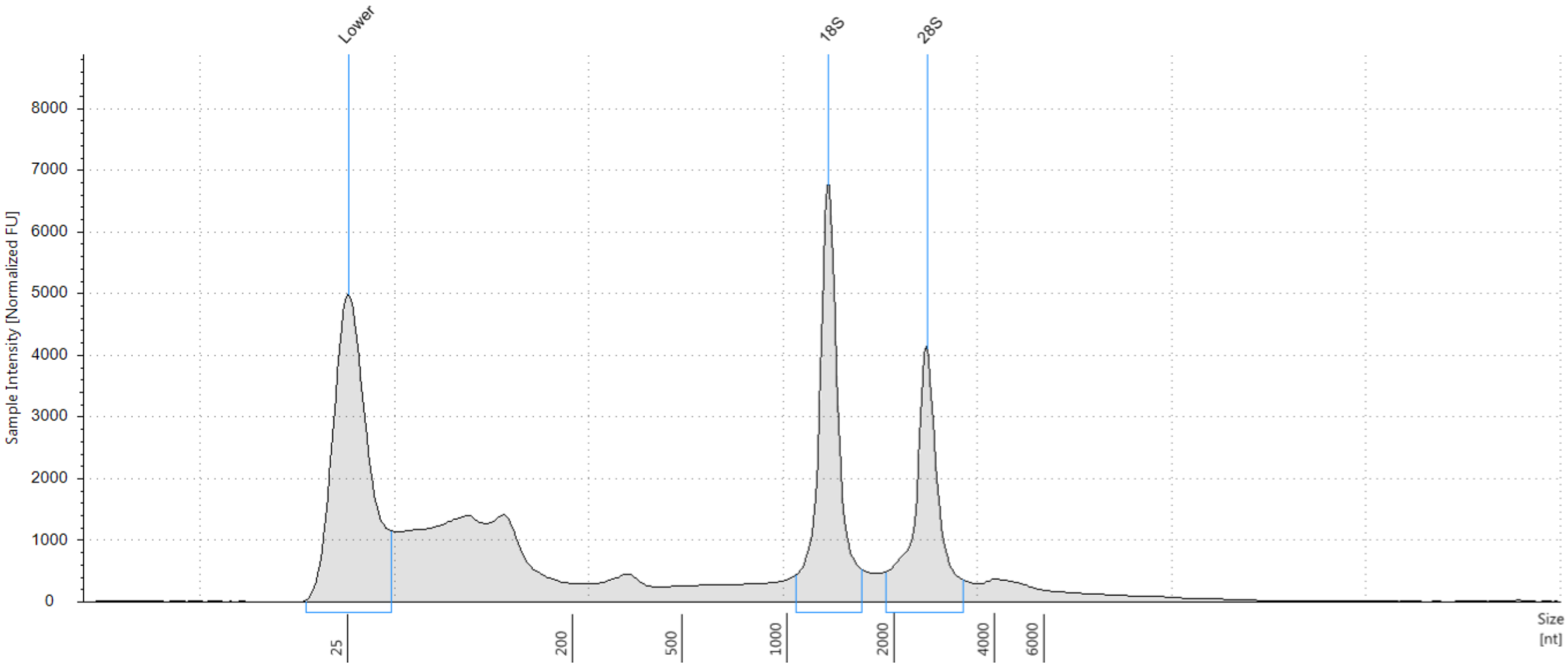

Sample Table

Well	RINe	28S/18S (Area)	Conc. [ng/µl]	Sample Description	Alert	Observations
F3	10.0	0.7	58.4	GK21-ASB-8-30-17-RNA-PA14-w-BIT-5-0A-2		

Peak Table

Size [nt]	Calibrated Conc. [ng/µl]	Assigned Conc. [ng/µl]	Peak Molarity [nmol/l]	% Integrated Area	Peak Comment	Observations
25	40.0	40.0	4710	-		Lower Marker
1303	15.7	-	35.4	58.16		18S
2498	11.3	-	13.3	41.84		28S

G3: GK22-ASB-8-30-17-RNA-PA14-w-BIT-5-0B-2

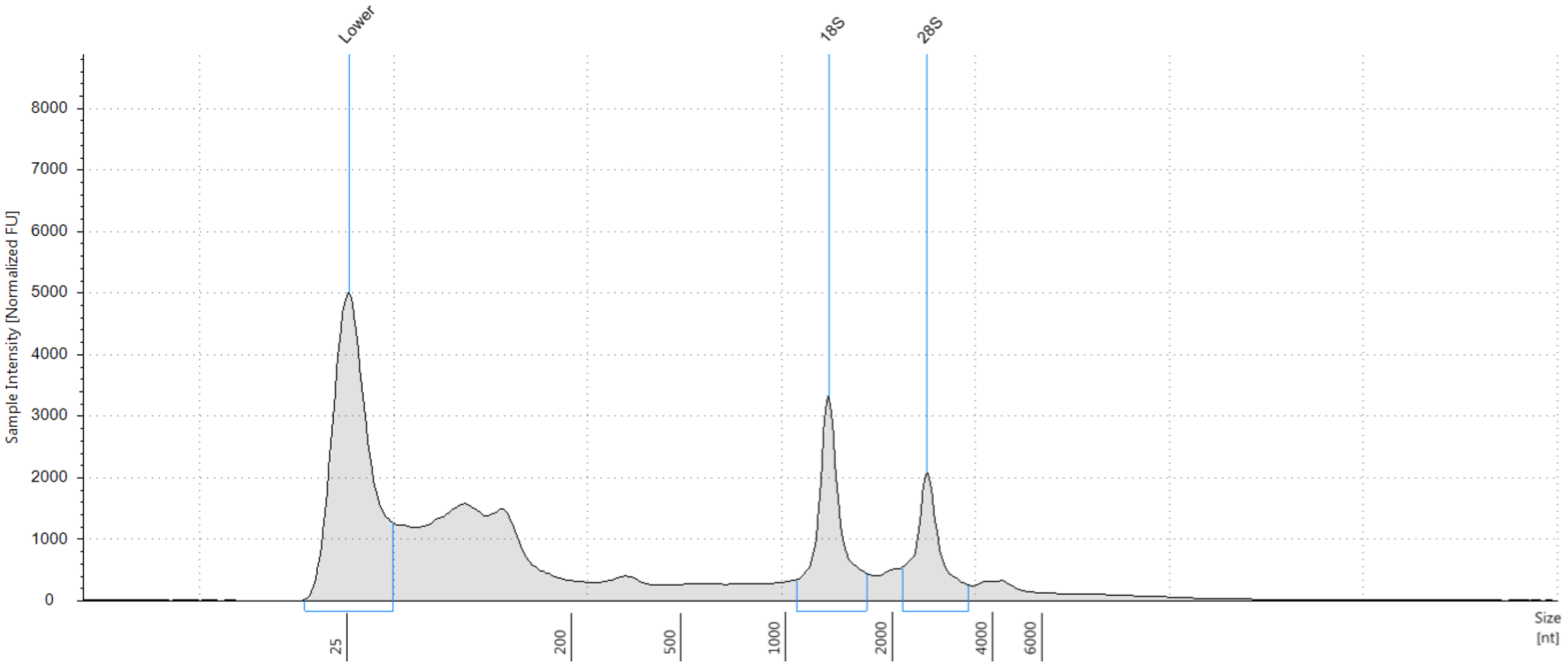

Sample Table

Well	RINe	28S/18S (Area)	Conc. [ng/µl]	Sample Description	Alert	Observations
G3	9.5	0.7	44.3	GK22-ASB-8-30-17-RNA-PA14-w-BIT-5-0B-2		

Peak Table

Size [nt]	Calibrated Conc. [ng/µl]	Assigned Conc. [ng/µl]	Peak Molarity [nmol/l]	% Integrated Area	Peak Comment	Observations
25	40.0	40.0	4710	-		Lower Marker
1318	8.35	-	18.6	59.51		18S
2520	5.68	-	6.63	40.49		28S

H3: GK23-ASB-8-30-17-RNA-PA14-w-BIT-5-0C-2

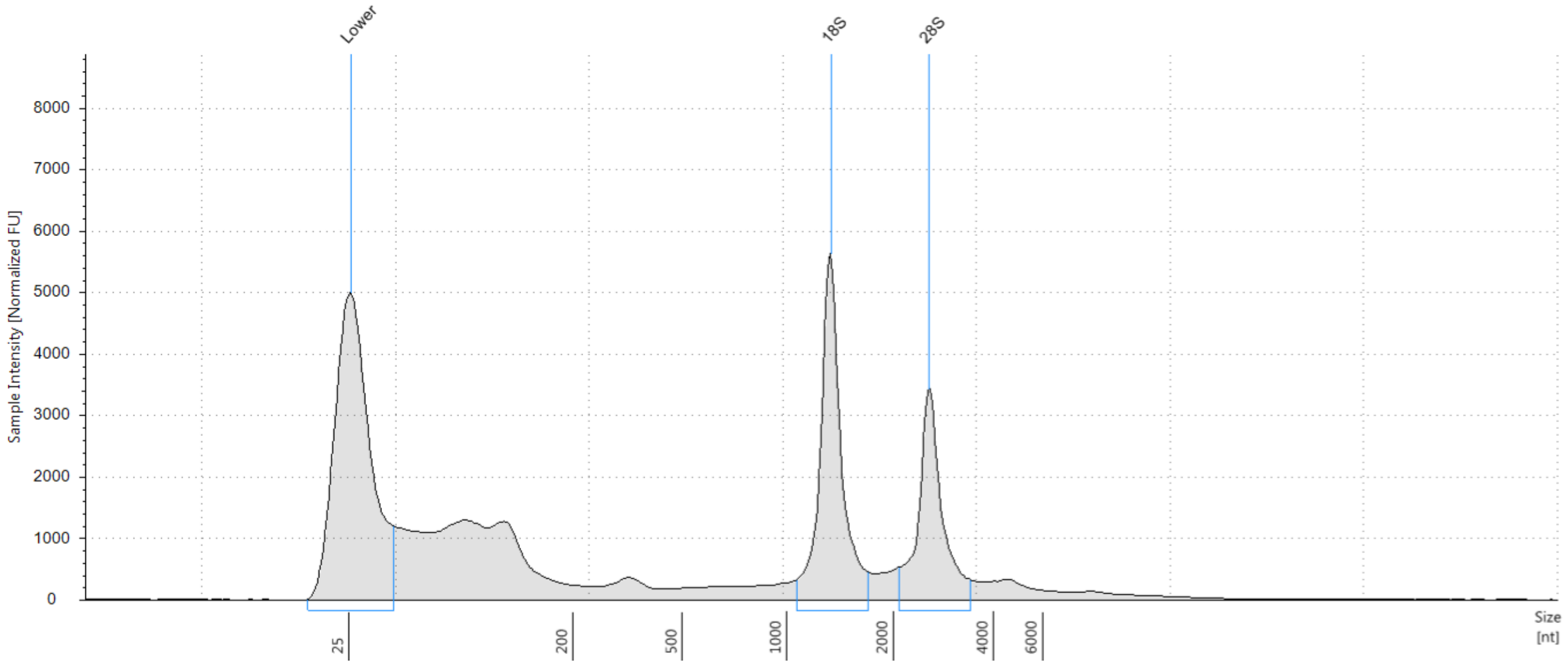

Sample Table

Well	RINe	28S/18S (Area)	Conc. [ng/µl]	Sample Description	Alert	Observations
H3	10.0	0.7	50.2	GK23-ASB-8-30-17-RNA-PA14-w-BIT-5-0C-2		

Peak Table

Size [nt]	Calibrated Conc. [ng/µl]	Assigned Conc. [ng/µl]	Peak Molarity [nmol/l]	% Integrated Area	Peak Comment	Observations
25	40.0	40.0	4710	-		Lower Marker
1323	13.7	-	30.5	59.45		18S
2538	9.35	-	10.8	40.55		28S

 © Agilent Technologies, Inc. 2017

A4: GK24-ASB-8-30-17-RNA-PA14-w-BIT-5-0D-2

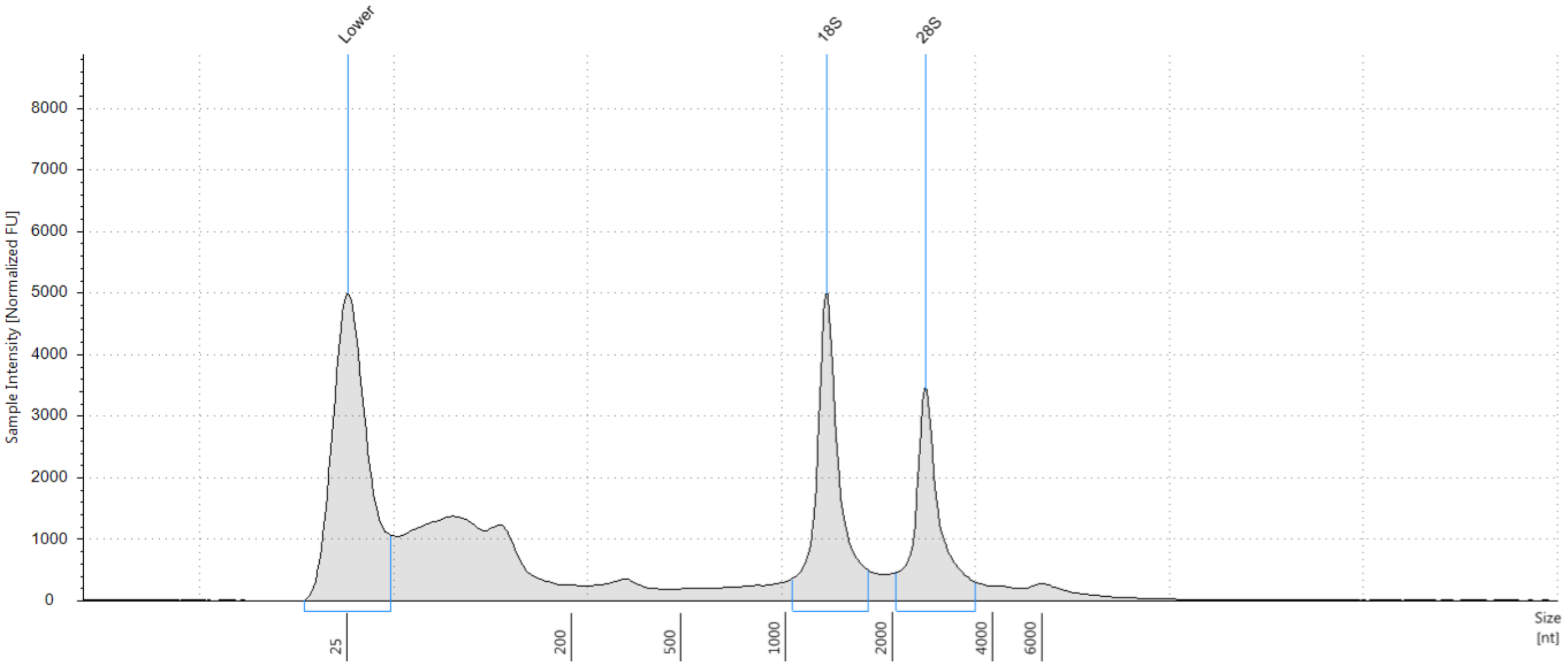

Sample Table

Well	RINe	28S/18S (Area)	Conc. [ng/µl]	Sample Description	Alert	Observations
A4	10.0	0.7	50.2	GK24-ASB-8-30-17-RNA-PA14-w-BIT-5-0D-2		

Peak Table

Size [nt]	Calibrated Conc. [ng/µl]	Assigned Conc. [ng/µl]	Peak Molarity [nmol/l]	% Integrated Area	Peak Comment	Observations
25	40.0	40.0	4710	-		Lower Marker
1300	13.3	-	30.1	57.48		18S
2512	9.84	-	11.5	42.52		28S

 © Agilent Technologies, Inc. 2017

B4: GK25-ASB-8-10-17-RNA-PA14-w-BIT-5-0-1A-3

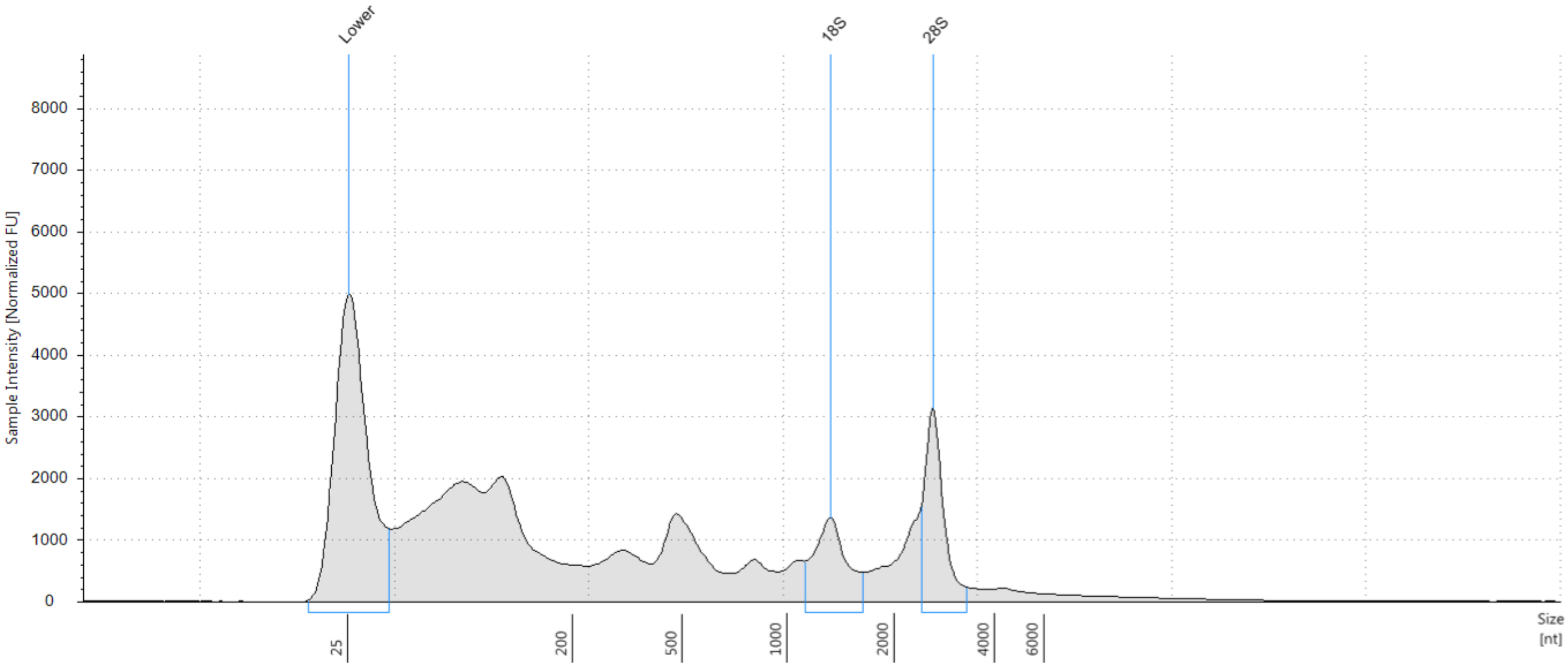

Sample Table

Well	RINe	28S/18S (Area)	Conc. [ng/µl]	Sample Description	Alert	Observations
B4	5.5	1.4	66.7	GK25-ASB-8-10-17-RNA-PA14-w-BIT-5-0-1A-3		

Peak Table

Size [nt]	Calibrated Conc. [ng/µl]	Assigned Conc. [ng/µl]	Peak Molarity [nmol/l]	% Integrated Area	Peak Comment	Observations
25	40.0	40.0	4710	-		Lower Marker
1321	5.49	-	12.2	42.52		18S
2596	7.42	-	8.41	57.48		28S

 © Agilent Technologies, Inc. 2017

C4: GK26-ASB-8-10-17-RNA-PA14-w-BIT-5--0-1B-3

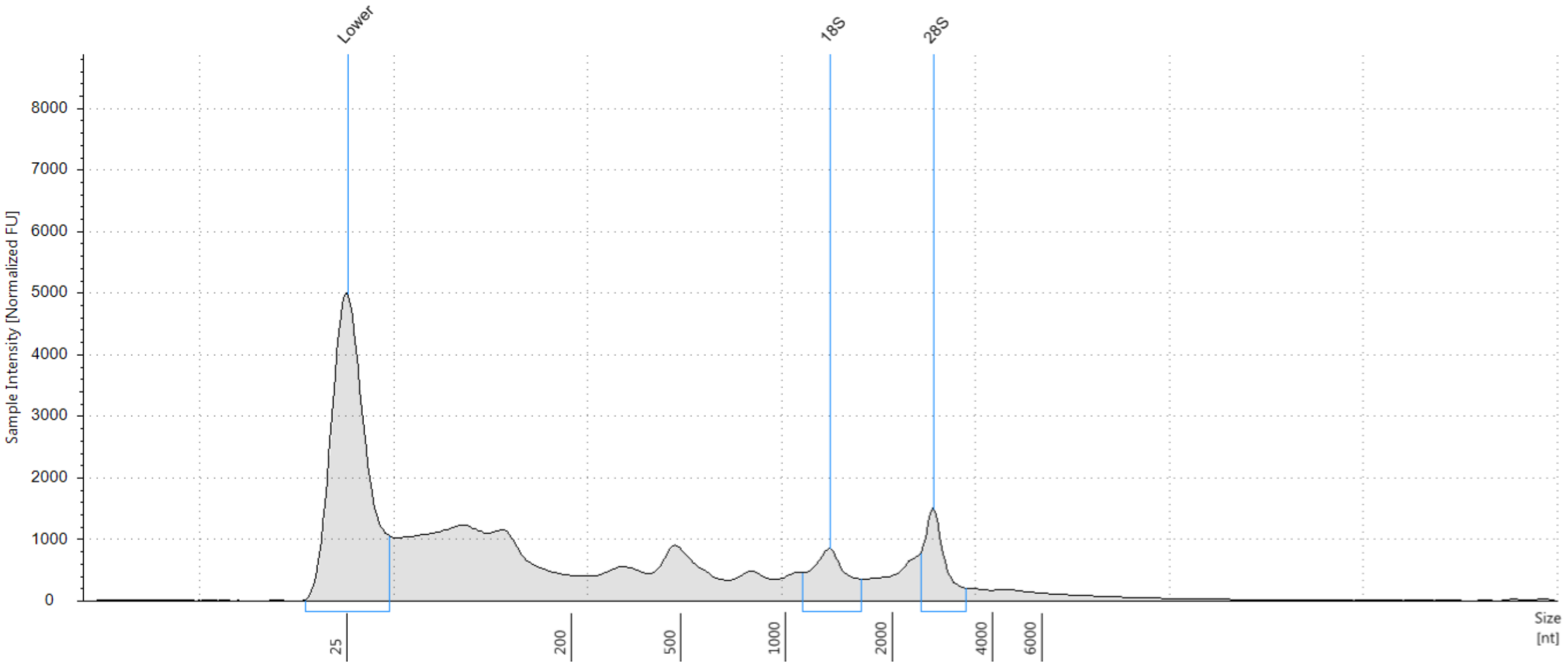

Sample Table

Well	RINe	28S/18S (Area)	Conc. [ng/µl]	Sample Description	Alert	Observations
C4	5.0	1.0	41.5	GK26-ASB-8-10-17-RNA-PA14-w-BIT-5--0-1B-3		

Peak Table

Size [nt]	Calibrated Conc. [ng/µl]	Assigned Conc. [ng/µl]	Peak Molarity [nmol/l]	% Integrated Area	Peak Comment	Observations
25	40.0	40.0	4710	-		Lower Marker
1329	3.48	-	7.69	49.12		18S
2646	3.60	-	4.00	50.88		28S

 © Agilent Technologies, Inc. 2017

D4: GK27-ASB-8-10-17-RNA-PA14-w-BIT-5-0-1C-3

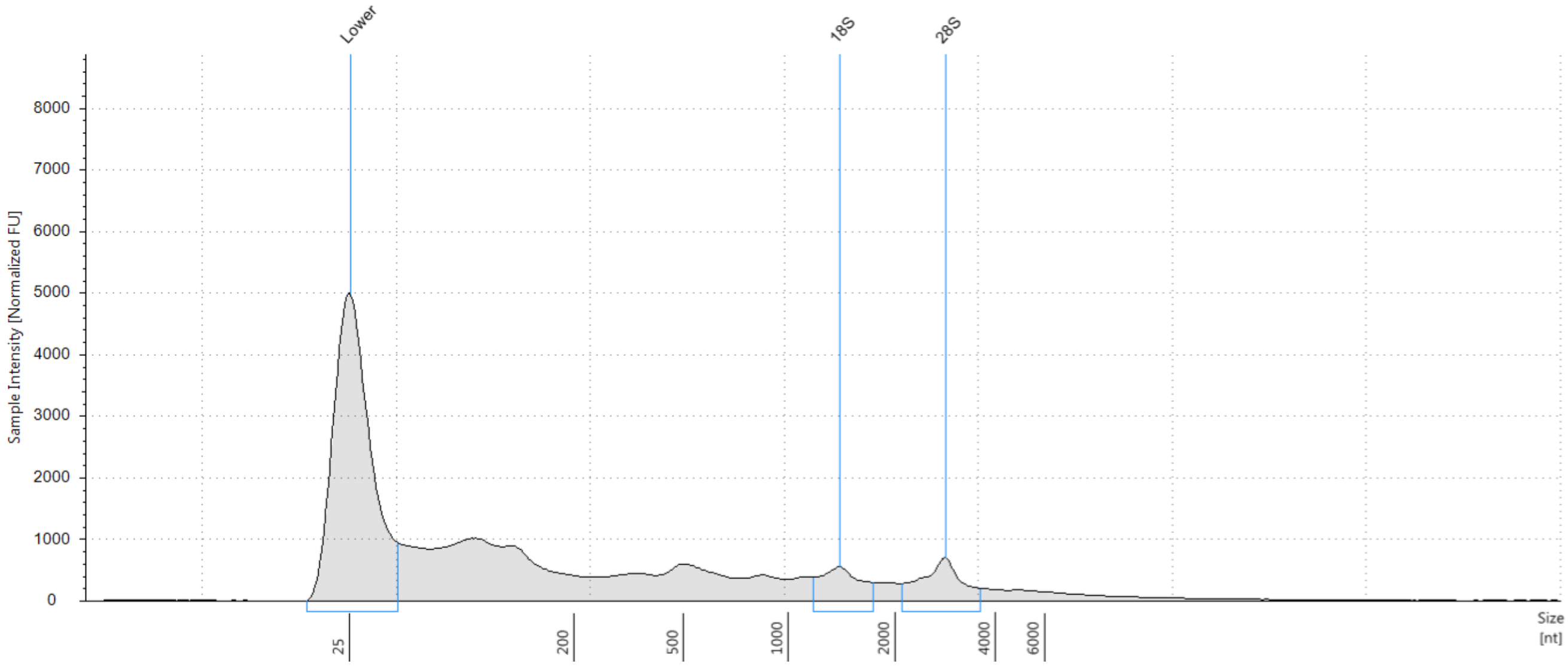

Sample Table

Well	RINe	28S/18S (Area)	Conc. [ng/µl]	Sample Description	Alert	Observations
D4	3.9	1.2	31.5	GK27-ASB-8-10-17-RNA-PA14-w-BIT-5-0-1C-3		

Peak Table

Size [nt]	Calibrated Conc. [ng/µl]	Assigned Conc. [ng/µl]	Peak Molarity [nmol/l]	% Integrated Area	Peak Comment	Observations
25	40.0	40.0	4710	-		Lower Marker
1388	2.41	-	5.10	45.01		18S
2817	2.94	-	3.07	54.99		28S

 © Agilent Technologies, Inc. 2017

E4: GK28-ASB-8-10-17-RNA-PA14-w-BIT-5-0-1F-3

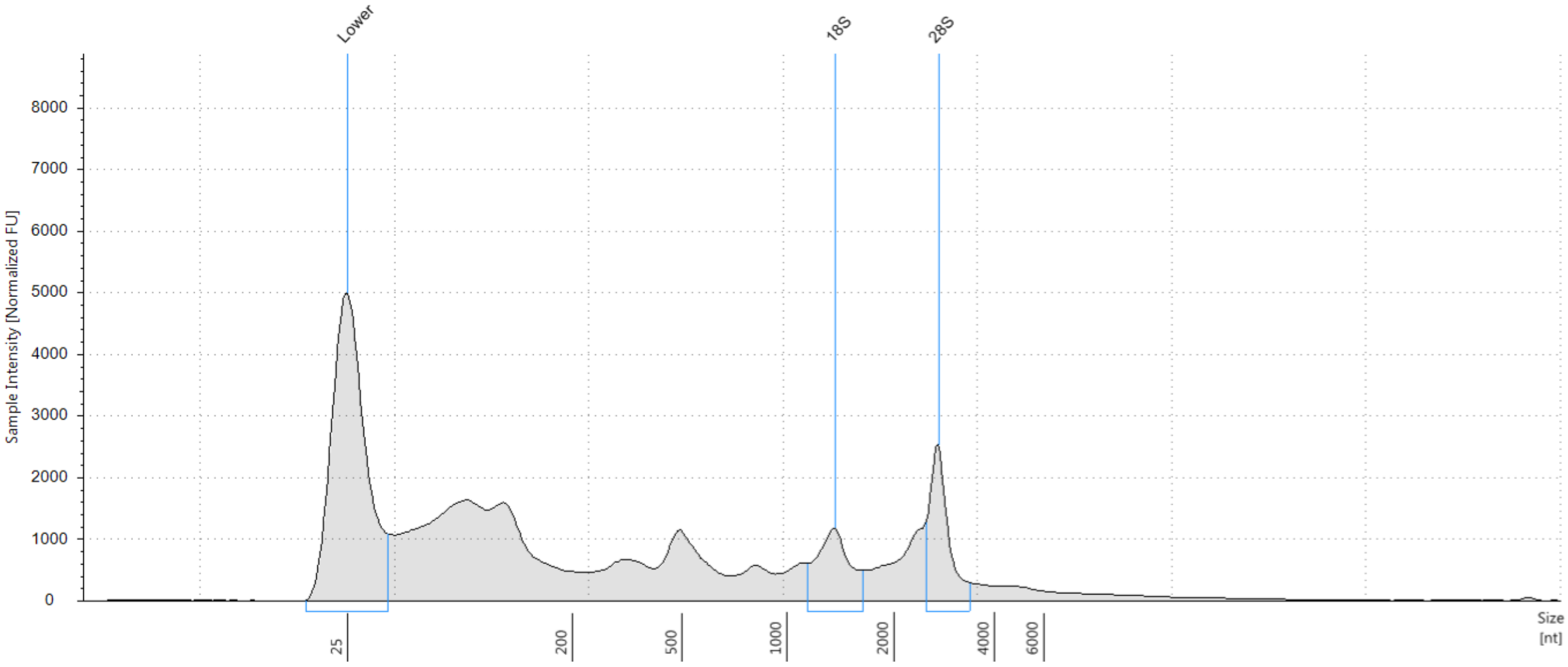

Sample Table

Well	RINe	28S/18S (Area)	Conc. [ng/µl]	Sample Description	Alert	Observations
E4	5.4	1.2	56.3	GK28-ASB-8-10-17-RNA-PA14-w-BIT-5-0-1F-3		

Peak Table

Size [nt]	Calibrated Conc. [ng/µl]	Assigned Conc. [ng/µl]	Peak Molarity [nmol/l]	% Integrated Area	Peak Comment	Observations
25	40.0	40.0	4710	-		Lower Marker
1370	4.71	-	10.1	44.93		18S
2719	5.77	-	6.24	55.07		28S

© Agilent Technologies, Inc. 2017

F4: GK29-ASB-8-30-17-RNA-PA14-w-BIT-5-0-1A-2

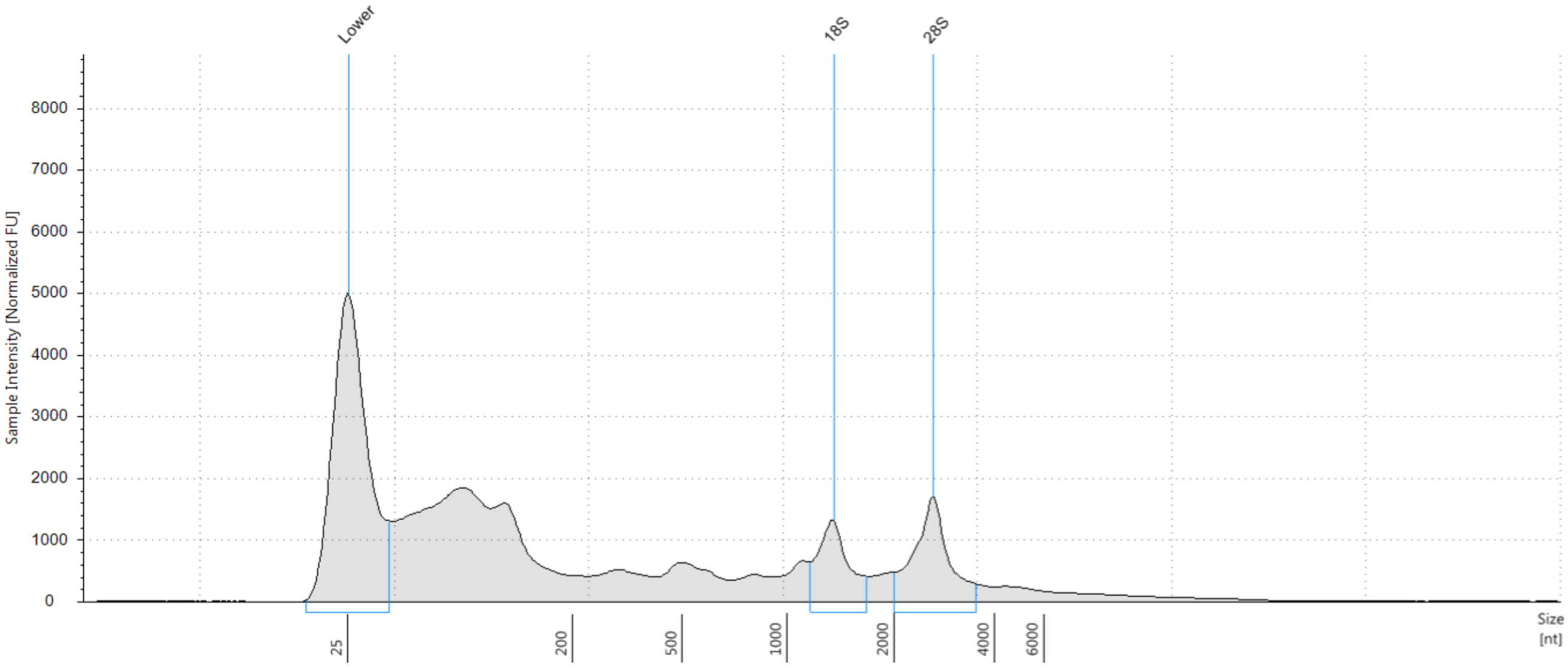

Sample Table

Well	RINe	28S/18S (Area)	Conc. [ng/µl]	Sample Description	Alert	Observations
F4	6.3	1.4	51.5	GK29-ASB-8-30-17-RNA-PA14-w-BIT-5-0-1A-2		

Peak Table

Size [nt]	Calibrated Conc. [ng/µl]	Assigned Conc. [ng/µl]	Peak Molarity [nmol/l]	% Integrated Area	Peak Comment	Observations
25	40.0	40.0	4710	-		Lower Marker
1346	4.69	-	10.3	41.00		18S
2608	6.76	-	7.62	59.00		28S

 © Agilent Technologies, Inc. 2017

G4: GK30-ASB-8-30-17-RNA-PA14-w-BIT-5--0-1B-2

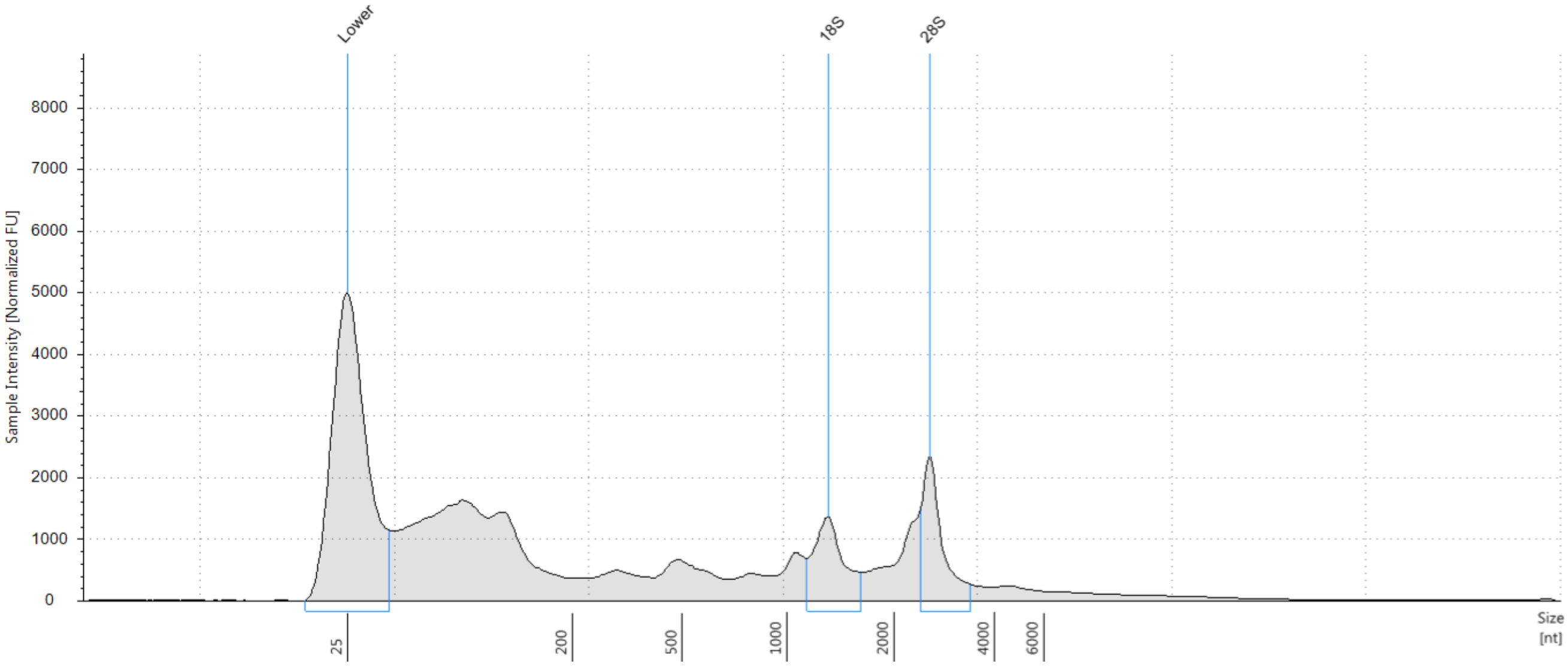

Sample Table

Well	RINe	28S/18S (Area)	Conc. [ng/µl]	Sample Description	Alert	Observations
G4	6.4	1.2	51.0	GK30-ASB-8-30-17-RNA-PA14-w-BIT-5--0-1B-2		

Peak Table

Size [nt]	Calibrated Conc. [ng/µl]	Assigned Conc. [ng/µl]	Peak Molarity [nmol/l]	% Integrated Area	Peak Comment	Observations
25	40.0	40.0	4710	-		Lower Marker
1311	4.79	-	10.7	46.31		18S
2567	5.55	-	6.36	53.69		28S

 © Agilent Technologies, Inc. 2017

H4: GK31-ASB-8-30-17-RNA-PA14-w-BIT-5-0-1D-2

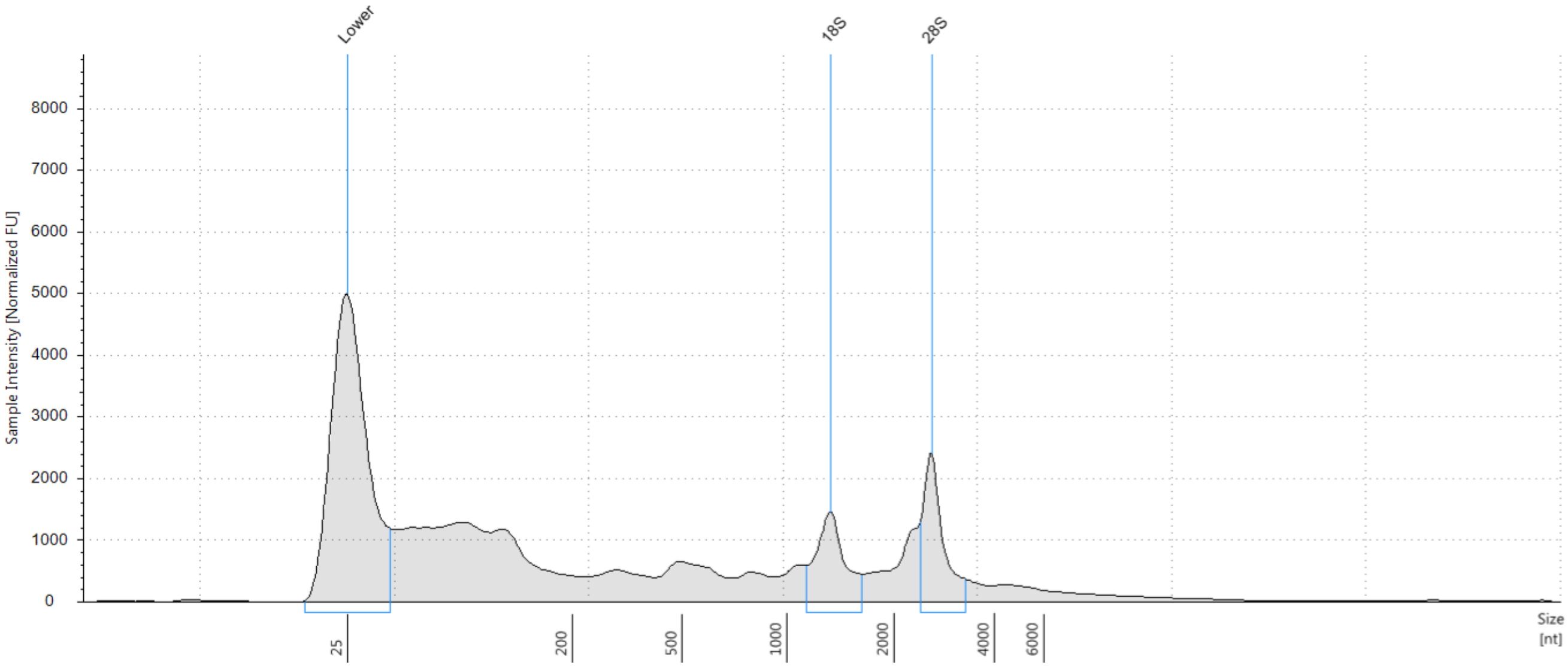

Sample Table

Well	RINe	28S/18S (Area)	Conc. [ng/µl]	Sample Description	Alert	Observations
H4	6.5	1.1	46.7	GK31-ASB-8-30-17-RNA-PA14-w-BIT-5-0-1D-2		

Peak Table

Size [nt]	Calibrated Conc. [ng/µl]	Assigned Conc. [ng/µl]	Peak Molarity [nmol/l]	% Integrated Area	Peak Comment	Observations
25	40.0	40.0	4710	-		Lower Marker
1325	4.75	-	10.5	46.83		18S
2586	5.39	-	6.13	53.17		28S

 © Agilent Technologies, Inc. 2017

A5: GK32-ASB-8-30-17-RNA-PA14-w-BIT-5-0-1F-2

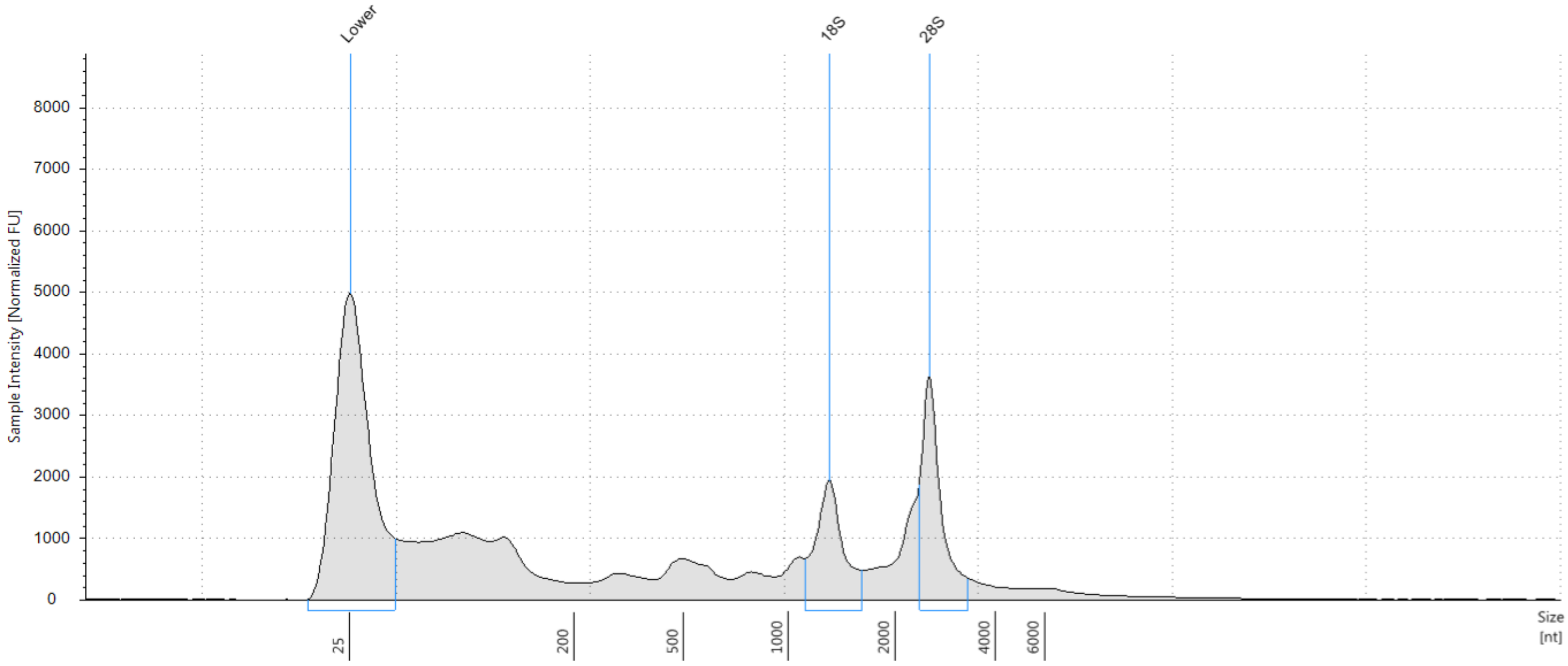

Sample Table

Well	RINe	28S/18S (Area)	Conc. [ng/µl]	Sample Description	Alert	Observations
A5	7.5	1.3	46.3	GK32-ASB-8-30-17-RNA-PA14-w-BIT-5-0-1F-2		

Peak Table

Size [nt]	Calibrated Conc. [ng/µl]	Assigned Conc. [ng/µl]	Peak Molarity [nmol/l]	% Integrated Area	Peak Comment	Observations
25	40.0	40.0	4710	-		Lower Marker
1303	6.13	-	13.8	43.11		18S
2517	8.09	-	9.46	56.89		28S

B5: GK33-ASB-8-10-17-RNA-PA14-w-BIT-24-0A-3

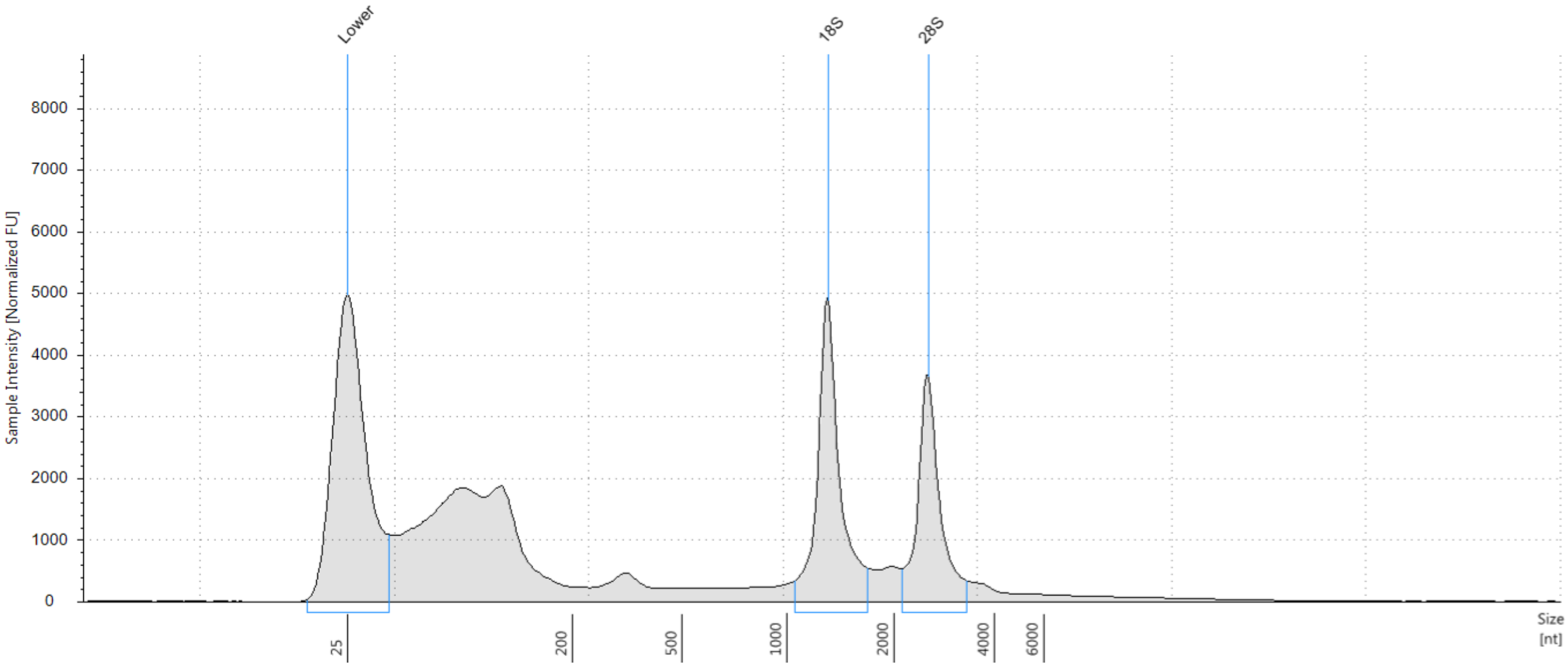

Sample Table

Well	RINe	28S/18S (Area)	Conc. [ng/µl]	Sample Description	Alert	Observations
B5	10.0	0.7	57.7	GK33-ASB-8-10-17-RNA-PA14-w-BIT-24-0A-3		

Peak Table

Size [nt]	Calibrated Conc. [ng/µl]	Assigned Conc. [ng/µl]	Peak Molarity [nmol/l]	% Integrated Area	Peak Comment	Observations
25	40.0	40.0	4710	-		Lower Marker
1304	13.4	-	30.2	57.35		18S
2529	9.94	-	11.6	42.65		28S

 © Agilent Technologies, Inc. 2017

C5: GK34-ASB-8-10-17-RNA-PA14-w-BIT-24-0B-3

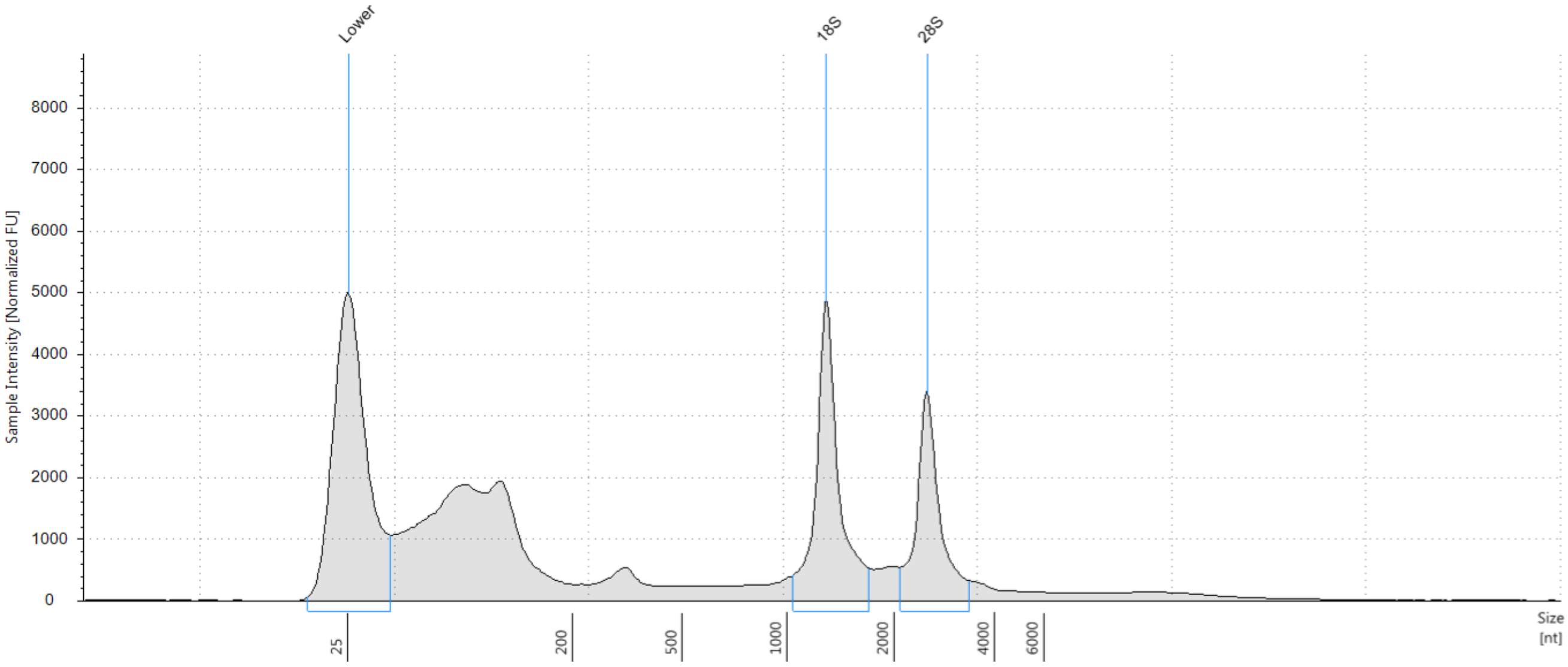

Sample Table

Well	RINe	28S/18S (Area)	Conc. [ng/µl]	Sample Description	Alert	Observations
C5	9.9	0.7	59.3	GK34-ASB-8-10-17-RNA-PA14-w-BIT-24-0B-3		

Peak Table

Size [nt]	Calibrated Conc. [ng/µl]	Assigned Conc. [ng/µl]	Peak Molarity [nmol/l]	% Integrated Area	Peak Comment	Observations
25	40.0	40.0	4710	-		Lower Marker
1282	13.4	-	30.8	58.63		18S
2513	9.46	-	11.1	41.37		28S

D5: GK35-ASB-8-10-17-RNA-PA14-w-BIT-24-0C-3

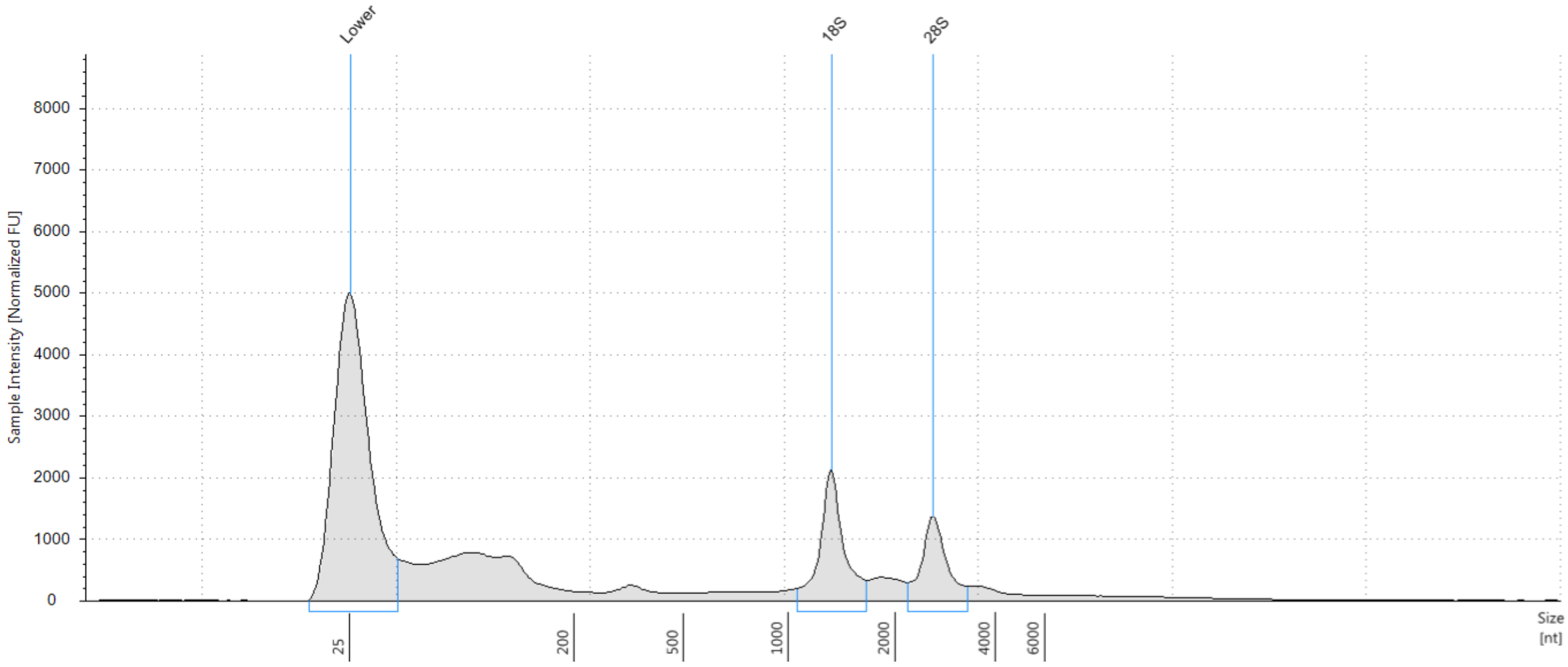

Sample Table

Well	RINe	28S/18S (Area)	Conc. [ng/µl]	Sample Description	Alert	Observations
D5	9.7	0.7	26.6	GK35-ASB-8-10-17-RNA-PA14-w-BIT-24-0C-3		

Peak Table

Size [nt]	Calibrated Conc. [ng/µl]	Assigned Conc. [ng/µl]	Peak Molarity [nmol/l]	% Integrated Area	Peak Comment	Observations
25	40.0	40.0	4710	-		Lower Marker
1318	5.75	-	12.8	59.27		18S
2597	3.95	-	4.48	40.73		28S

 © Agilent Technologies, Inc. 2017

E5: GK36-ASB-8-10-17-RNA-PA14-w-BIT-24-0D-3

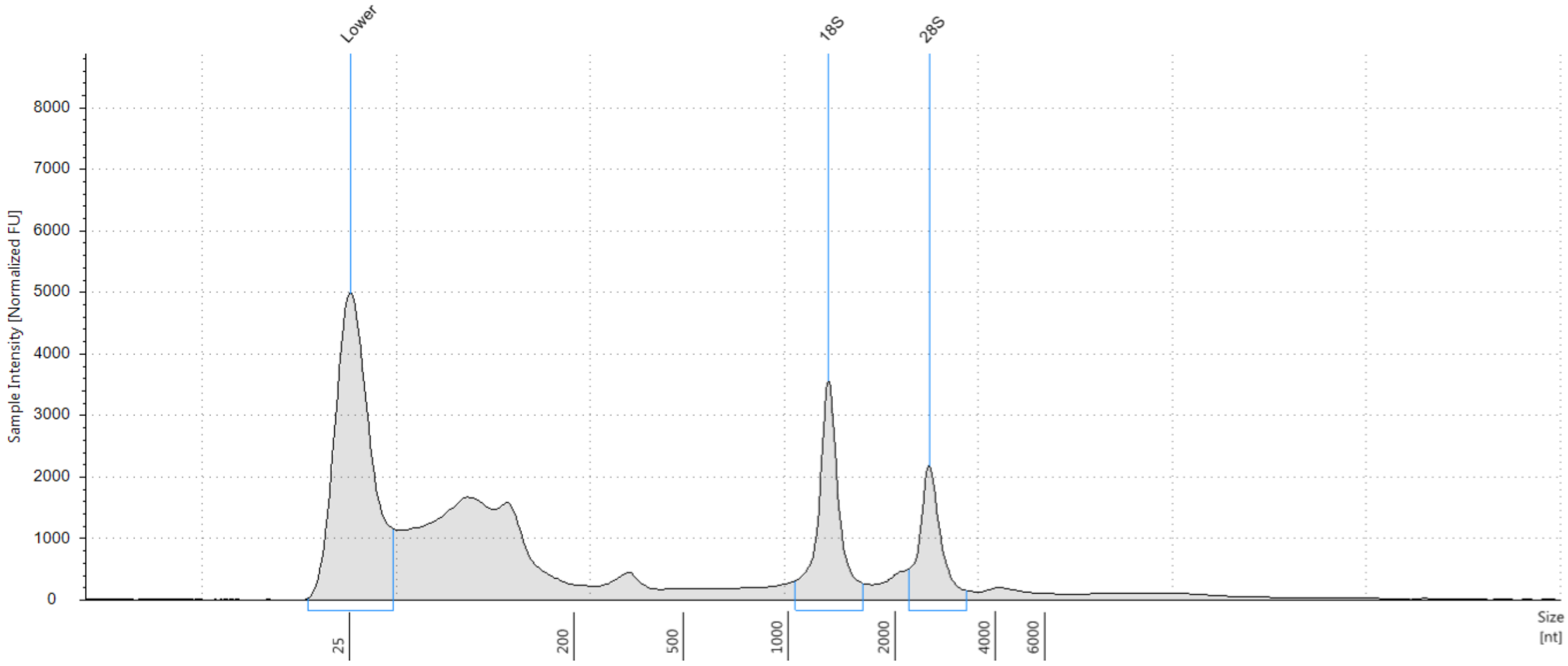

Sample Table

Well	RINe	28S/18S (Area)	Conc. [ng/µl]	Sample Description	Alert	Observations
E5	9.9	0.6	43.6	GK36-ASB-8-10-17-RNA-PA14-w-BIT-24-0D-3		

Peak Table

Size [nt]	Calibrated Conc. [ng/µl]	Assigned Conc. [ng/µl]	Peak Molarity [nmol/l]	% Integrated Area	Peak Comment	Observations
25	40.0	40.0	4710	-		Lower Marker
1291	8.27	-	18.8	60.78		18S
2517	5.34	-	6.24	39.22		28S

 © Agilent Technologies, Inc. 2017

F5: GK37-ASB-8-30-17-RNA-PA14-w-BIT-24-0A-2

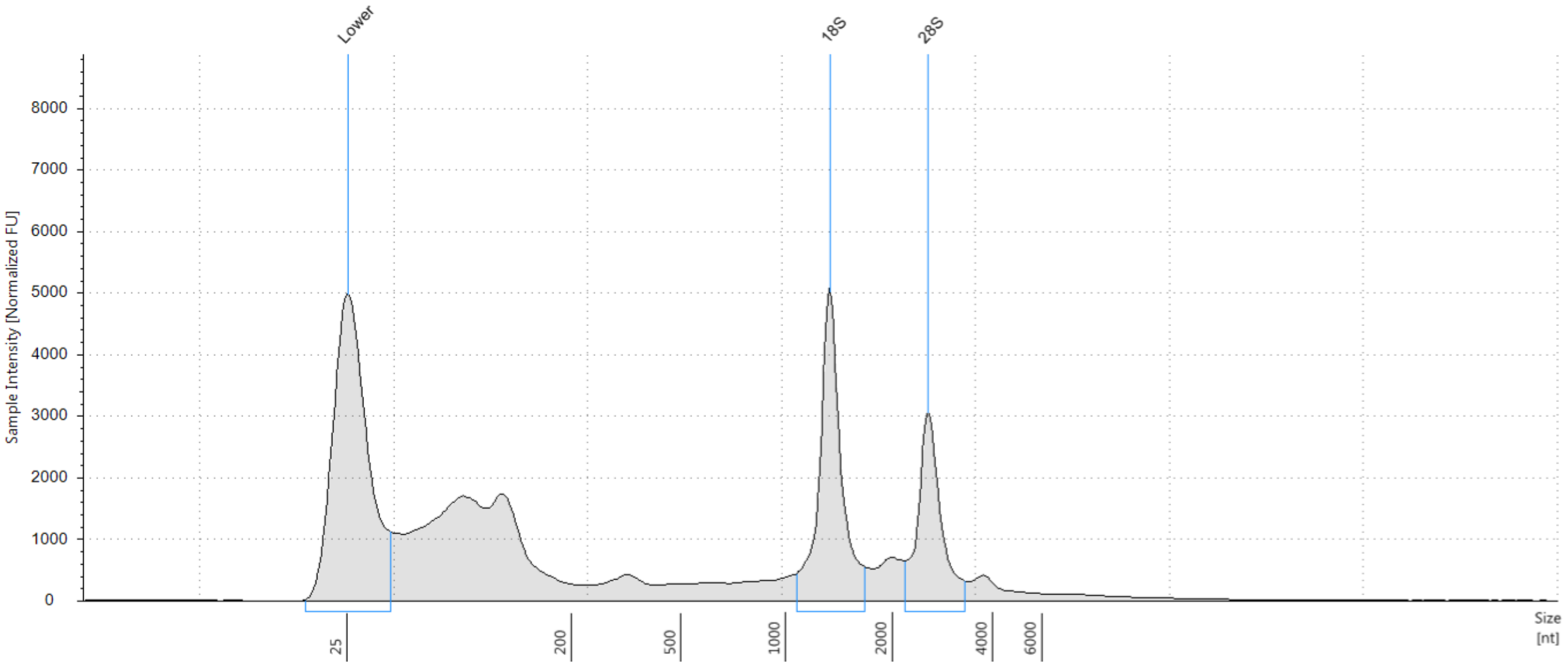

Sample Table

Well	RINe	28S/18S (Area)	Conc. [ng/µl]	Sample Description	Alert	Observations
F5	9.8	0.6	54.6	GK37-ASB-8-30-17-RNA-PA14-w-BIT-24-0A-2		

Peak Table

Size [nt]	Calibrated Conc. [ng/µl]	Assigned Conc. [ng/µl]	Peak Molarity [nmol/l]	% Integrated Area	Peak Comment	Observations
25	40.0	40.0	4710	-		Lower Marker
1336	12.9	-	28.3	61.23		18S
2550	8.14	-	9.39	38.77		28S

 © Agilent Technologies, Inc. 2017

G5: GK38-ASB-8-30-17-RNA-PA14-w-BIT-24-0B-2

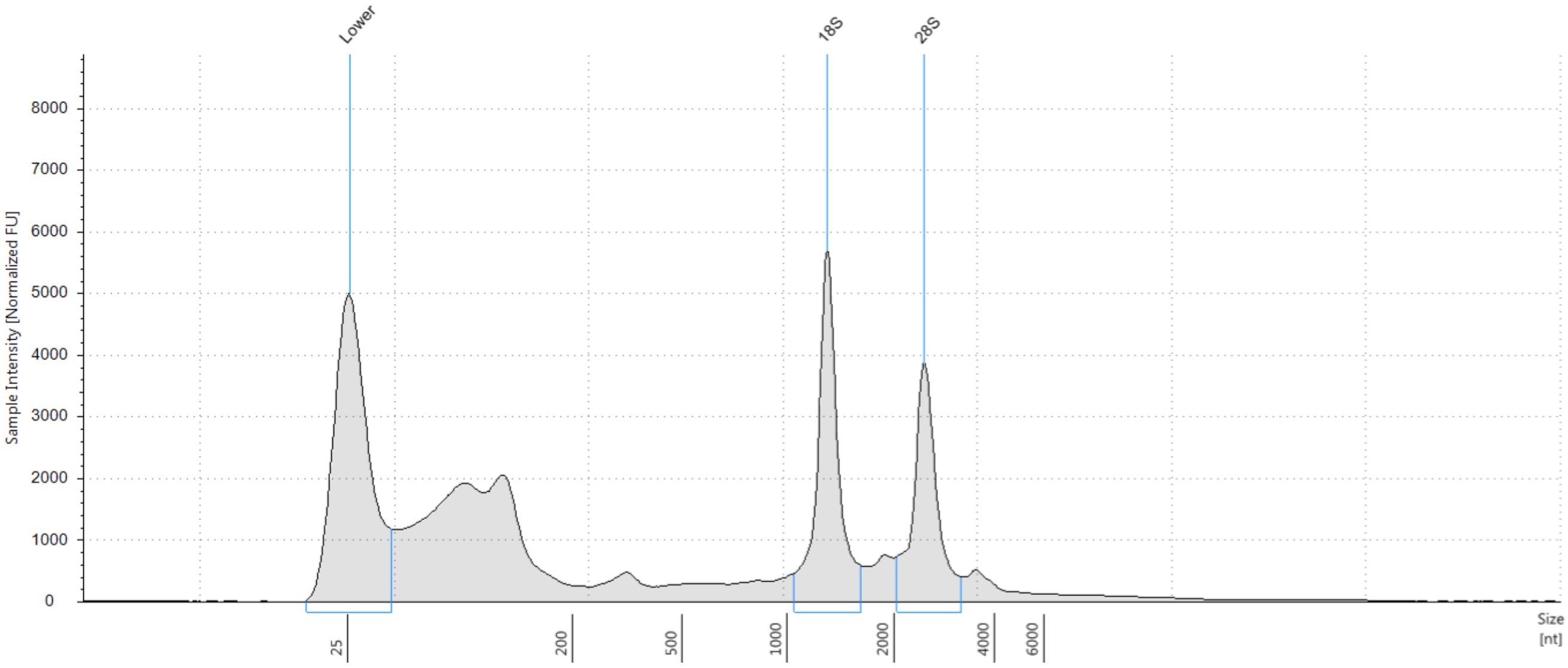

Sample Table

Well	RINe	28S/18S (Area)	Conc. [ng/µl]	Sample Description	Alert	Observations
G5	9.9	0.8	61.2	GK38-ASB-8-30-17-RNA-PA14-w-BIT-24-0B-2		

Peak Table

Size [nt]	Calibrated Conc. [ng/µl]	Assigned Conc. [ng/µl]	Peak Molarity [nmol/l]	% Integrated Area	Peak Comment	Observations
25	40.0	40.0	4710	-		Lower Marker
1292	13.6	-	30.9	56.33		18S
2453	10.5	-	12.6	43.67		28S

 © Agilent Technologies, Inc. 2017

H5: GK39-ASB-8-30-17-RNA-PA14-w-BIT-24-0C-2

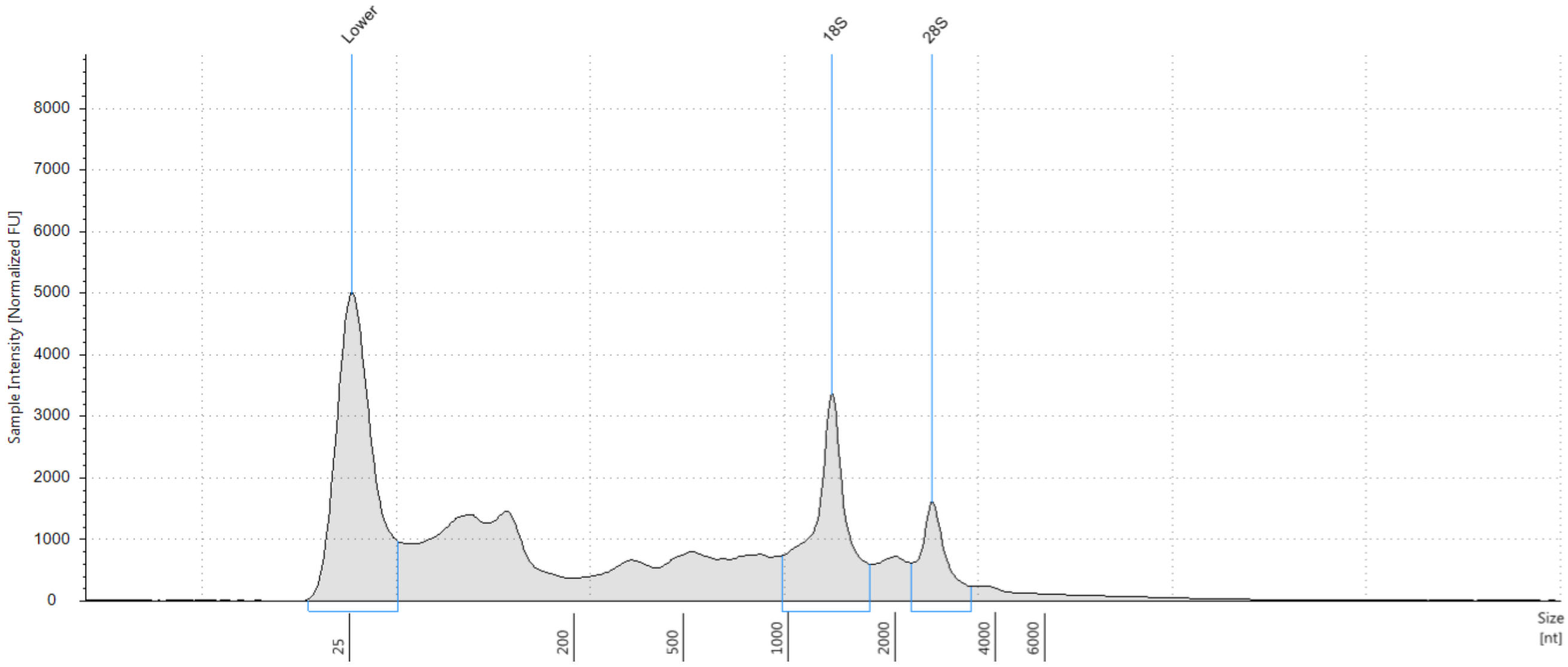

Sample Table

Well	RINe	28S/18S (Area)	Conc. [ng/µl]	Sample Description	Alert	Observations
H5	7.5	0.4	51.7	GK39-ASB-8-30-17-RNA-PA14-w-BIT-24-0C-2		

Peak Table

Size [nt]	Calibrated Conc. [ng/µl]	Assigned Conc. [ng/µl]	Peak Molarity [nmol/l]	% Integrated Area	Peak Comment	Observations
25	40.0	40.0	4710	-		Lower Marker
1318	12.0	-	26.8	72.13		18S
2564	4.65	-	5.33	27.87		28S

 © Agilent Technologies, Inc. 2017

A6: GK40-ASB-8-30-17-RNA-PA14-w-BIT-24-0D-2

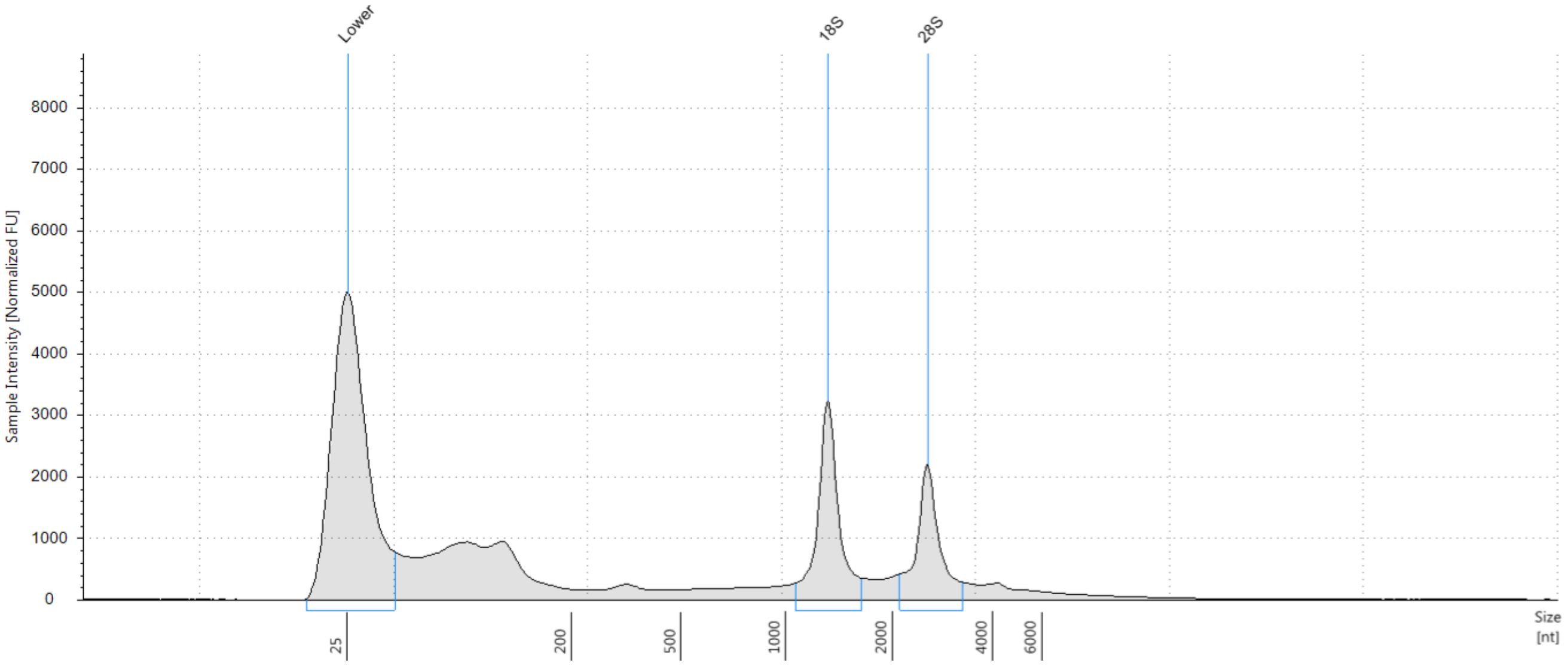

Sample Table

Well	RINe	28S/18S (Area)	Conc. [ng/µl]	Sample Description	Alert	Observations
A6	9.8	0.7	33.1	GK40-ASB-8-30-17-RNA-PA14-w-BIT-24-0D-2		

Peak Table

Size [nt]	Calibrated Conc. [ng/µl]	Assigned Conc. [ng/µl]	Peak Molarity [nmol/l]	% Integrated Area	Peak Comment	Observations
25	40.0	40.0	4710	-		Lower Marker
1312	7.76	-	17.4	57.66		18S
2541	5.70	-	6.59	42.34		28S

B6: GK41-ASB-8-10-17-RNA-PA14-w-BIT-24-0-1A-3

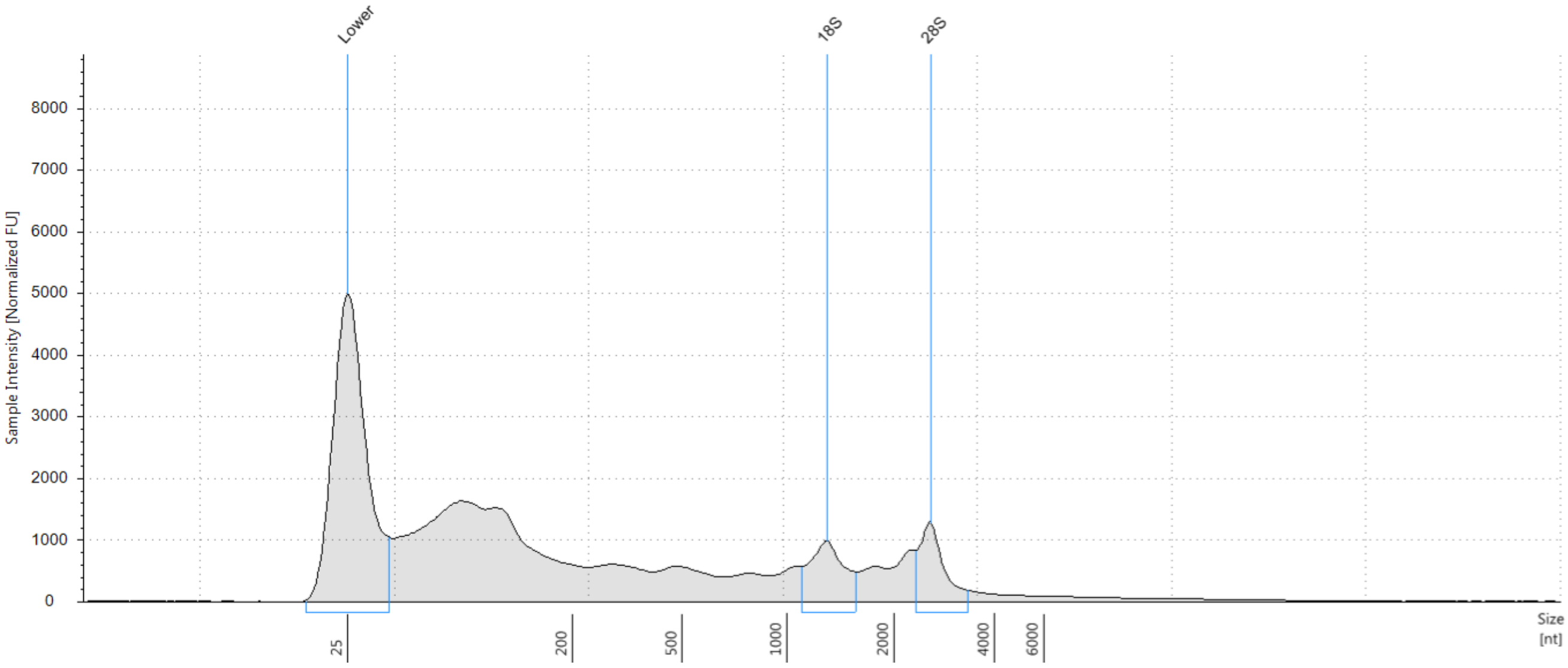

Sample Table

Well	RINe	28S/18S (Area)	Conc. [ng/µl]	Sample Description	Alert	Observations
B6	5.2	0.9	49.4	GK41-ASB-8-10-17-RNA-PA14-w-BIT-24-0-1A-3		

Peak Table

Size [nt]	Calibrated Conc. [ng/µl]	Assigned Conc. [ng/µl]	Peak Molarity [nmol/l]	% Integrated Area	Peak Comment	Observations
25	40.0	40.0	4710	-		Lower Marker
1292	4.11	-	9.36	51.80		18S
2577	3.83	-	4.37	48.20		28S

 © Agilent Technologies, Inc. 2017

C6: GK42-ASB-8-10-17-RNA-PA14-w-BIT-24--0-1B-3

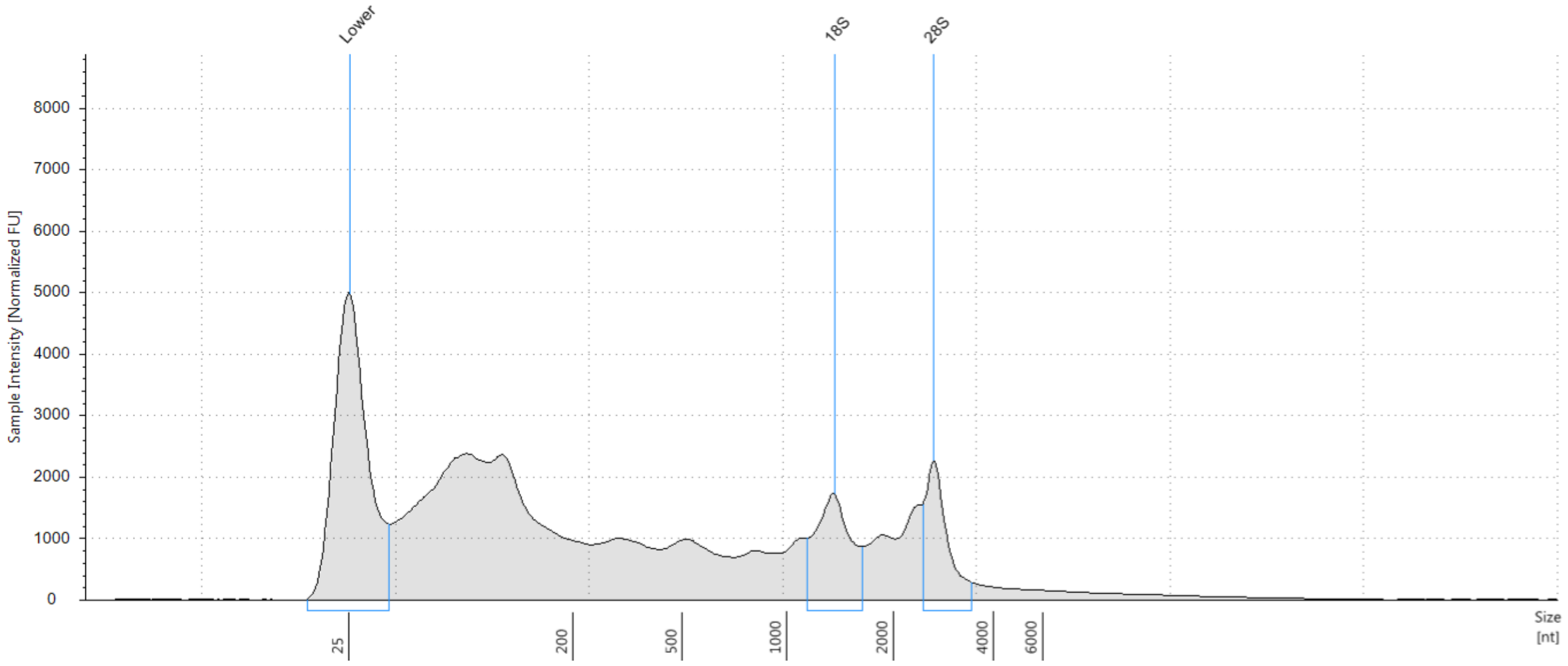

Sample Table

Well	RINe	28S/18S (Area)	Conc. [ng/µl]	Sample Description	Alert	Observations
C6	5.2	0.8	79.7	GK42-ASB-8-10-17-RNA-PA14-w-BIT-24--0-1B-3		

Peak Table

Size [nt]	Calibrated Conc. [ng/µl]	Assigned Conc. [ng/µl]	Peak Molarity [nmol/l]	% Integrated Area	Peak Comment	Observations
25	40.0	40.0	4710	-		Lower Marker
1357	7.46	-	16.2	55.05		18S
2636	6.09	-	6.80	44.95		28S

 © Agilent Technologies, Inc. 2017

D6: GK43-ASB-8-10-17-RNA-PA14-w-BIT-24-0-1C-3

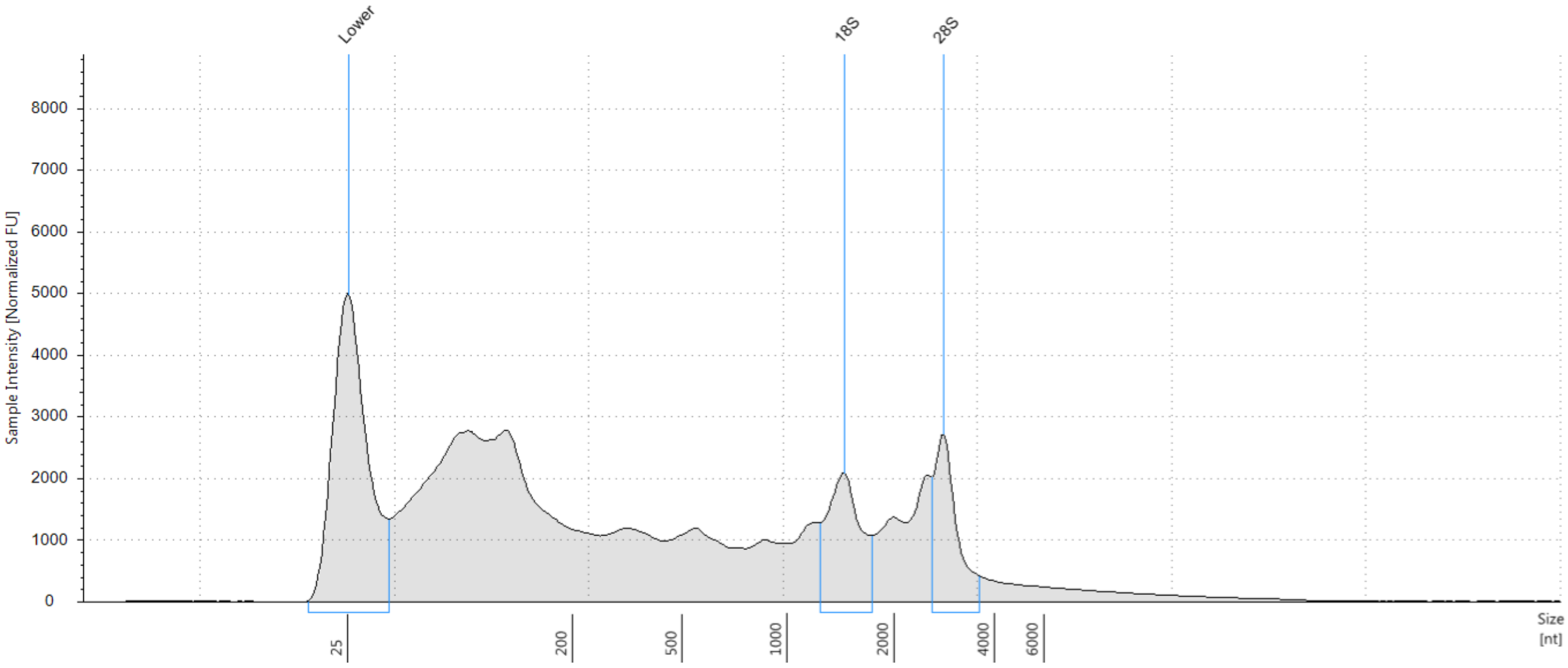

Sample Table

Well	RINe	28S/18S (Area)	Conc. [ng/µl]	Sample Description	Alert	Observations
D6	5.1	0.9	95.2	GK43-ASB-8-10-17-RNA-PA14-w-BIT-24-0-1C-3		

Peak Table

Size [nt]	Calibrated Conc. [ng/µl]	Assigned Conc. [ng/µl]	Peak Molarity [nmol/l]	% Integrated Area	Peak Comment	Observations
25	40.0	40.0	4710	-		Lower Marker
1443	8.47	-	17.3	53.94		18S
2798	7.23	-	7.60	46.06		28S

 © Agilent Technologies, Inc. 2017

E6: GK44-ASB-8-10-17-RNA-PA14-w-BIT-24-0-1F-3

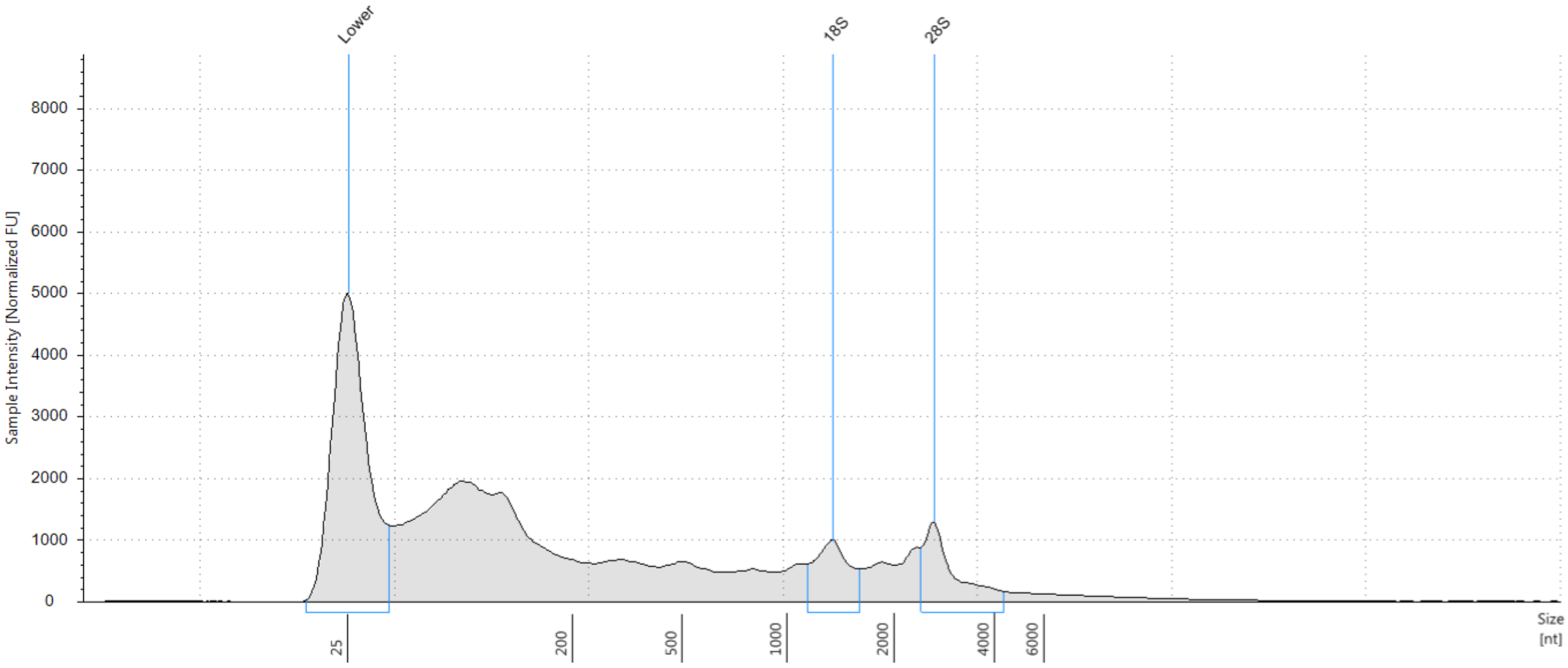

Sample Table

Well	RINe	28S/18S (Area)	Conc. [ng/µl]	Sample Description	Alert	Observations
E6	4.8	1.1	54.1	GK44-ASB-8-10-17-RNA-PA14-w-BIT-24-0-1F-3		

Peak Table

Size [nt]	Calibrated Conc. [ng/µl]	Assigned Conc. [ng/µl]	Peak Molarity [nmol/l]	% Integrated Area	Peak Comment	Observations
25	40.0	40.0	4710	-		Lower Marker
1343	4.06	-	8.88	47.35		18S
2641	4.51	-	5.02	52.65		28S

F6: GK45-ASB-8-30-17-RNA-PA14-w-BIT-24-0-1A-2

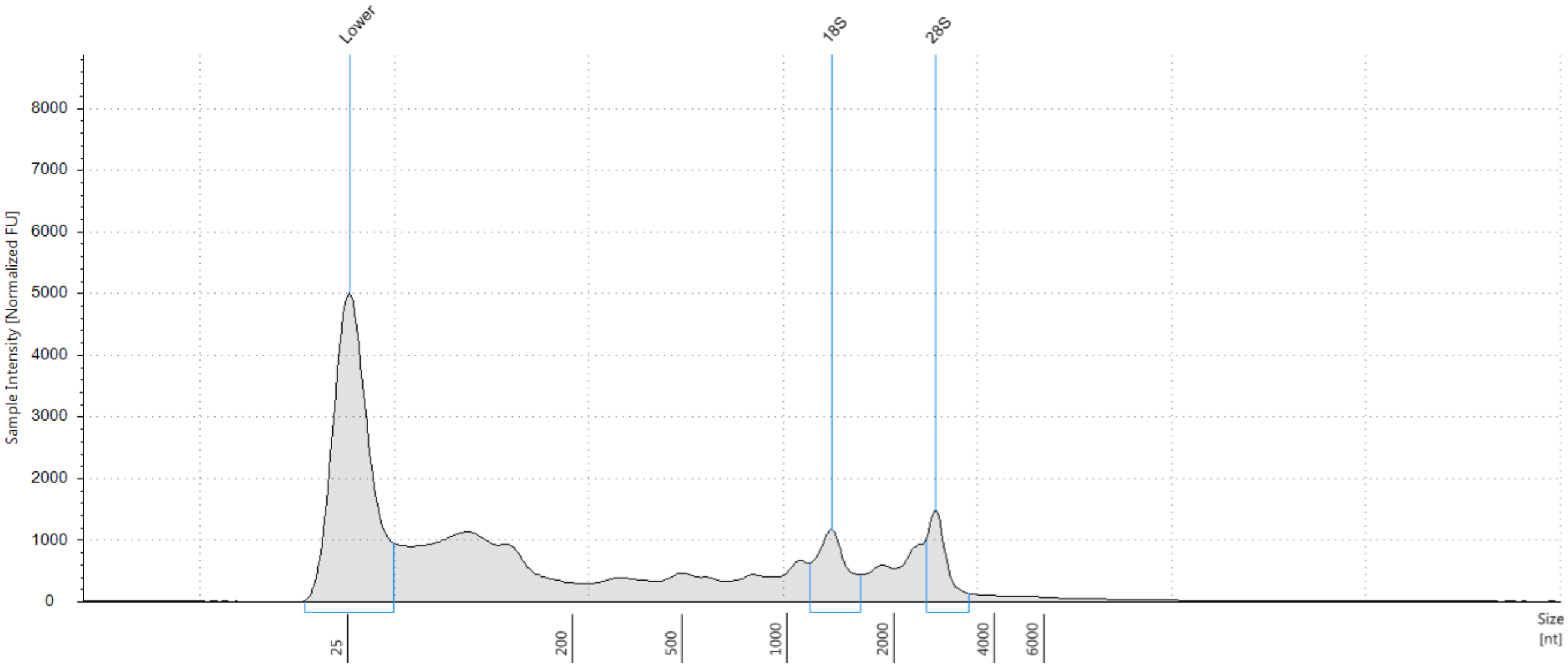

Sample Table

Well	RINe	28S/18S (Area)	Conc. [ng/µl]	Sample Description	Alert	Observations
F6	5.9	0.8	35.6	GK45-ASB-8-30-17-RNA-PA14-w-BIT-24-0-1A-2		

Peak Table

Size [nt]	Calibrated Conc. [ng/µl]	Assigned Conc. [ng/µl]	Peak Molarity [nmol/l]	% Integrated Area	Peak Comment	Observations
25	40.0	40.0	4710	-		Lower Marker
1327	3.96	-	8.77	55.61		18S
2648	3.16	-	3.51	44.39		28S

 © Agilent Technologies, Inc. 2017

G6: GK46-ASB-8-30-17-RNA-PA14-w-BIT-24-0-1B-2

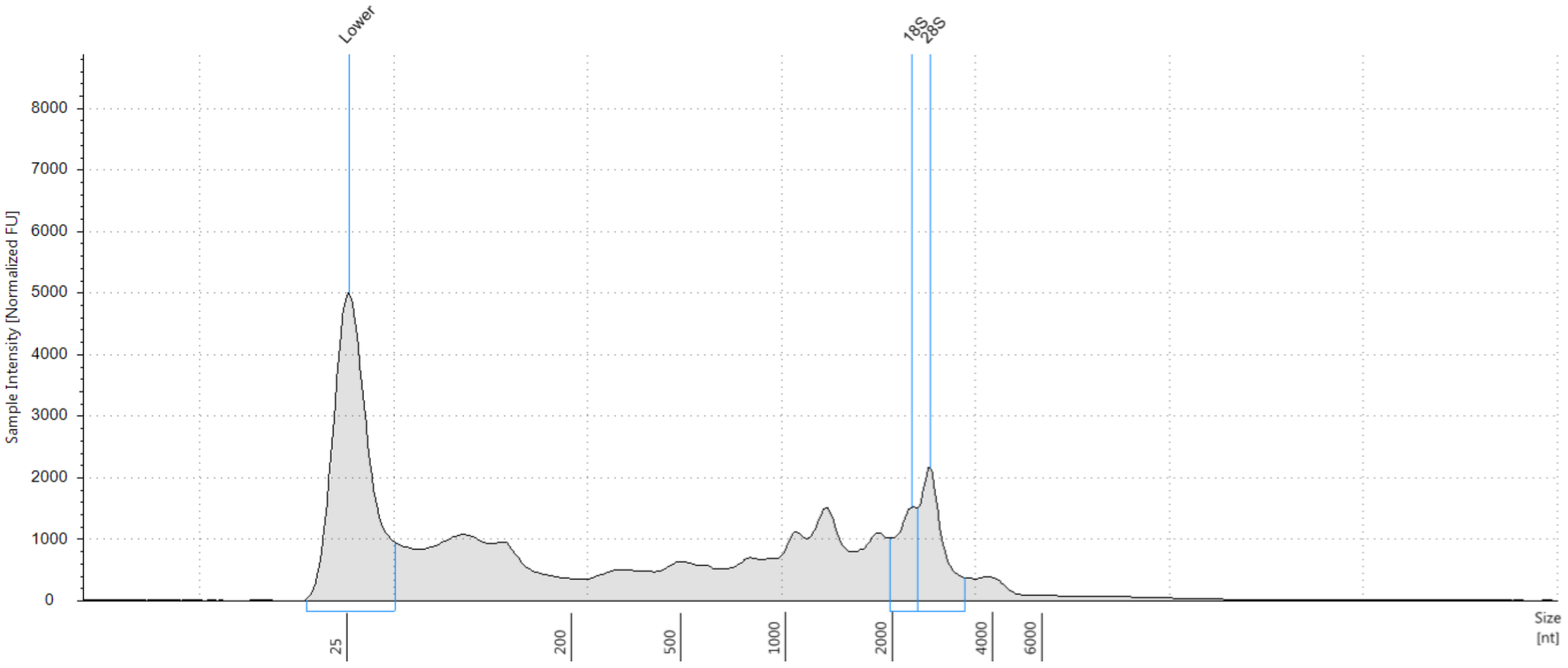

Sample Table

Well	RINe	28S/18S (Area)	Conc. [ng/µl]	Sample Description	Alert	Observations
G6	3.9	1.5	48.2	GK46-ASB-8-30-17-RNA-PA14-w-BIT-24-0-1B-2		

Peak Table

Size [nt]	Calibrated Conc. [ng/µl]	Assigned Conc. [ng/µl]	Peak Molarity [nmol/l]	% Integrated Area	Peak Comment	Observations
25	40.0	40.0	4710	-		Lower Marker
2281	3.69	-	4.76	39.55		18S
2584	5.64	-	6.42	60.45		28S

 © Agilent Technologies, Inc. 2017

H6: GK47-ASB-8-30-17-RNA-PA14-w-BIT-24-0-1D-2

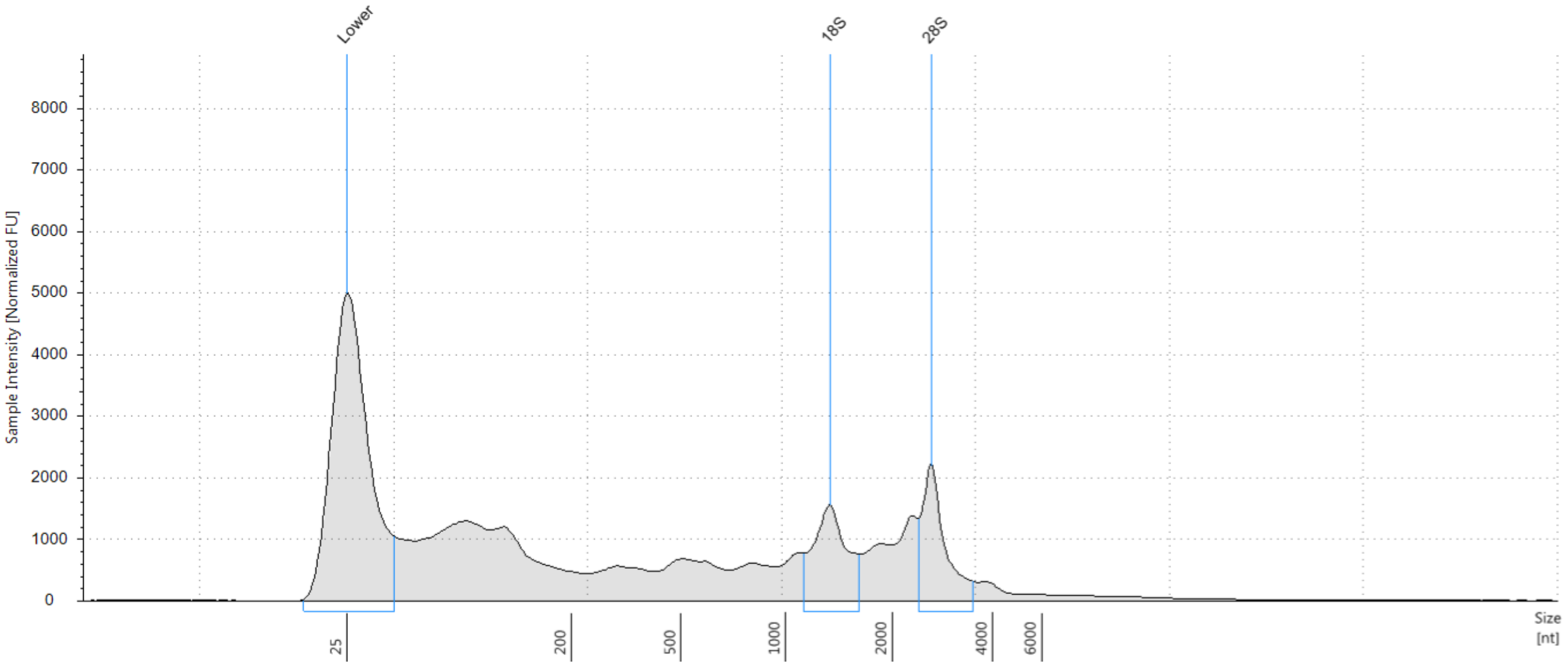

Sample Table

Well	RINe	28S/18S (Area)	Conc. [ng/µl]	Sample Description	Alert	Observations
H6	5.8	1.0	47.7	GK47-ASB-8-30-17-RNA-PA14-w-BIT-24-0-1D-2		

Peak Table

Size [nt]	Calibrated Conc. [ng/µl]	Assigned Conc. [ng/µl]	Peak Molarity [nmol/l]	% Integrated Area	Peak Comment	Observations
25	40.0	40.0	4710	-		Lower Marker
1339	5.72	-	12.6	50.29		18S
2627	5.65	-	6.33	49.71		28S

 © Agilent Technologies, Inc. 2017

A7: GK48-ASB-8-30-17-RNA-PA14-w-BIT-24-0-1F-2

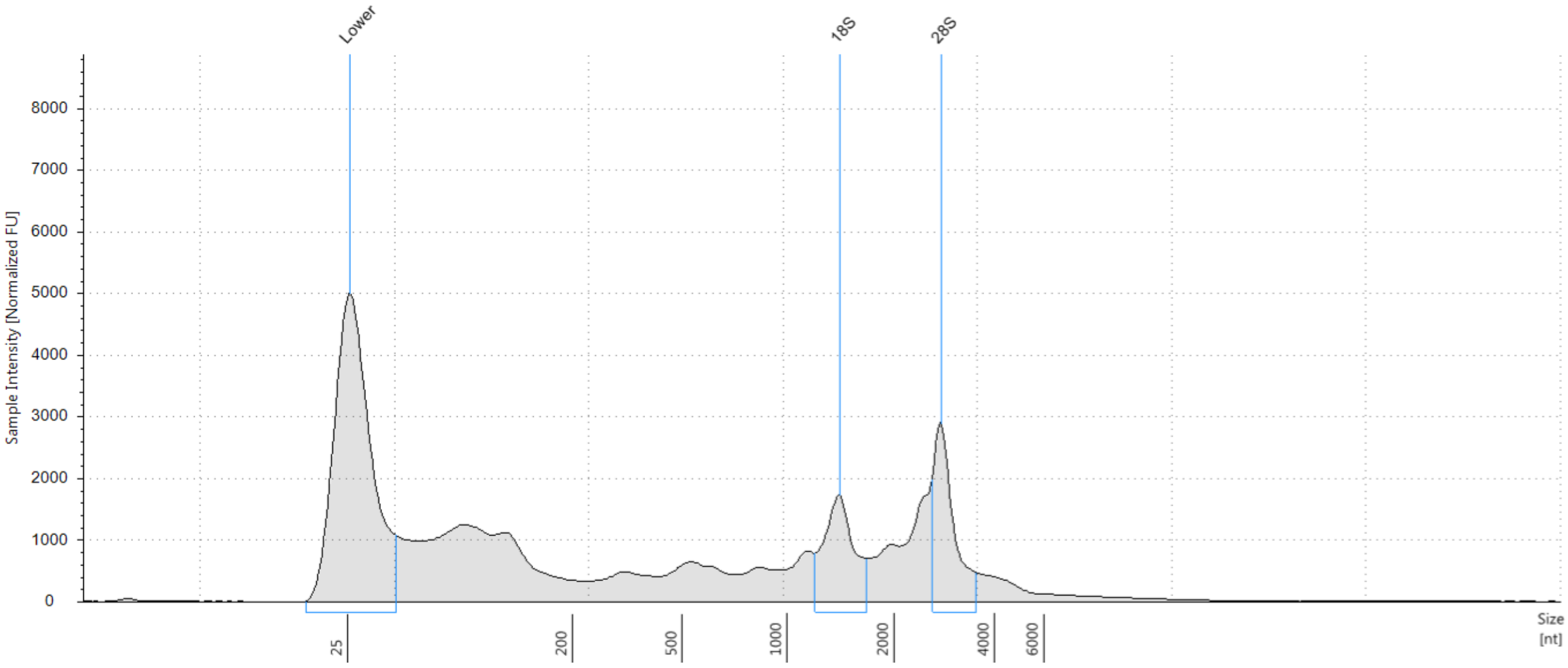

Sample Table

Well	RINe	28S/18S (Area)	Conc. [ng/µl]	Sample Description	Alert	Observations
A7	6.4	1.1	48.9	GK48-ASB-8-30-17-RNA-PA14-w-BIT-24-0-1F-2		

Peak Table

Size [nt]	Calibrated Conc. [ng/µl]	Assigned Conc. [ng/µl]	Peak Molarity [nmol/l]	% Integrated Area	Peak Comment	Observations
25	40.0	40.0	4710	-		Lower Marker
1397	5.74	-	12.1	47.30		18S
2737	6.39	-	6.87	52.70		28S

 © Agilent Technologies, Inc. 2017
