# Supplementary material for: Integrating multi-omics data with network modeling to characterize Pseudomonas aeruginosa persister cell metabolism
Source: J Bacteriol. 2026 Jul 20;208(8):e00373-25. doi: 10.1128/jb.00373-25 (PMC13492110; doi:10.1128/jb.00373-25)
Supplement: Supplementary file 3 [file jb.00373-25-s0003.pdf]

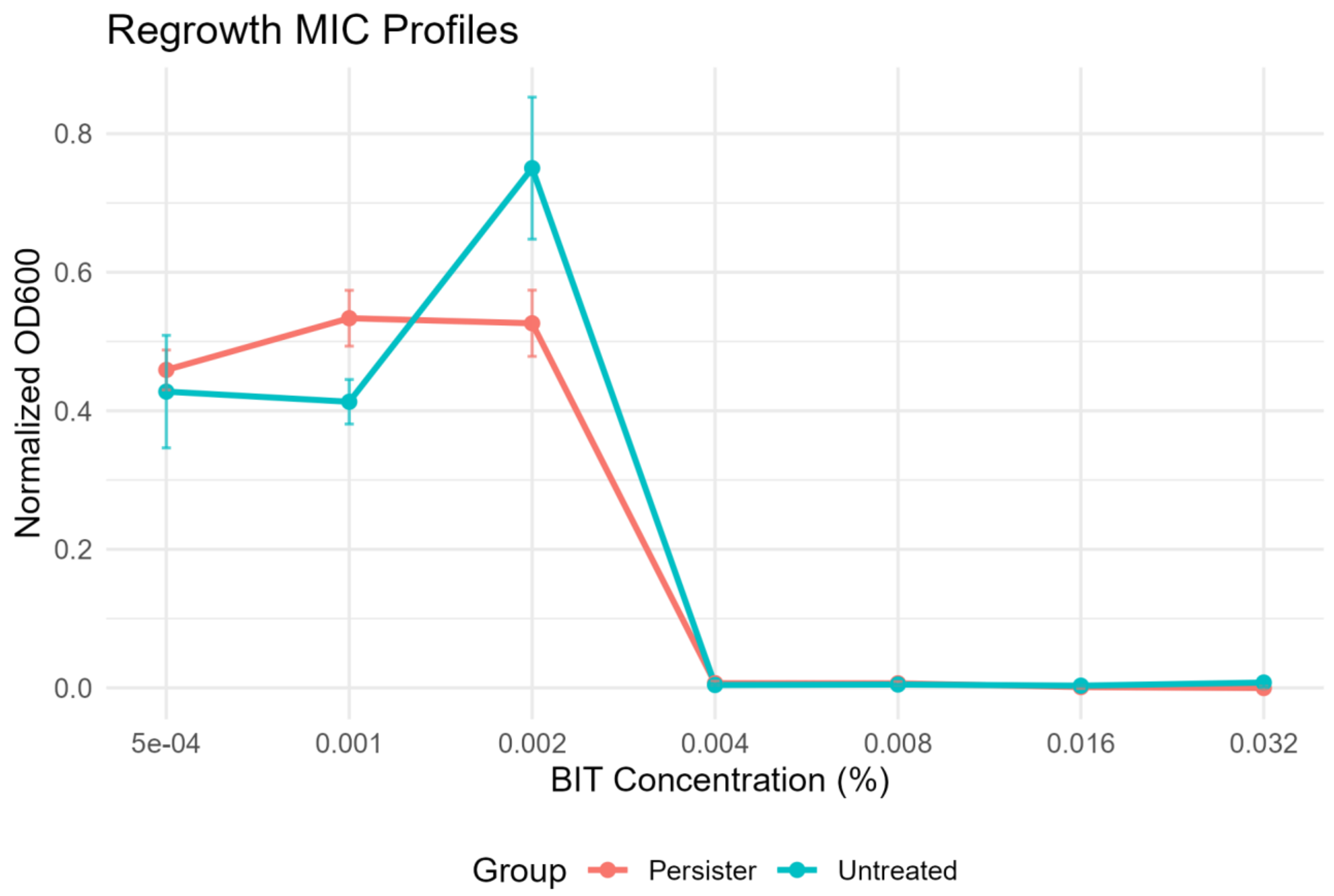

Figure S1. Dose response curve of re-grown *P. aeruginosa* cultures treated with BIT after the initial persister experiment. Overnight cultures of PA14 were diluted and exposed to various concentrations of BIT. Growth was measured 24h after inoculation via optical density (OD600) measurements.

A.

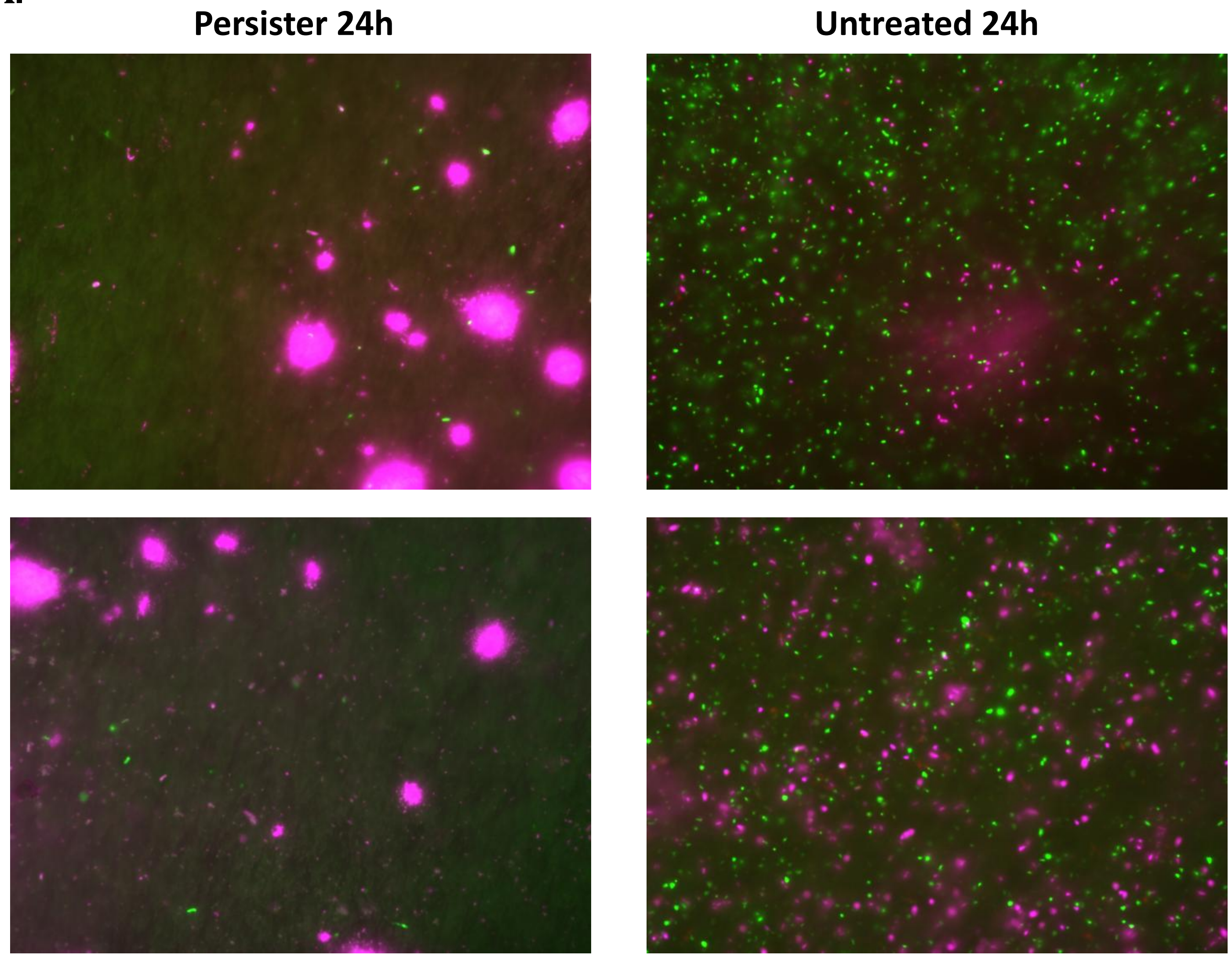

B.

	Live/Dead Ratio	Cell Area (pixels)
Persister	0.116	**Live:** 39.2 ± 38.3<br>**Dead:** 37.8 ± 24.2
Untreated	1.217	**Live:** 62.4 ± 41.3<br>**Dead:** 83.3 ± 70.8

Figure S2. (A) Example microscope images of persister cultures and untreated cultures at 24 hours. (B) Statistics on the live/dead ratio of cells in those imaged as well as the average cell areas and standard deviations.

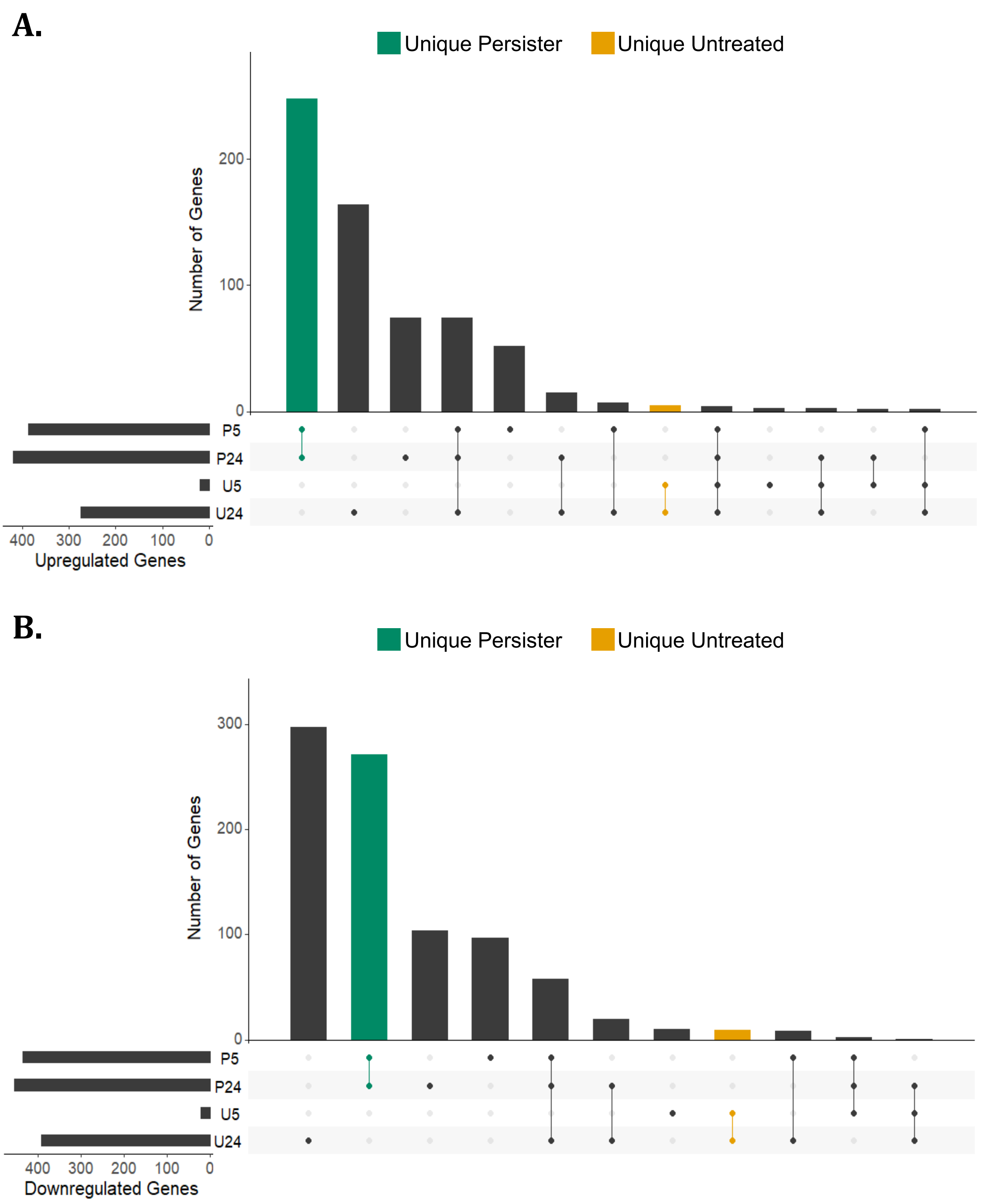

Figure S3. Comparison of upregulated (A) and downregulated (B) genes relative to their 0 hour counterparts .

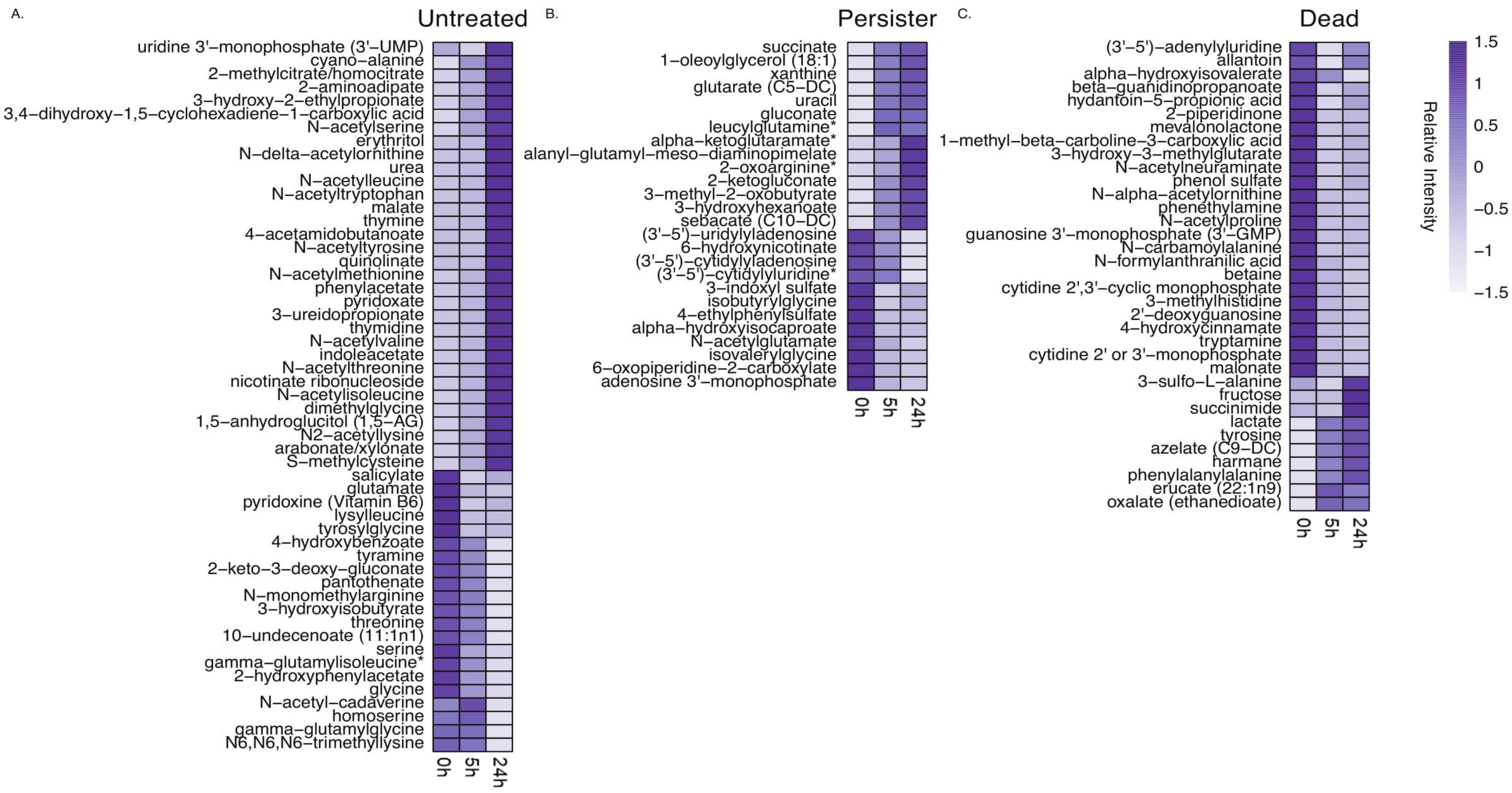

Figure S4. Metabolites that significantly change in abundance over time unique to the untreated (A), persister (B), and dead (C) conditions.

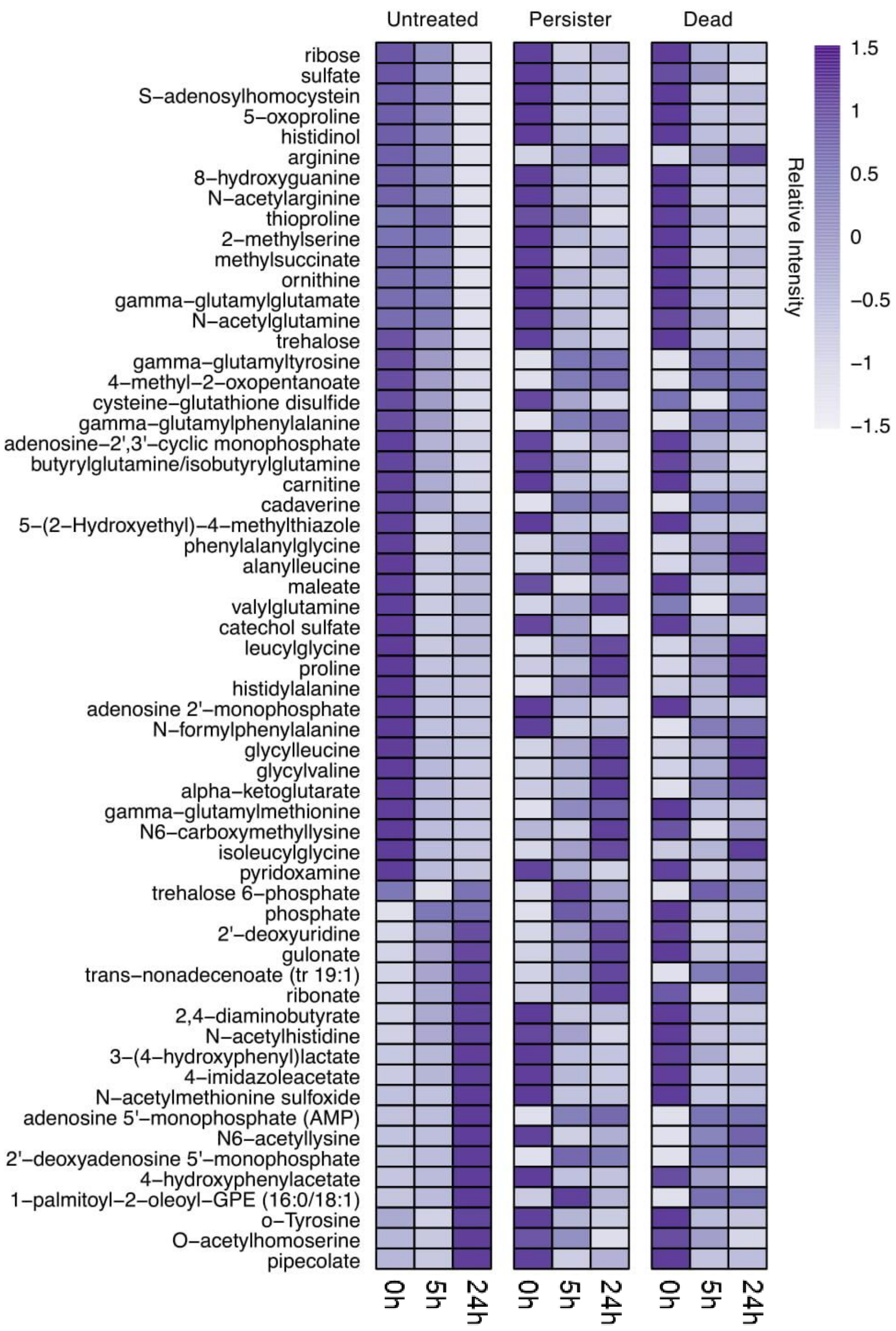

Figure S5. Metabolites that significantly change in abundance over time common to the untreated, persister, and dead conditions.

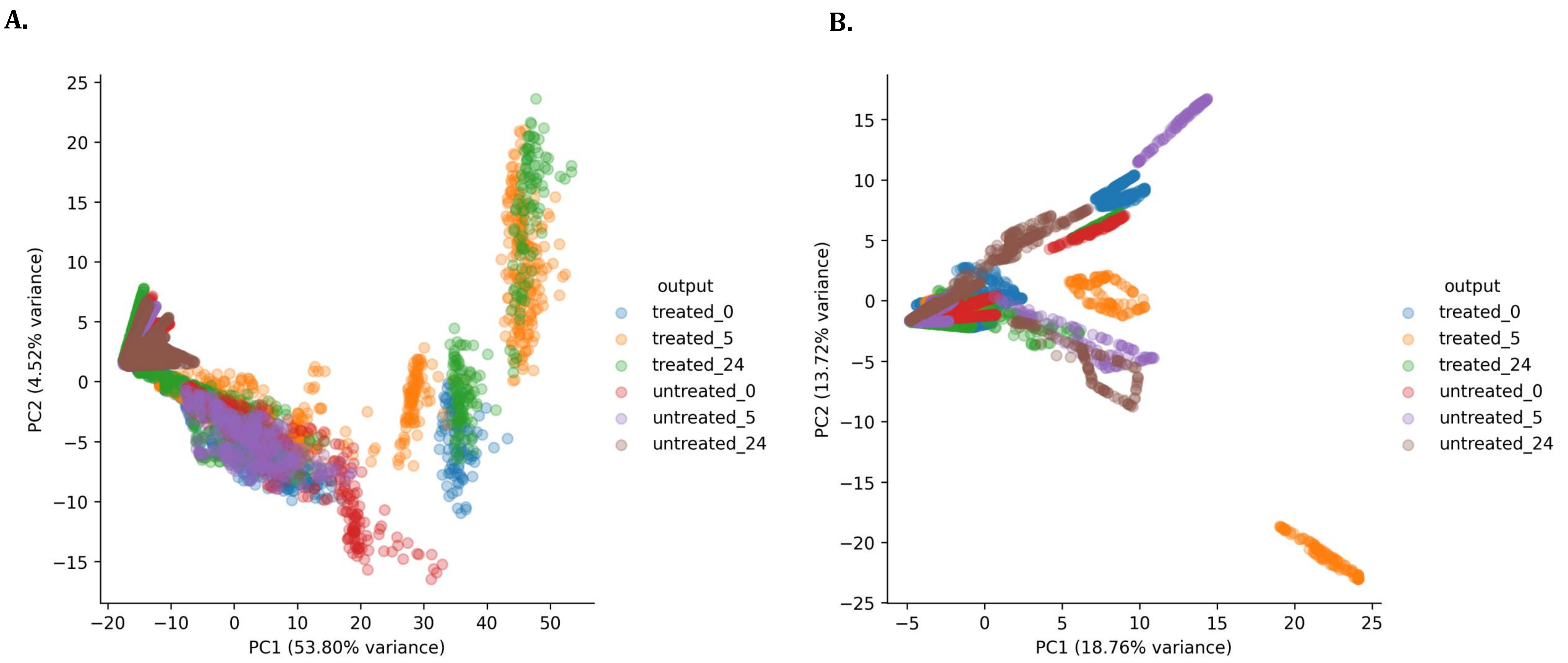

Figure S6. PCA plot from sampling the flux distributions of the context-specific metabolic models optimized for (A) biomass and (B) ATP production.

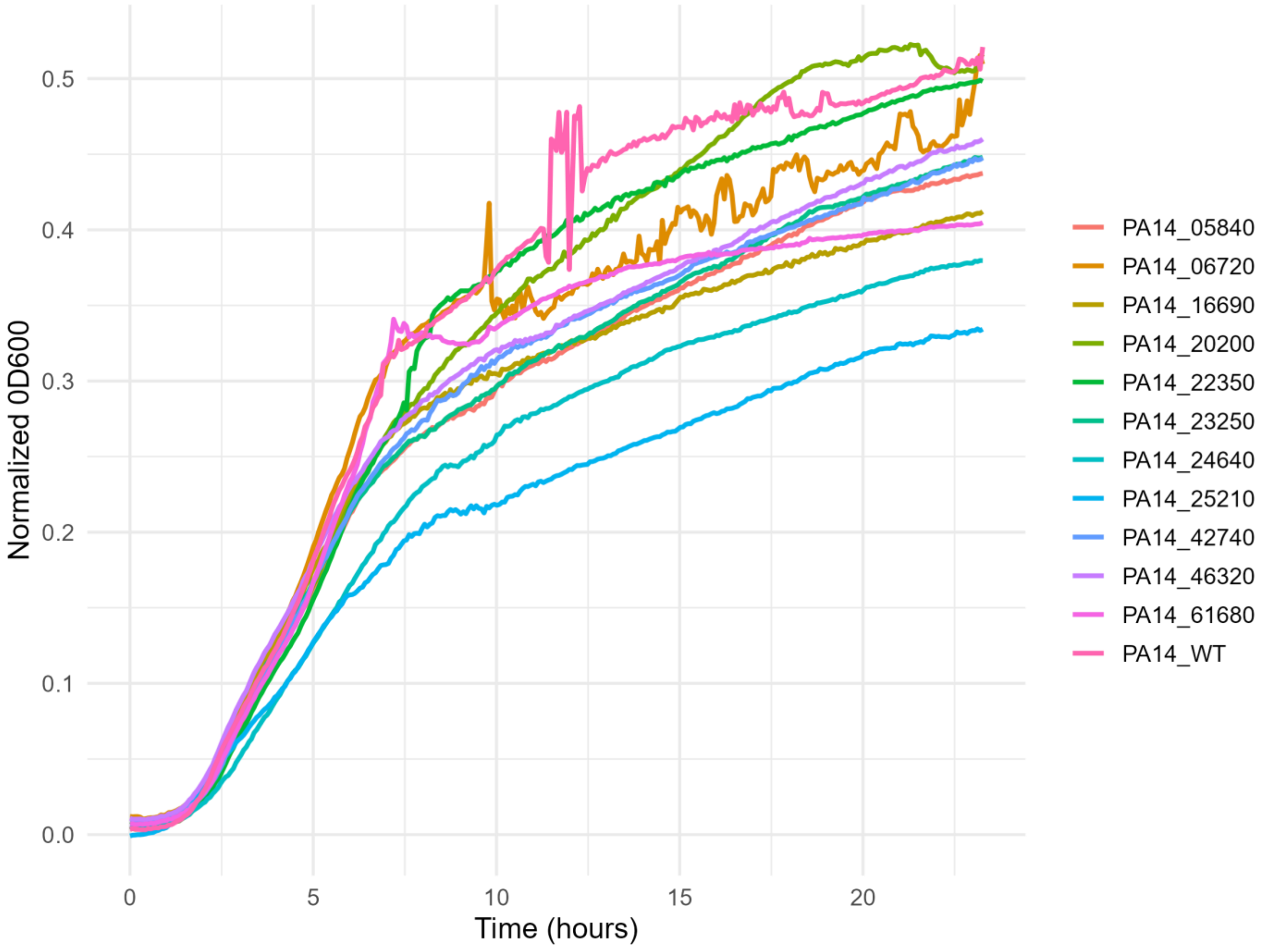

Figure S7. Growth curves of each of the PA14 mutants used in the mutant validation experiment.

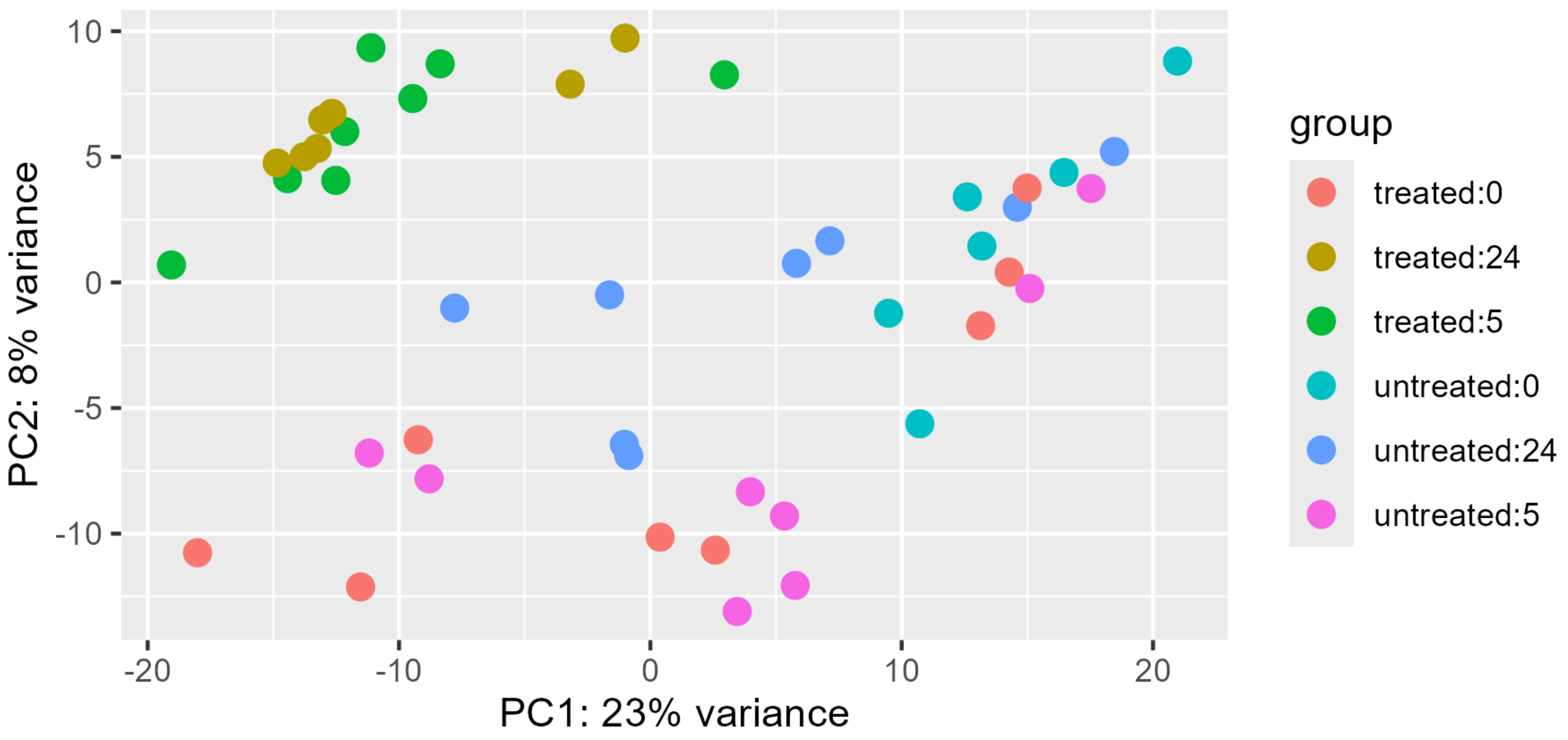

Figure S8. Principal component analysis (PCA) of variance-stabilized RNA-seq counts, showing samples colored by condition, with batch effects accounted for in the DESeq2 design.

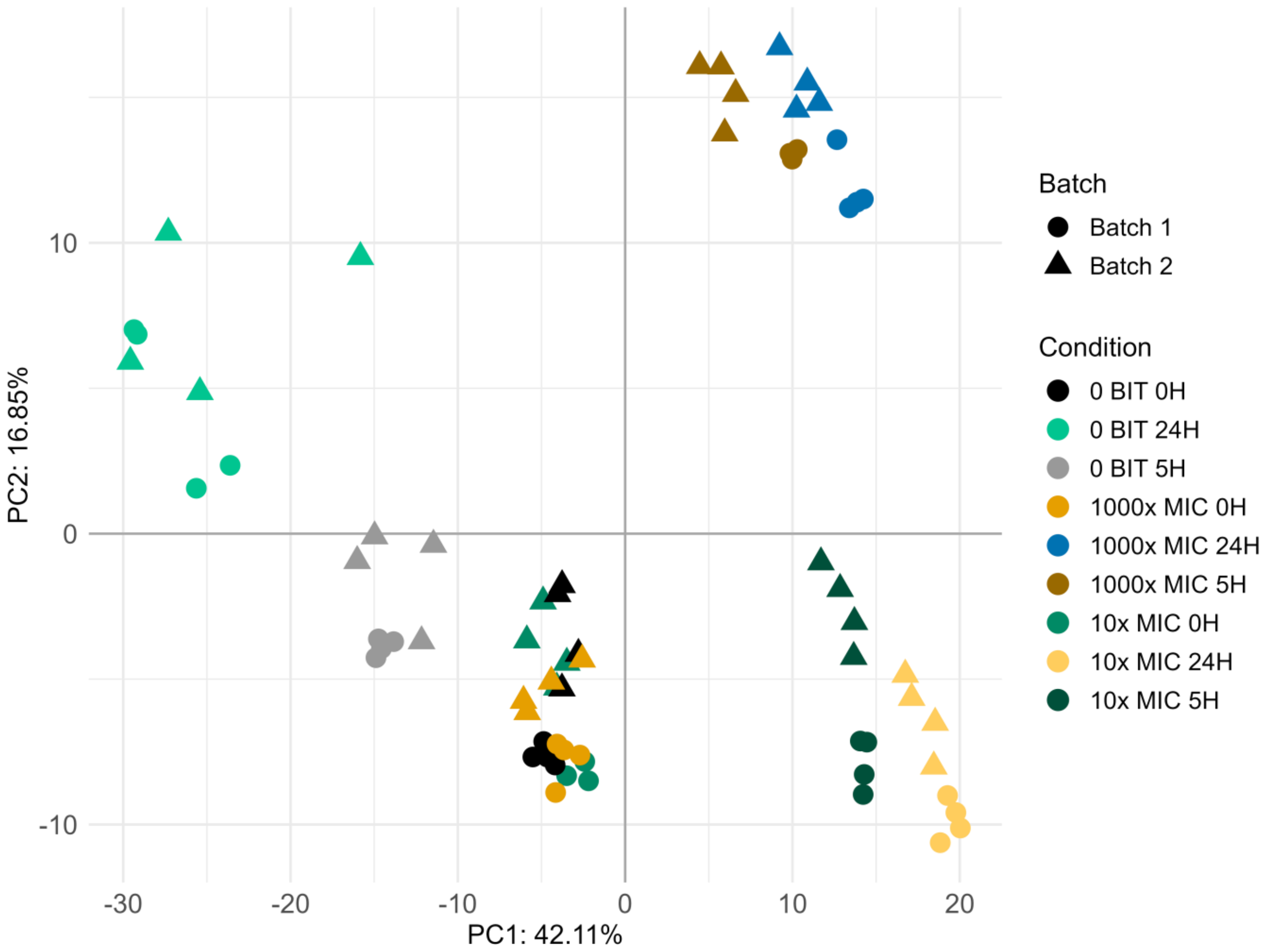

Figure S9. Principal component analysis (PCA) of normalized metabolomics samples.
